# Mycobacteria that cause tuberculosis have retained ancestrally acquired genes for the biosynthesis of chemically diverse terpene nucleosides

**DOI:** 10.1371/journal.pbio.3002813

**Published:** 2024-09-30

**Authors:** Jacob A. Mayfield, Sahadevan Raman, Alexandrea K. Ramnarine, Vivek K. Mishra, Annie D. Huang, Sandrine Dudoit, Jeffrey Buter, Tan-Yun Cheng, David C. Young, Yashodhan M. Nair, Isobel G. Ouellet, Braden T. Griebel, Shuyi Ma, David R. Sherman, Ludovic Mallet, Kyu Y. Rhee, Adriaan J. Minnaard, D. Branch Moody

**Affiliations:** 1 Division of Rheumatology, Inflammation and Immunity, Brigham and Women’s Hospital, Harvard Medical School, Boston, Massachusetts, United States of America; 2 Stratingh Institute for Chemistry, University of Groningen, Groningen, the Netherlands; 3 Division of Biostatistics, School of Public Health, University of California, Berkeley, California, United States of America; 4 Department of Statistics, University of California, Berkeley, California, United States of America; 5 University of Washington Department of Chemical Engineering, Seattle, Washington State, United States of America; 6 Center for Global Infectious Disease Research, Seattle Children’s Research Institute, Seattle, Washington State, United States of America; 7 University of Washington Department of Pediatrics, Seattle, Washington State, United States of America; 8 University of Washington Pathobiology Program, Department of Global Health, Seattle, Washington State, United States of America; 9 Department of Microbiology, University of Washington, Seattle, Washington State, United States of America; 10 Unité de Mathématique et Informatique Appliquées de Toulouse, INRA, Castanet-Tolosan, France; 11 Division of Infectious Diseases, Weill Department of Medicine, Weill Cornell Medical College, New York, New York, United States of America; Universitat zu Koln, GERMANY

## Abstract

*Mycobacterium tuberculosis* (Mtb) releases the unusual terpene nucleoside 1-tuberculosinyladenosine (1-TbAd) to block lysosomal function and promote survival in human macrophages. Using conventional approaches, we found that genes *Rv3377c* and *Rv3378c*, but not *Rv3376*, were necessary for 1-TbAd biosynthesis. Here, we introduce linear models for mass spectrometry *(limms)* software as a next-generation lipidomics tool to study the essential functions of lipid biosynthetic enzymes on a whole-cell basis. Using *limms*, whole-cell lipid profiles deepened the phenotypic landscape of comparative mass spectrometry experiments and identified a large family of approximately 100 terpene nucleoside metabolites downstream of *Rv3378c*. We validated the identity of previously unknown adenine-, adenosine-, and lipid-modified tuberculosinol-containing molecules using synthetic chemistry and collisional mass spectrometry, including comprehensive profiling of bacterial lipids that fragment to adenine. We tracked terpene nucleoside genotypes and lipid phenotypes among *Mycobacterium tuberculosis* complex (MTC) species that did or did not evolve to productively infect either human or nonhuman mammals. Although 1-TbAd biosynthesis genes were thought to be restricted to the MTC, we identified the locus in unexpected species outside the MTC. Sequence analysis of the locus showed nucleotide usage characteristic of plasmids from plant-associated bacteria, clarifying the origin and timing of horizontal gene transfer to a pre-MTC progenitor. The data demonstrated correlation between high level terpene nucleoside biosynthesis and mycobacterial competence for human infection, and 2 mechanisms of 1-TbAd biosynthesis loss. Overall, the selective gain and evolutionary retention of tuberculosinyl metabolites in modern species that cause human TB suggest a role in human TB disease, and the newly discovered molecules represent candidate disease-specific biomarkers.

## Abstract 2

Mycobacterium tuberculosis releases 1-tuberculosinyladenosine to block lysosomal function and promote survival in human macrophages. This study identifies over 100 such terpene nucleoside metabolites, revealing how ancestral M. tuberculosis complex bacteria acquired the genes needed to make these unique bioactive lipids.

## Introduction

Whereas most mycobacterial species are nonpathogenic or infect nonhuman hosts, *Mycobacterium tuberculosis* (Mtb) is an obligate human pathogen that causes lung disease on a worldwide basis, killing more than 1 million people per year. The lipid-rich mycobacterial envelope contributes to the global burden of tuberculosis (TB) disease as major source of phenotypic variance and virulence factors. In addition to forming the primary barrier with the host, mycobacterial lipids carry out specific functions that induce cough [1], moderate immunity [2], and mediate antibiotic resistance [3]. Mass spectrometry has revealed thousands of mycobacterial lipids organized into 58 classes [4], emphasizing the extreme complexity of its evolved lipidome, but also providing a path to new pathogen-shed diagnostics and drug targets.

One recently discovered *Mycobacteria*-restricted lipid is the lysosomotropic base 1-tuberculosinyladenosine (1-TbAd), which comprises >1% of total Mtb lipid [5]. The tandem gene*s Rv3377c* and *Rv3378c* encode 1-TbAd biosynthesis. Their atypical GC-content and lack of orthology suggested horizontal gene transfer from an undetermined source [6], a hypothesis later extended to include the adjacent gene *Rv3376* [7]. Chemical and genetic investigations showed that *Rv3378c* encodes the tuberculosinyl transferase that generates 1-TbAd [5], the rearrangement product *N*^*6*^-TbAd [8], and the by-product isotuberculosinol [7]. *Rv3377c* is presumed to encode a synthase for the unusual halimane lipid tuberculosinol pyrophosphate [7], while *Rv3376* encodes a haloacid dehydrogenase ortholog with an unknown role. Only the function of *Rv3378c* has been directly determined, and the breadth of molecules made by this enzyme remains unknown. Further, the origin, regulation, and function of this putative locus among mycobacteria that vary in virulence and human tropism remain unknown.

The 1-TbAd biosynthetic genes can influence mycobacterial survival in cells by inhibiting lysosomal acidification [9,10], which promotes pathogen survival in mouse macrophages [6,11] and in lungs during early infection in vivo [12]. These functional data align with the known chemical mechanism of 1-TbAd to act as a weak base [10] and with data showing that *Rv3378c* or 1-TbAd has an essential role in blocking lysosomal maturation and autophagy, which are 2 cellular processes involved in escape from host killing [11,13,14]. However, the locus is not essential for infection, as suggested by gene silencing experiments with mixed Mtb strains [15]. One potential explanation for all data is that tuberculosinyl metabolites are decisive for infection outcomes in certain circumstances, like persistence through nutrient limitation in macrophages, which 1-TbAd was recently shown to influence [11]. Mice have limited ability to model the early survival of single bacteria, transmission, and persistence events that occur during human tuberculosis disease, highlighting the need for human data to understand possible roles of 1-TbAd in virulence. Mycobacterial pathogens with competence for infection of mammals appeared in the MTC through evolution from nonvirulent soil *Mycobacteria*, with only a subset further disseminating among humans as epidemic TB disease. Therefore, we asked if 1-TbAd biosynthesis gene variations among MTC species that occurred over the same time frame as acquisition of the capability for productive infection of humans could offer clues to TB disease [16,17].

Our central goals were to determine the origin, evolutionary timing, transfer mechanism, and biochemical outputs of the known tuberculosinyl biosynthetic locus in *Mycobacteria*. However, the complexity of mass spectrometry-based experiments comparing multiple strains and species required advancement of bioinformatic tools for comparative metabolomics. The output of a modern mass spectrometer is a list of ion masses, retention times, and peak intensities that can exceed 10,000 multidimensional data points (molecular events). Hence, comparative experiments incur a substantial multiple hypothesis testing penalty. Further, peak finding algorithms are prone to artifacts for events near the threshold of detection that distort statistical testing by introducing intermittent zero values. Comparisons such as nested samples, paired sample analysis, dose responses, and time courses have high discovery potential but are best analyzed using contrast-based methods instead of pairwise testing. Extending the first generation comparative lipidomics platform for two-way analysis [4], here we introduce *limms* as a second generation profiling tool for differential abundance analysis using flexible contrast-based comparisons, linear models, and Bayesian shrinkage of variance [18]. Using *limms*, we identified an unexpected and large family of approximately 100 previously unknown tuberculosinyl compounds. Further, combining sequence analysis and chemotyping, we identified the likely origin and timing of horizontal transfer of the locus, which revealed that gain of constitutively high 1-TbAd biosynthesis correlated with acquisition of human TB causation on an evolutionary time scale.

## Results

### Targeted analysis of 1-TbAd biosynthesis gene functions

Given 1-TbAd’s ability to block lysosome function in macrophages and promote mycobacterial growth in macrophage culture [6,11] and in vivo [12], we sought to understand more about 1-TbAd production by testing the functions of all 3 biosynthetic genes using targeted knockouts. Knowing *Rv3378c* is essential for 1-TbAd production [10], here we deleted *Rv3376* and *Rv3377c* through gene replacement, as well as creating a double mutant of *Rv3377c* and *Rv3378c*. Strains were validated through sequencing and RT-PCR (S1A–S1F Fig) and shown to not alter growth in 7H9 media (S1G Fig). While we complemented the *Rv3378c* and *Rv3377c-Rv3378c* double deletions, all attempts to complement the *Rv3376* or *Rv3377c* deletion strains failed. We hypothesized non-native expression of these genes was genotoxic.

**Fig 1:**
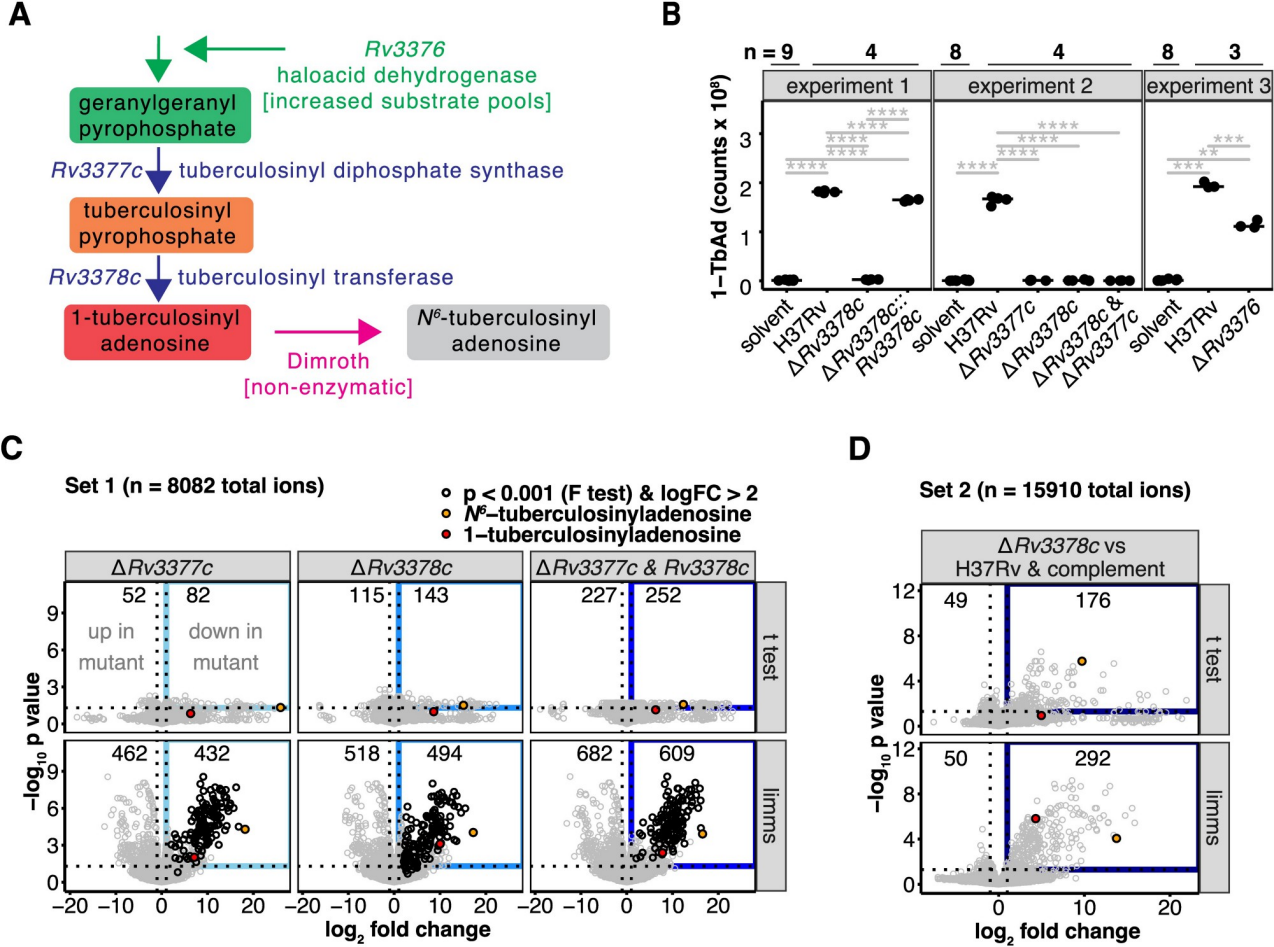
Engineered deletions of 1-TbAd biosynthesis genes reveal gene functions and greatly expand the lipid signature. (A) Schematic shows the 1-TbAd biosynthetic pathway. (B) Area-under-the-curve of extracted ion chromatograms tested 1-TbAd production by the parental Mtb strain (H37Rv) and single or two-gene knockouts as well as the *Rv3378c* deletion complemented with *Rv3378c*. A Benjamini–Hochberg adjusted *p* value is indicated only for significant pairwise *t* tests (*: *p* < 0.05, **: *p* < 0.01, ***: *p* < 0.001, ****: *p* < 0.0001). The peak area in the retention time window corresponding to Mtb H37Rv 1-TbAd [M+H]^+^ was measured in intervening solvent blank samples to indicate the measurement threshold. (C) Comparative metabolomics analysis showed genetic control of differentially abundant molecules. Positive mode mass spectrometry data were analyzed by comparing deletions in *Rv3377c*, *Rv3378c*, or a double deletion of both genes to the H37Rv parental strain. Differential abundance determined using *t* tests or a linear model fit using *limms* was compared. The number of significant events (*p* < 0.05 after adjustment using the Benjamini–Hochberg method) that also changed more than 2-fold were indicated (blue rectangle). The most abundant 1- and *N*^*6*^-tuberculosinyladenosine peaks are flagged (red and orange circles, respectively). Using *limms*, events with similar patterns of change in all 3 comparisons were determined by F-test. Events with >4-fold decrease in all 3 mutants and *p* < 0.001 (Benjamini–Hochberg adjusted *p* value of the F-test) are shown in black. (D) An independent metabolomic comparison of the *Rv3378c* deletion to H37Rv parent strain and the *Rv3378c* deletion complemented with *Rv3378c* was analyzed for differentially abundant positive mode events. Significantly changed events were determined using *t* tests or *limms*. The numbers of changed events (Benjamini–Hochberg adjusted *p* < 0.05 and 2-fold or greater change), the gene-dependent events (blue rectangle), and the most abundant 1- and *N*^*6*^-tuberculosinyladenosine peaks are indicated as in Fig 2A. The data in Fig 1B–1D can be found in S1 Data.

Rv3377c and Rv3378c are thought to act sequentially, producing tuberculosinyl pyrophosphate and conjugating it to adenosine, respectively (Fig 1A, red) [5,7]. Targeted mass spectrometry detected 1-TbAd ([M+H]^+^ for TbAd and subsequent terpene nucleosides) and its rearrangement product *N*^6^-TbAd in parental Mtb H37Rv strains. *Rv3377c* was indeed necessary for both TbAd forms (Fig 1B). While failure to complement the *Rv3377c* deletion meant a second site effect was not ruled out, we noted that isolates with 2 different loss-of-function alleles in *Rv3377c* were also defective in 1-TbAd production [10]. In contrast, deletion of *Rv3376* reduced but did not eliminate 1-TbAd, ruling out an essential biosynthetic function but consistent with an accessory role (Figs 1B and S2). Nakano [19] demonstrated Rv3376 has phosphatase activity, which might augment geranylgeranyl pyrophosphate pools (Fig 1A, green).

### *limms* for untargeted metabolomics

Whereas conventional approaches measure the effect of gene deletion on expected or known products of enzymes (Fig 1A and 1B), lipidomics platforms enable untargeted approaches to measure the scope of effects on the organism that includes unknown molecules measured as percent of total lipids meeting defined change criteria (Fig 1C and 1D). To achieve this phenotypic expansion, comparative lipidomics relies on differential abundance, linking the mass, retention time, and intensity values of unnamed “molecular events” [4] to genetic or conditional effects. This untargeted approach can discover previously unknown compounds, connect chemicals to biosynthetic genes lacking known substrates or products, and link metabolites to unexpected or emergent networks. However, this approach applied to high-resolution mass spectrometry data creates a large multiple hypothesis testing problem: comparing lipid extracts from the parental, single and double knockouts in *Rv3377c* and *Rv3378c* generated 24,300 events to test in multi-way comparisons. Furthermore, mass spectrometry event lists are not immediately amenable to statistical analysis pipelines for identifying differential abundance because of intermittent zero intensities and technical variability.

Therefore, we wrote the open-source R package, *limms*, to overcome these limitations and support a next-generation metabolomics platform. This software normalizes and imputes mass spectrometry data, facilitates contrast-based statistical comparisons [20], applies *p* value adjustments [20], and supports data visualization (S2 Data). Unlike prior approaches that are constrained to pairwise or all-ways comparisons [4,21,22], *limms* allowed flexible specification of multi-way contrasts, including a three-way complementation (Fig 1C) and four-way epistasis analysis (Fig 1D); furthermore, paired samples, nested contrasts, time courses, and dose-response analyses are accepted with their use explained in the *limms* vignette (S2 Data). *limms* works with data from any mass spectrometry platform, chromatography system, and type of metabolite. Broadly applicability was demonstrated by reanalysis of previously published data that measured intracellular metabolites from *Saccharomyces cerevisiae* using a different LC-MS system [23]. Changes in sulfur-containing amino acids were expected when yeast lacking cystathionine beta-synthase (CBS) activity were trans-complemented with human alleles; however, analysis using *limms* found statistically significant changes extended well beyond the CBS pathway (S3 Fig, S1 Table, and S2 Data). These data are included and utilized as examples in the *limms* vignette (included as S2 Data) and help pages.

### *limms* revealed an unexpected lipidomic phenotype

Using *limms*, lipidomics analysis of all events from the Mtb genetic studies focused on the genetically controlled metabolites whose intensity values changed at least 2-fold with *p* value <0.05 (Fig 1C and 1D and S3 Data). Because 1-TbAd and *N*^*6*^-TbAd were the only known products of this locus (Fig 1A), the large number of changed events after deletion of *Rv3377c* (894 events) or *Rv3378c* (1,012 events), or double deletion (1,291 events) was highly unexpected (Fig 1C). Mass-retention time addresses of changed events showed high overlap in all 3 mutants (Fig 1C; F-test *p* < 0.001 in S3 Data), consistent with the proposed pathway in Fig 1A, but did not clarify the order of gene action (Fig 1C). Separately, we compared the *Rv3378c* deletion mutant to the parental strain and complemented *Rv3378c* deletion, with complementation used to increase statistical stringency and address possible second-site mutations, and 292 events showed lower intensity when *Rv3378c* was deleted (Fig 1D, blue), while 50 up-regulated events showed small increases in intensity. Hence, 2 independent experiments showed a marked expansion of the lipid phenotype beyond 1-TbAd and *N*^*6*^-TbAd.

Comparative lipidomics without *limms* was possible by applying statistical methods piecemeal. For example, the R package *xcms* used here for peak picking and alignment [22], tabulated *p* values for pairwise *t* tests. However, in these cases the mass spectrometry data are not normalized, *p* values are not adjusted, and complex experimental designs can only be approached by looking for overlaps in data sets by non-statistical means like Venn diagrams. For comparisons of the results with and without *limms*, we applied pairwise *t* tests to the 2 mass spectrometry data sets in Fig 1C and 1D. More events were detected as differentially abundant using *limms* and many fewer events localized near the x-axis (high fold-change but *p* > 0.01), with the lead compounds 1- and *N*^*6*^-tuberculosinyladenosine being better differentiated. Only 52 events were significantly decreased in all 3 mutant strains when the overlap between *t* test results in Fig 1C was computed. In contrast, 194 events were found to be significantly decreased for the same data via the F-test in *limms*. Furthermore, the F-test provided a *p* value that directly addressed the hypothesis that the single and double mutants altered the same events. Mapping the events detected by F-test as most changed onto the individual mutant contrasts showed the decreased events occupied similar space in all 3 mutants (Fig 1C, black), consistent with almost identical lipid phenotypes for all 3 mutant strains.

### Lipidomic discovery of terpene nucleotides

**Fig 2:**
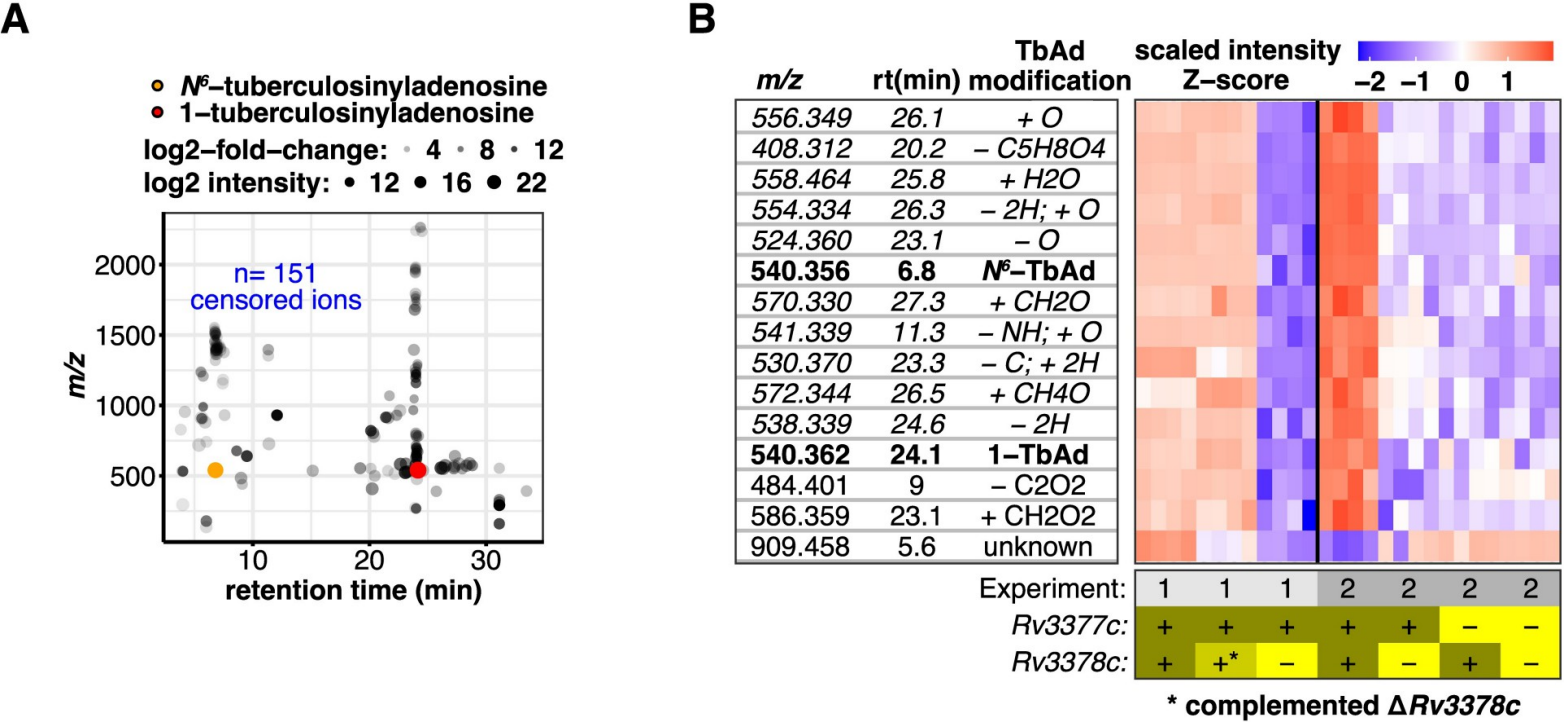
*Rv3377-3378c*-dependent lipids share biochemical properties consistent with a larger family of terpene nucleosides. (A) The 254 significantly changed events in the blue box in panel Fig 1D were filtered to remove 151 recognizable isotopes and alternate adducts, yielding 104 unique molecules. These *Rv3378c*-dependent events restored by complementation clustered in mass and time with either 1- and *N*^*6*^-TbAd. (B) Heatmap of 15 *Rv3378c*-dependent events that had mass (within 10 ppm) and retention time (within 3 min) analogs across the 2 independent experimental data sets shown in Fig 1C and 1D. Rows show the ion intensity scaled separately for each experiment. Chemical modifications to TbAd consistent with the observed mass are indicated. Rows in *italics* were subsequently validated. Raw data for Fig 2B and 2C can be found in S1 Data.

Censoring isotopes, alternative adducts, and multimers yielded a triaged list of 108 *Rv3378c*-dependent targets of unknown chemical structure from the 292 events identified in Fig 1D. The unknowns clustered around 1-TbAd (24 min) and *N*^*6*^-TbAd (7 min) in time, consistent with being chemically related to 1- and *N*^*6*^-linked purine structures (Fig 2A). In contrast to the events markedly reduced or lost after *Rv3378c* deletion, 50 events with increased intensity showed lower fold-change, suggestive of dispersed flux rather than activation of a specific pathway.

To prioritize high value targets, we further restricted candidates to changed events seen in both experiments (Fig 1C and 1D; significant F-test). Intersection of these data, intended to filter lipids with variable abundance due to culture state or nutrient availability, yielded 82 lipid targets, approximately 4.5 times more using *limms* than the 18 events overlapping in the *t* test sets. We hypothesized these were TbAd-like molecules and subtracted the exact mass of TbAd (540.354) from each unknown. Chemical modifications including deglycosylation, acetylation, reduction, oxidation, or others could account for many changed events (Fig 2B). One event (*m/z* 909.458) could not be linked to TbAd but had a bifurcated pattern of change in the 2 experiments, that while significant in both experiments, did not appear *Rv3378c* dependent. Some, like acetyl-TbAd, had been presumptively identified in culture medium or resembled natural or synthetic terpenes [7,19,24–27]. These unexpected results suggested that *Rv3378c* acts on multiple substrates, has promiscuous enzymatic activity, or that 1-TbAd undergoes previously unknown downstream modifications. Furthermore, identification of many distinct TbAd-like molecules suggested that prior measurements of 1-TbAd [8], despite the high absolute yield comprising 1% to 2% of total cellular lipids, underestimated the amount and diversity of lipids dependent on *Rv3378c*. Given recent successful efforts in MS detection of Mtb-specific metabolic biomarkers, including 1-TbAd, in human breath [28] and biofluids [29], the *Rv3378c*-linked *m/z* values had intrinsic biological value as endpoints even without further chemical validation. Nonetheless, we sought evidence for or against the modifications proposed in Fig 2B using a combination of techniques that subsequently generated data informing 10 modifications (Fig 2B, italics).

### Validation through synthetic chemistry

**Fig 3:**
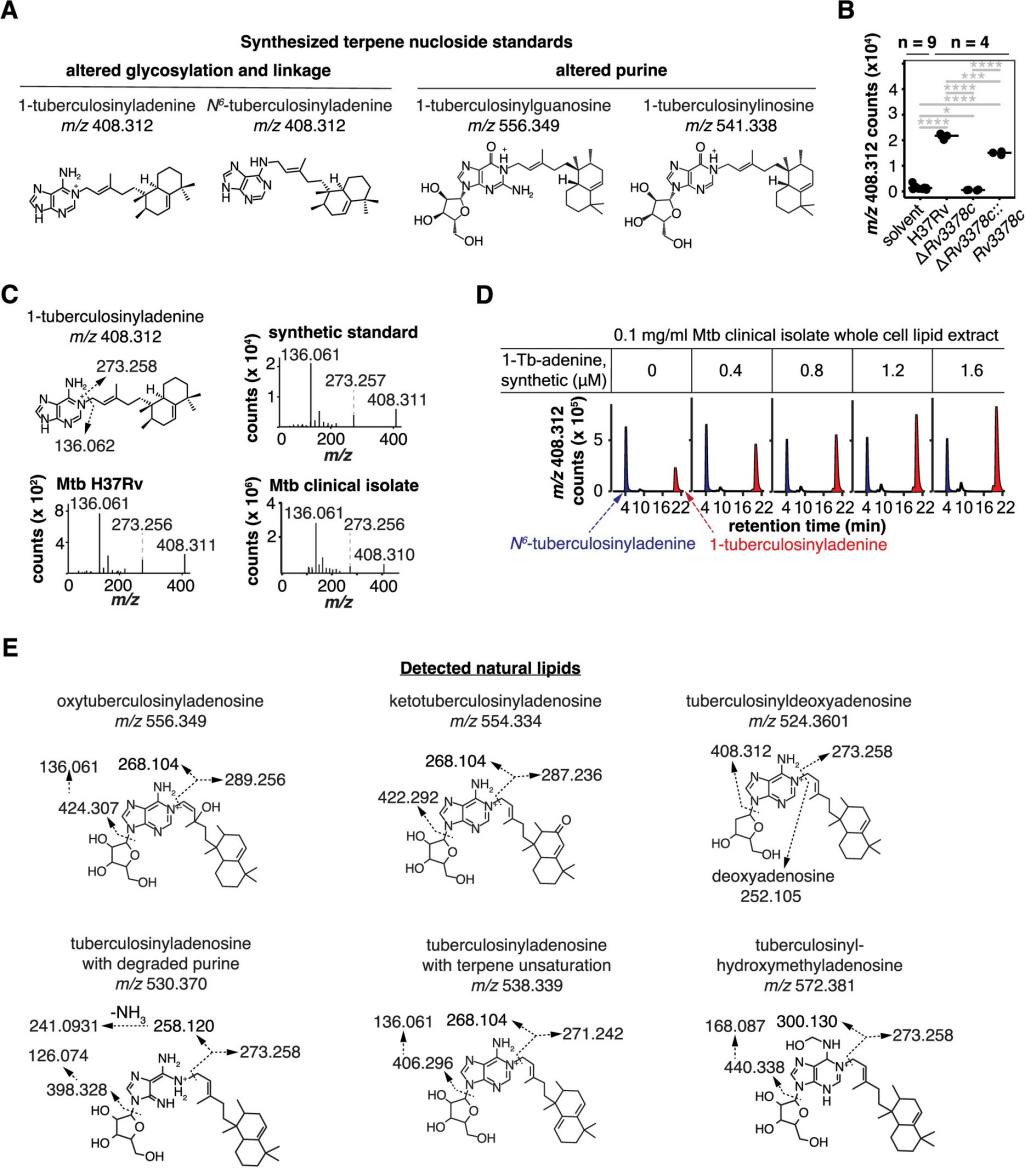
Unknown *Rv3377-3378c*-dependent lipids were identified as new terpene nucleoside family members. (A) Structures of synthetic molecules used to analyze natural compounds. (B) Intensities of ion chromatograms corresponding to *m/z* 408.312, the most abundant non-TbAd lipid found in the differential abundance analysis. Significant pairwise *t* tests after Benjamini–Hochberg adjustment are indicated (*: *p* < 0.05, **: *p* < 0.01, ***: *p* < 0.001, ****: *p* < 0.0001). Raw data for these measurements were provided in S1 Data. (C) CID-MS of *m/z* 408.312 showed fragmentation patterns diagnostic of 1-tuberculosinyladenine. The chemical structure with fragmentation shows calculated masses while spectra show observed masses. (D) Mtb clinical isolate M0014870-1 total lipid extracts were spiked with synthetic 1-tuberculosinyladenine, which showed co-elution with natural 1-tuberculosinyladenine and established its chemical identity and absolute yield in Mtb. (E) Annotated fragments from CID-MS established structures of 6 previously unknown terpene nucleosides where the calculated masses are shown. Collision localized modifications to the ribose, adenosine, or terpene but linkage within the moiety was inferred based on known analogous compounds.

We implemented CID-MS for direct detection of chemical fragments as a proof-of-principal for terpene nucleoside variants. Given lack of standards for terpene purines and terpene nucleosides, we adapted recent methods [10] to synthesize 4 key molecules, 1- and N^6^-tuberculosinyladenine, 1-tuberculosinylguanosine, and 1-tuberculosinylinosine (Figs 3A and S4 and S4 Data) that matched the putative structure of the most abundant variants. The latter 2 molecules differed from TbAd in purine usage, resulting in mass shifts of 15.995 and 0.984 amu, respectively. These standards tested alternate purine incorporation versus alternate changes like substitution of tuberculosinol for tuberculosinyl moieties (also 15.995 amu) in natural molecules.

We first focused on putative tuberculosinyladenine (*m/z* 408.312) because the ion had the highest intensity of any putative TbAd-derivative (S3 Data). Reanalysis of the lipidomics data specifically confirmed the loss of 1-tuberculosinyladenine with *Rv3378c* deletion and restoration with complementation (Fig 3B). CID-MS spectrum from a laboratory strain and a fresh clinical Mtb isolate had equivalent spectra, generating diagnostic fragments for adenine (*m/z* 136.061) and the tuberculosinyl lipid (*m/z* 273.258). Further synthetic 1-tuberculosinyladenine co-migrated with the endogenous lipid in a clinical isolate on HPLC-MS, formally ruling in the structural identification (Figs 3D and S5 and S4 Data) [30]. The method of standard additions confirmed that 1-tuberculosinyladenine was extremely abundant despite being previously unknown, accounting for approximately 0.2% of total lipid in H37Rv and patient-derived strains (S5 Fig).

Synthetic tuberculosinylguanosine and tuberculosinylinosine (Fig 3A) failed to co-elute with the Mtb lipids of matching mass (S6 Fig). Ruling out tuberculosinylguanosine helped to identify an endogenous lipid that differs from 1-TbAd by 16 amu as oxytuberculosinyladenosine (Fig 3E) using CID-MS. Likewise, ruling out tuberculosinylinosine suggested that *m/z* 541.339 was most likely the first isotope of TbAd.

We next targeted the other 11 putative TbAd modifications by CID-MS aiming to identify fragments consistent with the proposed modifications. CID-MS ruled in keto-tuberculosinyladenosine, the deoxyribose variant tuberculosinyldeoxyadenosine, an unsaturation in the terpene, a hydroxymethyladenosine, and a TbAd-like molecule with a degraded purine (Fig 3E). Hence, changes to the ribose, adenine and terpene components of TbAd were validated. CID-MS established the altered moiety, but putative structures (Fig 3E) were assigned linkages based on similarity to other known molecules [31].

**Fig 4:**
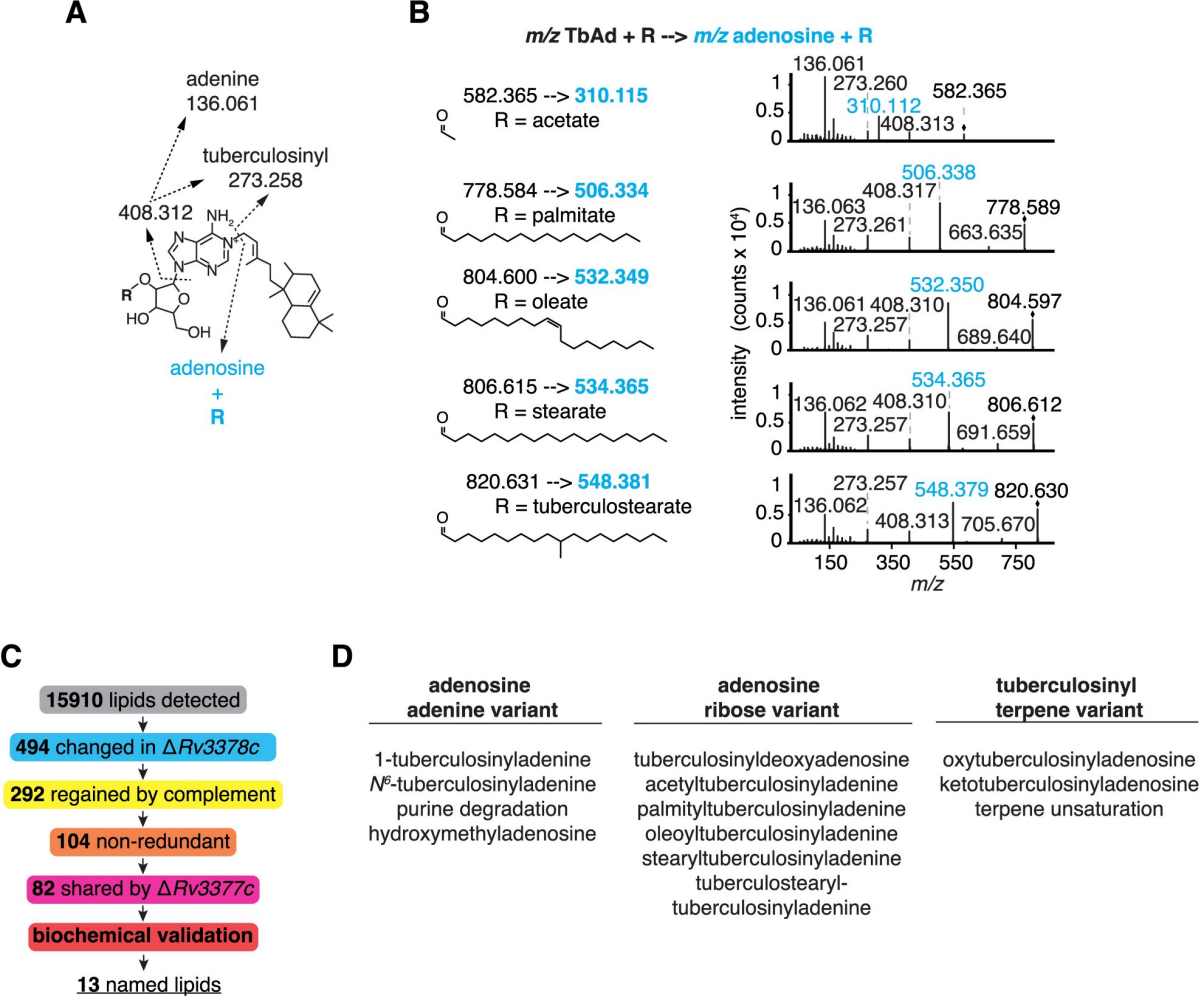
Fragmentation and identification of adenosine-containing lipids revealed terpene nucleoside family members. (A) Annotated fragments and calculated masses of lipid-linked 1-TbAd derivatives detected by CID-MS. The R group was assigned as ribose-linked based on ribose-fatty acyl fragments with the 2-linkage favored based on its chemical reactivity and known structures; however, linkage position could not be assigned directly from MS. (B) CID-MS showed diverse fragmentation patterns containing adenosine and consistent with parent terpene nucleosides containing lipid-conjugated ribose. Observed masses are shown. (C) Schematic shows the unsupervised lipidomic discovery process for the new TbAd-like lipids. (D) Thirteen lipids were identified as *Rv3378c* dependent and validated through CID-MS and synthetic chemistry.

CID-MS of TbAd reliably yields a characteristic adenine fragment (*m/z* 136.061) that is rarely present in other lipids, so we reasoned that unknown TbAd-like molecules might also yield adenine fragments (Fig 4A, blue). Using whole-cell lipid extracts of Mtb H37Rv as starting material, we searched for parental ions that released adenine (*m/z* 136.061) by targeting all lipids based on abundance after excluding previously collided masses (Fig 4A). Five distinct parent ions with a mass greater than 1-TbAd yielded, adenine, tuberculosinyl (*m/z* 273.258), and tuberculosinyladenine (*m/z* 408.312) fragments. By subtracting the mass of TbAd, we could deduce the presence of ribose-linked fatty acids (Fig 4A) consistent with the observed CID-MS fragmentation patterns (Fig 4B). This new approach independently identified acetyl- and oleoyl-TbAd structures seen also in the first lipidomics approach (S3 Data). Further we detected palmitoyl-, stearyl-, and tuberculostearyl-TbAd.

Automated metabolite peak identification and differential abundance analysis favored detection of metabolites with chromatographically distinct peaks and high relative abundance. Hence, genuine low abundance lipids of interest like tuberculostearyl-TbAd, detectable by the adenine fragment approach, were likely filtered out by the stringency of the lipidomics pipeline. Pooling the CID-MS spectra of *limms*-identified and adenine-containing lipids, we used network visualization to analyze precursor masses and fragments. This combined approach showed similarities and differences between TbAd variants of adenine, the adenosine ribose, and terpene moieties (S7 Fig). Furthermore, fragments consistent with the modifications proposed in Fig 2B for *m/z* 558.365 and 570.365 were identified.

Overall, despite decades of intense study of this major pathogen, the detection of many new molecules pointed to both the unsolved nature of the Mtb lipidome and the need for new tools that link chemical signatures to genes. *limms* software and the fragmentation methods identified a chemically diverse family of terpene nucleosides from a comparative lipidomics surveys of >20,000 events, leading to dozens of nonredundant, complemented candidates downstream of *Rv3378c* (Fig 4C) and positive identification of 13 named new molecules (Fig 4D). We sought to characterize patterns of diversity in the TbAd family, but these fully and partially solved compounds still likely underestimate the actual diversity of terpene nucleosides in Mtb.

### Terpene nucleosides in the MTC and adjacent species

**Fig 5:**
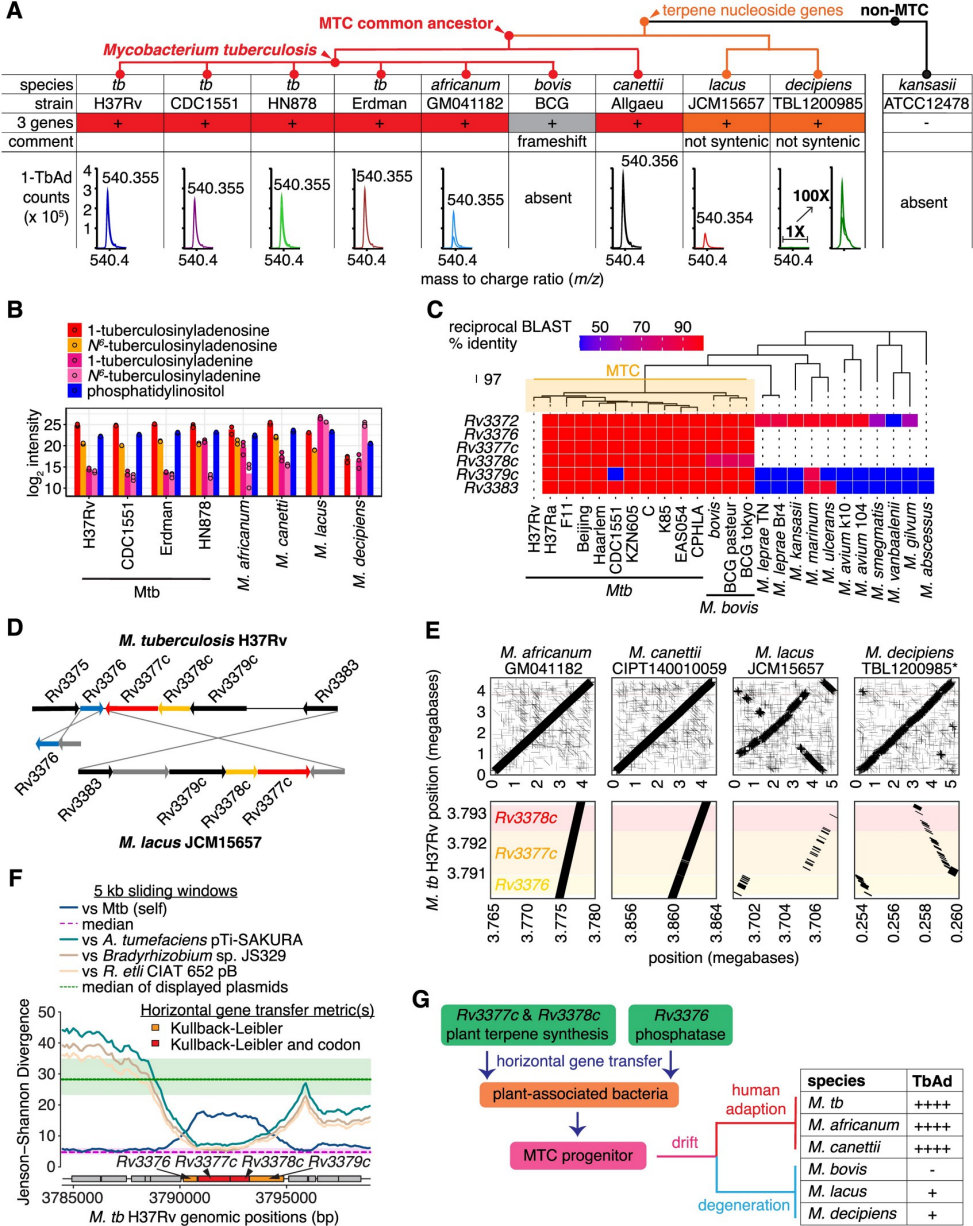
The timing and origin of horizontal transfer of biosynthetic genes for terpene nucleosides. (A) Mass spectrometry tested 1-TbAd production and abundance in MTC species that contained the three-gene locus. Positive mode extracted ion chromatograms for 1-TbAd show lipid counts near *m/z* 540.354 (observed mass shown) at a retention time of approximately 23 min in samples at 1 mg/ml total lipid. The lack of 1-TbAd in *M*. *bovis* and M. *kansasii* was documented previously [5,12]. A cladogram of arbitrary branch lengths reflected plausible organization [33]. (B) Peak intensity of a predominant membrane lipid, phosphatidylinositol, along with 1- and *N*^*6*^-TbAd, and 1- and *N*^*6*^-tuberculosinyladenine were measured among a panel of 8 MTC strains and species. (C) Reciprocal BLAST hit scores versus H37Rv are shown as a heatmap for the terpene nucleoside biosynthetic genes *Rv3376*, *Rv3377c*, and *Rv3378c* along with flanking genes. The neighbor-joining species dendrogram was based on whole-genome presence/absence of orthogroups. (D) Locus organization in *M*. *lacus* is rearranged relative to Mtb H37Rv. (E) Synteny of *Mycobacterium* species is shown for the whole genome and *Rv3376-8c* locus as dot plots compared to the reference Mtb H37Rv genome. The *M*. *decipiens* TBL1200985 genome is not fully assembled; hence, genome positions were inferred by scaffolding using the Mtb H37Rv genome. (F) Jenson–Shannon divergence profiles of Mtb H37Rv comparing to Mtb H37Rv genome itself or to DNA sequences from other bacteria using a 5 kb sliding window with 100 bp step. The gene schematic is colored according to Kullback–Leibler divergence. (G) Schematic of gene acquisition shows divergence and function. The data for Fig 5B and 5C can be found in S1 Data.

The TbAd biosynthesis genes are restricted to *Mycobacteria* [6–8], but whether the genes are functional had not been evaluated. Since infection competence for mammals is a hallmark of species in the MTC, with only Mtb and *M*. *africanum* further evolving into human lung specialists, we sought to correlate terpene nucleoside genotypes, host tropism, and TbAd biosynthesis using representative *Rv3378c*-containing pre-MTC and MTC strains. Common laboratory Mtb strains H37Rv, CDC1551, HN878, and Erdman all provided strong signals of similar absolute intensity, but other MTC species varied in 1-TbAd levels, with *M*. *africanum* and *M*. *canettii* [8] showing strong 1-TbAd signals equivalent to Mtb (Fig 5A). The non-MTC ancestor *M*. *kansasii* [5,12] was previously documented to lack 1-TbAd production, while the MTC strain *M*. *bovis* BCG [5,8] was shown to harbor an inactivating frameshift mutation in *Rv3377c* [32].

Unexpectedly, our genomic analysis identified TbAd loci in 2 clinical isolates of 2 recently identified non-MTC species, *M*. *lacus* and *M*. *decipiens* [34,35], adjusting the known timing of horizontal gene transfer to a point after *M*. *kansasii* divergence but before the MTC radiation (Fig 5A and S2 Table) [36]. Despite having all 3 biosynthetic genes, *M*. *lacus* and *M*. *decipiens* chemotyping detected 4- to 100-fold lower 1-TbAd signals relative to Mtb (Fig 5A). Whereas 1-tuberculosinyladenine and 1-TbAd had similar relative profiles across strains and species, *M*. *lacus* and *M*. *decipiens* accumulated 1-tuberculosinyladenine at higher concentrations than 1-TbAd or any other species of the MTC (Fig 5B).

Integrating genome sequence with TbAd chemotyping analysis revealed which gene variants in *Rv3376*, *Rv3377c*, or *Rv3378c* were functional (Fig 5A and S2 Table). A total of 197 genomes from 10 MTC species revealed species-restricted coding variants (S2 Table). These included an *Rv3377c* frameshift mutation previously suggested to block 1-TbAd production in *M*. *bovis BCG* [5], which was observed across 129 of 130 *M*. *bovis* strains and all *M*. *caprae* strains, suggesting both these animal-adapted pathogens lost TbAd production due to this lesion. In contrast, the *Rv3377c* glycine to valine mutation in 29 of 34 *M*. *africanum* strains did not alter 1-TbAd production (Fig 5A and S2 Table). Likewise, *M*. *canettii* Allgaeu showed production even with approximately 1% sequence divergence (Fig 5A and S2 Table).

Extending prior work [5–8] beyond the MTC, *Rv3376*, *Rv3377c*, and *Rv3378c* orthologs were either present or absent *en bloc*, suggesting a single transfer event involving all 3 genes (Fig 5C). The human-adapted species that cause pulmonary TB, Mtb and *M*. *africanum*, the emerging human pathogen *M*. *canettii* [31], the animal-adapted species *M*. *bovis*, *M*. *caprae*, *M*. *microti*, *M*. *mungi*, *M*. *orygis*, and *M*. *pinnipedii* had all 3 genes in tandem (S2 Table). We did not chemotype these species but their sequences and the trends in Fig 5A are consistent with terpene nucleoside production for all except *M*. *bovis* and *M*. *caprae*, which have an *Rv3377c* inactivating frameshift [32]. Whereas the locus in all MTC species had high synteny, *M*. *lacus* [34] and *M*. *decipiens* [35] showed substantive genomic rearrangements and altered gene order (Fig 5D and 5E) along with markedly lower biosynthesis, suggesting horizontal transfer of a functional locus followed by degeneration through rearrangement.

The locus was absent in all other non-MTC species surveyed, including *M*. *marinum*, *M*. *avium*, the skin pathogen *M*. *leprae*, the commensal *M*. *smegmatis*, and the proposed common ancestor of the MTC, *M*. *kansasii* [12,17]. Hence, genome analysis indicated locus acquisition in a single event by an MTC progenitor near to the evolutionary time that parasitism of mammals began to evolve at the outset of MTC complex radiation. Frameshift mutations (*M*. *bovis*, *M*. *caprae*) and locus degeneration (*M*. *lacus*, *M*. *decipiens*) subsequently occurred in some species (Fig 5G).

### Origin on a plasmid from plant-associated bacteria

Pethe and colleagues noted the atypical G-C content of *Rv3377c* and *Rv3378c* was consistent with horizontal gene transfer [6], a finding Becq and colleagues extended to include *Agrobacterium* or *Rhizobium* as possible donors [37]. When our initial efforts to find orthologs of *Rv3376*, *Rv3377c*, or *Rv3378c* outside of *Mycobacteria* failed, we searched for a genetic donor that more closely matched TbAd biosynthesis gene nucleotide composition among all available DNA sequences (Fig 5F). The 1-TbAd locus most closely resembled sequences from *Agrobacterium* and *Rhizobium* (Fig 5F) in agreement with Becq and colleagues; however, we identified the sequences as plasmids from plant-associated bacteria. A short stretch of the *Bradyrhizobium* chromosome was also identified (Fig 5F) but the rest of the genome was not implicated, more consistent with a sequence transferred to both *Bradyrhizobium* and *M*. *tuberculosis*.

While the ancestral genes were not identified directly, *Agrobacterium tumefaciens* tumor-inducing (Ti) plasmids like the one identified here encode specialized machinery for horizontal gene transfer [38], providing a plausible mechanism for punctuated evolution. We speculate the terpene biosynthesis genes *Rv3377c* and *Rv3378c* were collated with the hydrolase *Rv3376*, which was less diverged from the Mtb genome and is oppositely transcribed, prior to a single horizontal gene transfer into the common ancestor of the MTC, *M*. *lacus*, and *M*. *decipiens* (Fig 5G). Genetic changes subsequently tuned 1-TbAd production while MTC species evolved distinct host tropisms, with the species that cause human TB epidemics showing high-level constitutive 1-TbAd biosynthesis.

### Regulation of 1-TbAd biosynthesis genes in Mtb

The proposed horizontal gene transfer from plant-associated bacteria suggested terpene biosynthesis gene regulation operated across genera. Conversely, consuming the essential metabolites geranylgeranyl pyrophosphate and adenosine as substrates might impose a fitness cost if no regulation to offset the biochemical expense is present. This premise, along with recent studies suggesting that 1-TbAd is produced at very high levels but does not affect in vitro growth in the absence of stress, suggested that 1-TbAd locus might be subject to gene regulatory control. However, evaluating Mtb transcriptional data assembled by Yoo [39] from 647 samples spanning 231 unique conditions found no examples of 1-TbAd biosynthesis genes altered by more than 4-fold relative to log-phase growth in media. Even stimuli expected to induce strong repression such as stationary phase growth, hypoxia, and altered carbon source [40] showed only mild change (S8A Fig).

**Fig 6:**
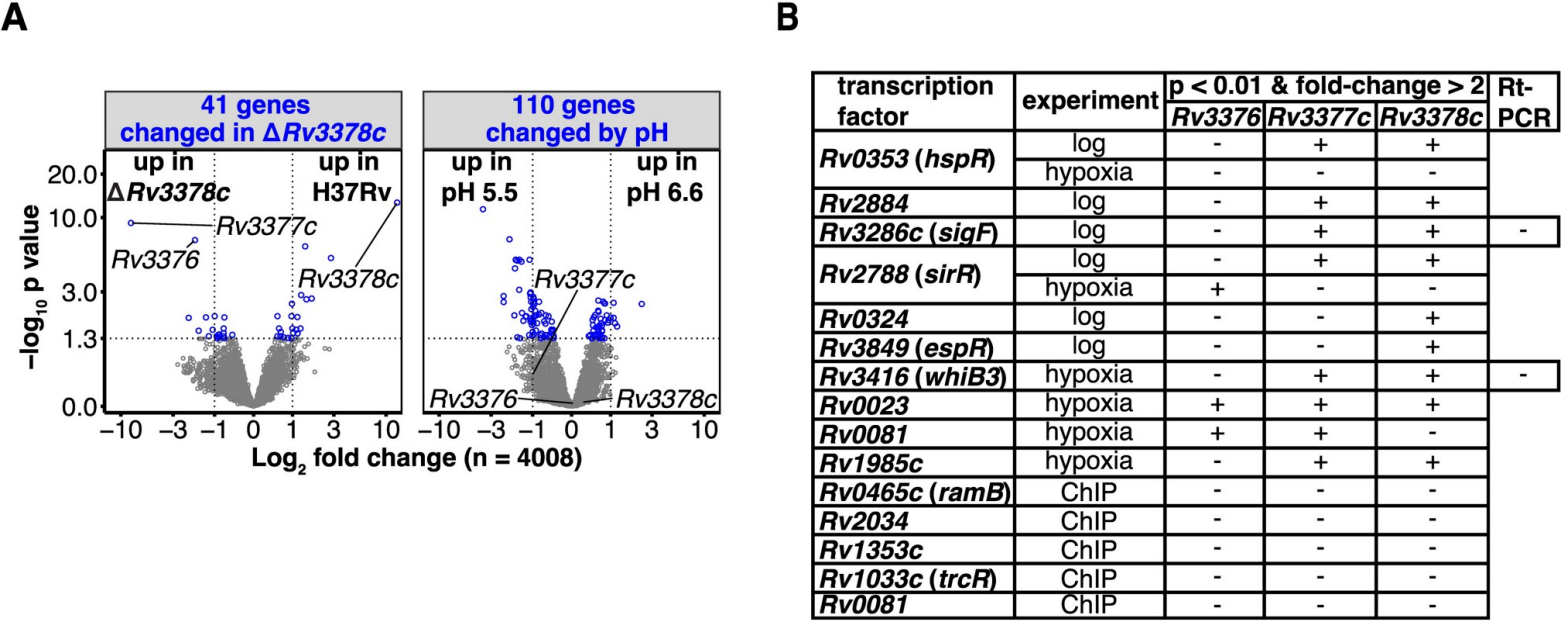
Terpene nucleoside gene regulation. (A) Gene expression measured by RNAseq was compared in the *Rv3378c* mutant and the parental strain at pH 6.6 or 5.5. A nested contrast of the *Rv3378c* mutant versus the parental strain at each pH indicated the genes with differential abundance with respect to the *Rv3378c* mutant (left), while a nested contrast of pH 5.5 versus 6.6 shared by both strains showed pH responsive genes (right). Differentially abundant genes (blue; Benjamini–Hochberg adjusted *p* value <0.05) and the TbAd biosynthesis pathway genes are indicated. The underlying transcriptomics data can be found in S1 Data. (B) Transcription factor overexpression strains that caused significant alterations in *Rv3376*, *Rv3377c*, or *Rv3378c* transcripts in log-phase growth or during the induction and release of hypoxia are shown. Transcription factors physically associated with *Rv3376*, *Rv3377c*, or *Rv3378c*, measured by ChIP-seq, were included.

We next looked for a transcriptional signature of compensatory regulation by comparing the transcriptomes of mutants lacking 1-TbAd, reasoning that loss of an abundant lipid family synthesized from essential substrates might cause feedback regulation elsewhere in the genome. Transcripts for the downstream genes *Rv3376* and *Rv3377c* increased after *Rv3378c* deletion (Figs 6A, S8B and S8C), suggestive of a feedback loop. Otherwise, among the 4,008 genes measured, only 41 had significant transcriptional changes relative to the Mtb H37Rv parent, less than number of genome-wide false positive associations expected by chance. Further, changed transcripts did not cluster into recognized regulatory pathways by gene-set enrichment analysis.

Since 1-TbAd affects lysosomal acidification [6,10,11,41], we next tested acid pH as a transcriptional stimulus. Shifting cultures from pH 6.6 to pH 5.5 induced more substantial transcriptional reprogramming than gene deletions alone (Figs 6A and S9A), but transcription of the TbAd biosynthesis locus was not altered. Further, the pH signature shifted equivalently in H37Rv and TbAd biosynthesis mutants (S9A Fig). Thus, the known effect of 1-TbAd to confer better survival at acid pH (6, 8, 9) is likely constitutive rather than induced.

*Rv3377c* was shown to require a magnesium cofactor and be inhibited by high magnesium concentrations [32,42]. Hence, we assayed 1-TbAd production in *Mycobacterium tuberculosis* grown in 3 concentrations of magnesium (0.6, 6.0, and 60 mM) reported to alter magnesium homeostasis without inhibiting growth [43]. We did not measure significant (Benjamini–Hochberg adjusted *p* value of the F-test) changes in the levels of 1- or *N*^*6*^-TbAd (S9B–S9E Fig). This outcome did not rule out the potential for biochemical activation via optimization of Rv3377c activity for the low magnesium levels observed upon phagosomal engulfment as previously indicated [32], but was consistent with constitutive TbAd production when magnesium was replete.

To broadly investigate gene regulatory potential, we used data from a library of transcription factor induction constructs [11] to survey *trans*-acting proteins. The genome-wide effects of 183 of 206 known Mtb transcription factor [44] and serine/threonine protein kinase (STPK) knockout or induction strains [45] were surveyed for altered expression of any 1-TbAd biosynthesis gene. Only 6 transcription factors altered transcription more than 2-fold during log-phase growth, with 5 reducing expression and only *Rv3286c* (*sigF*) increasing expression. No transcription factor altered expression of all 3 genes simultaneously, and any change was less than 4-fold (Fig 6B). Attempted validation of the 2 transcription factors with the largest differences (*sigF* and *whiB3*) by real-time quantitative PCR showed that only *Rv3377c* was significantly altered, only by *sigF*, and by a modest decrease of less than 4-fold (S10 Fig). Thus, both genome-wide and targeted transcriptional and transcription factor analysis revealed a constitutively transcribed locus insulated from strong transcriptional regulation. The lack of strong, detectable gene regulation is contrary to the conditional regulation hypothesis, but it is consistent with horizontal gene transfer and self-regulated gene expression. Consistent expression and abundant production are important features of pathogen-shed metabolite biomarkers of infection.

## Discussion

The transmission and sequelae of tuberculosis disease are confoundingly variable [46]. The array of unique Mtb lipids coordinated by a genome with only 60% functional annotation is also challenging. Even when annotated, many biosynthetic enzymes are both promiscuous and act in nonlinear pathways, yielding multiple small molecule products and forming networks between seemingly unconnected cellular processes. Thus, the biosynthetic genes and disease-determining metabolites of even the world’s most deadly bacterium remain substantially unannotated. Nonetheless, shed molecules like 1-TbAd reprogram host cells [10,11,41] to shape pathogenesis, despite being encoded by enzymes rather than acting as effector proteins that fit the molecular arms race canon.

To facilitate moving between genotypes and broadly measured phenotypes for these metabolic effectors, we provided new profiling and software-based bioinformatic tools. Here, we used these tools to detect and analyze lipids dependent on the Rv3378c enzyme, which revealed insights into the origin and natural history of 1-TbAd in mycobacteria and the discovery of many unexpected downstream metabolites. High level terpene nucleoside biosynthesis is, with rare exceptions, restricted to pathogens that broadly disseminate and cause TB in humans, so all of the 292 complemented *m/z* values represent potential TB-specific biomarkers [24] that can be sensitively tracked with existing MS methods in serum [47] and breath [28].

We identified 13 previously unknown lipids with the chemical components of antacids and lysosomotropes. From the chemical biology perspective, the ready detection of multiple new lipids demonstrated the value of unsupervised bioinformatic approaches for discovery in metabolomic studies. Furthermore, the improvements to differential abundance analysis brought by *limms* meant that the lipids were attributable to genes and the chemical signatures to modular chemical units, insights hidden in lists of cellular lipids. While *limms* provided a new tool for discovery through differential abundance, we also developed an innovative wide mass window collision strategy and employed synthetic chemistry to validate in silico findings.

The newly identified terpene nucleosides represent new disease associated candidate biomarkers and point to future studies of new functions. For example, hypotheses inspired by the structures included the similarity of *N*^*6*^-tuberculosinyladenine to isoprene cytokinins that act as small molecule signals in Mtb [48]. In this study, we could not distinguish biosynthesis using alternative substrates versus chemical modification, such as oxidation, after 1-TbAd production. However, 2 oxygenated terpene variants suggest TbAd could double as an oxygen as well as proton sink. Finally, since TbAd can cross membranes to enter lysosomes [10], the 6 new lipid-linked lysosomotropes now become candidate transporters of lipids to lysosomes, a hypothesis that is independently supported by TbAd induction of lysosomal lipid overload [11].

Extending the expanded terpene nucleoside discoveries to the MTC through chemotyping, we observed a striking correlation of high 1-TbAd biosynthesis with mycobacterial virulence in humans, where only *M*. *canetti*, Mtb and *M*. *africanum* maintained high TbAd production. Inactivating single-nucleotide polymorphisms and locus degeneration were apparent in animal-adapted species, while 2 species that very rarely infect or transmit from humans favored tuberculosinyladenine over TbAd. *M*. *lacus* and *M*. *decipiens* were isolated from human infections of synovium and skin, respectively, but are rare and are not known to cause pulmonary disease in humans [34,35]. Although their natural host tropism is unknown, their spectrum of disease, including a recent study showing *M*. *decipiens* has a lower optimum growth temperature than Mtb [49], as well as overproduction of tuberculosinyladenine are intriguing components for future studies.

For the MTC, horizontal gene transfer of *Rv3377c* and *Rv3378c* into a common ancestor likely resulted in an abrupt, immediate metabolic shift, given that laboratory transformation of the non-MTC species *M*. *kansasii* with these 2 genes is sufficient for 1-TbAd production [12]. Here, we provided evidence for collation and transfer of these genes originating in plant-associated bacteria, as well as the timing of transfer prior to the MTC radiation. This punctuated evolutionary gene transfer event likely also underlies the transcriptional character of the locus we described: basally active, minimally regulated, and largely free of trans-acting factors.

A lack of recombination or gene acquisition events among tens of thousands of sequenced MTC genomes have led to the suggestion that Mtb was largely shaped by point mutations, insertions, and deletions [50]. However, horizontal gene transfer at the onset of MTC radiation, perhaps prior to an obligate parasitic lifestyle, contributed genes necessary for in vivo mammalian pathogenesis [16,51,52]. However, horizontal transfer can simultaneously bring interdependent genes to cause discrete gain of function related to previously nonexistent, complex pathways [56]. Here, we showed acquisition and non-loss of terpene nucleosides that disrupt lysosomal function [10] and diminish control of pathogen growth [6,11,41] was phylogenetically coincident with gain of the ability to survive inside and transmit among human hosts.

These data are consistent with the conclusion that 3 gene transfer was a key event in the evolution of MTC species into human pathogens. Beyond this central conclusion, the new terpene nucleosides are candidate biomarkers. Furthermore, prior studies showing *N*^*6*^- and 1-TbAd comprise 1% to 3% of cellular lipid [10] likely underestimated the total terpene nucleoside content, which needs to account for tuberculosinyladenine and other abundant family members. This discovery and increased contribution could make a critical difference for chemical titration of macrophage lysosomes and detection of virulence-related molecules for TB diagnosis [10].

## Materials and methods

### Culture and extraction of Mtb strains and species

Growth of microbial strains to late log phase in 7H9 media was as described [4]. Analysis of the MTC used the following strains: Mtb H37Rv, Mtb CDC1551, Mtb HN878, Mtb Erdman, *M*. *africanum* GM041182; *M*. *bovis* BCG Paris, *M*. *canettii* Allgaeu: recently discovered non-MTC strains were *M*. *lacus* JCM15657 and *M*. *decipiens* TBL1200985. To test the effect of magnesium, Mtb H37Rv was grown in Mg-GAST medium supplemented with 0.6, 6.0, or 60 mM magnesium chloride [43] for 4 weeks, yielding similar OD600 of 0.5. Growth defects were not observed.

### Mass spectrometry

Whole-cell extraction and mass spectrometry optimized for Mtb lipids was as described [4] and used chloroform:methanol, chromatographic separation on a normal-phase Inerstil Diol column (GL Sciences, Tokyo, Japan), and an Agilent 6520 Accurate-Mass QToF with 1200 series HPLC. Directed CID-MS targeted candidate lipids with voltages between 20 and 35 mV. To generate adenine containing fragments without target selection, data-dependent acquisition using a 1 amu window and variable collision energy (E = (*m/z*)/100 + 20) was used selecting a maximum of 2 precursor ions > 260 amu per cycle. A counts threshold and an exclusion list generated from previously collided ions was used, with solvent blanks and extracted media preceding 5 samples of Mtb H37Rv.

### Computational methods and genome analyses

Statistical testing not using *limms* used base R, except for S5 Fig that used GraphPad Prism. Detailed analyses and code for statistical comparisons, transcriptomics analysis, synteny analysis, and data visualizations are provided in S1 Data. For the analyses of mass spectrometry data using *t* tests, zero values were replaced with ones to allow calculation of non-infinite values prior to testing, then *t* tests and *p* value adjustment by the Benjamini–Hochberg method were used. Within R, synteny analysis used the R package DECIPHER [53]. Network visualization used igraph [54] and ggnetwork [55] on spectra analyzed using MassHunter (Agilent) to select fragments diagnostic of TbAd; observed precursor and fragment masses are provided in S1 Data.

Genome sequences for all strains and species were obtained from Genbank [56], and included: Mtb H37Rv (GCA_000195955.2), *Mycobacterium africanum* (GCA_000253355.1), *Mycobacterium orygis* (GCA_015265495.1), *Mycobacterium caprae* (GCA_001941665.1), *Mycobacterium microti* (GCA_001544815.1), *Mycobacterium pinnipedii* (GCA_002982275.1), *Mycobacterium bovis* (GCA_005156105.1), *Mycobacterium bovis* pasteur (GCA_025908415.1), *Mycobacterium mungi* (GCA_001652545.1), *Mycobacterium canettii* (GCA_000253375.1), *Mycobacterium lacus* (GCA_010731535.1), *Mycobacterium decipiens* (GCA_002104675.1), *Mycobacterium kansasii* (GCA_000157895.2), and *Mycobacterium marinum* (GCA_016745295.1). Whole genome alignments were performed using MAUVE [57] to establish genome coordinates relative to Mtb H37Rv, including the locus rearrangements in *M*. *lacus* JCM15657 and *M*. *decipiens* TBL1200985. Gene variants in the terpene nucleotide locus were identified and compiled using BLAST [58] on all whole genome sequences for the analyzed species that were available on Genbank on 05/21/2021. The presence/absence BLAST score matrix was compiled using genewise BLAST [58] searches of the specified genomes against Mtb H37Rv. Phylogeny in the neighbor-joining tree was based on whole genome presence or absence of orthogroups. Analysis of horizontal gene transfer was as described [16]. Sequences matching the terpene nucleoside locus were: *Agrobacterium tumefaciens* Ti plasmid pTiBo542 (GI:190014640), *Agrobacterium tumefaciens* pTi (GI:10955016), *Agrobacterium tumefaciens* MAFF301001 pTi-SAKURA (GI:10954820), *Bradyrhizobium* sp. JS329 (GI:335999372), *Rhizobium etli* CIAT 652 pB (GI:190893983).

### *Rv3376*, *Rv3377c*, and *Rv3378c* genetic manipulations

Mtb strain H37Rv was used for in frame deletion of *Rv3376*, *Rv3377c*, *Rv3378c*, and *Rv3377c-Rv3378c* by gene replacement [59]. A targeting construct with 500 bp flanking regions of the gene and loxP-hygromycin-LoxP cassette was cloned into a pUC57 vector. Linear targeting DNA was amplified from the vector and transformed into H37Rv carrying the pNit-recET-SacB-kan plasmid (1) expressing recombinase. Transformed bacteria were selected on 7H10 agar plates containing 50 μg/ml hygromycin. Recombinants were further selected by growth on 7H10 plates containing 5% sucrose and hygromycin (50 μg/ml), then were subsequently screened on 7H10 plates with kanamycin (25 μg/ml). Colonies were screened for in frame deletion of the target gene and presence of hygromycin cassette by PCR and sequencing. The primers used were: P1(gctgcggtggaatatcagac), P2 (gaattcatccgatcaagcaagg), P3 (gtttgtgggatctggcgc), P4 (cattggaggagatcgaacgc), P5 (atatcgtacaggcgctcgaa). Complementation was performed by integrating a single copy of the gene under the control of the constitutively expressing MOP promoter in the pJEB402 vector [60], confirmed by measuring 1-tuberculosinyladenosine using HPLC-MS.

### limms

The open source *limms* software package was written in R and includes functions for preprocessing and normalizing a peak intensity table, statistical inference of differentially abundant compounds, and annotation of features. The *limms* function imputeZerosUnifMin replaced zeros with random local minima to mimic the threshold of detection and avoid distorting variance calculations with the abundant zero measurements in mass spectrometry data. The function runNorm log2 transformed and normalized data via full quantile normalization. The function limmaTest, a simplified interface for the *limma* functions lmfit, eBayes, and topTable, was used to build a linear model including Bayesian smoothing of variance and generate tables of summary statistics [18,20]. The function dbMatch identified matches to a user-suppled database using specified mass and retention time variance windows.

In addition to the R package, which includes help pages for each function, a package vignette also provided as S2 Data contains explanations of *limms* functions in detail and step-by-step instructions for their use. The help pages and S2 Data contain an instructional narrative and annotated code for re-analysis of published data [23]. *limms* and the vignette are available at https://github.com/jamayfie/limms.

In addition to the analysis provided in S2 Data, the R Markdown provided in S1 Data includes all code for the complete Mtb lipidomic analyses in the manuscript. When run with the included source files, S1 Data regenerates the data analyses, outputs including S3 Data, and visualizations used to produce the manuscript figures. The R package XCMS [22,61] was used to identify mass spectrometry peaks and align them across samples to generate the grouped peak/intensity tables used for S1, S2 and S4 Data.

### Synthetic chemistry

The synthesis of tuberculosinyladenine, tuberculosinylinosine, and tuberculosinylguanosine is described in S4 Data. Quantification of 1-tuberculosinyl adenine and 1-TbAd. Mtb total lipids (0.1 mg/ml) were spiked with a known concentrations of synthetic 1-tuberculosinyladenosine or synthetic 1-tuberculosinyladenine and analyzed by normal phase HPLC-MS. The chromatogram peak areas of *m/z* 408.312 and *m/z* 540.354 were extracted for 1-tuberculosinyladenine and 1-tuberculosinyladenosine, respectively, and plotted against the spiked concentrations of synthetic compounds.

### Transcriptomics

Approximately 0.5 to 1 μg of total RNA at 50 to 100 ng/μl was prepared for RNA-seq using the Ribo-Zero Magnetic Bacterial kit (Epicentre) in connection with TruSeq Stranded Total RNA kit (Illumina), and 10–20 × 106 50 bp paired-end reads were obtained for each sample replicate on an Illumina HiSeq 2500. Demultiplexing and adapter removal were performed using fastqc [62], followed by alignment to the Mtb H37Rv genome (NC_000962.3) using bwa-mem [63]. Bam files were sorted and merged using samtools [64] and gene counts were obtained using featureCounts from the Rsubread package [65]. Differential abundance analysis of RNAseq data used edgeR [66] and limma [18, 20] with code in S1 Data; raw data are available via BioProject accession PRJNA1146031.

### Transcription factor overexpression

Published transcription factor overexpression data [44,45] were re-analyzed for *Rv3376*, *Rv3377c*, and *Rv3378c* to identify transcription factors that induced a significant change in terpene nucleoside gene expression relative to baseline expression of all input microarrays [44].

## Supporting information

S1 Fig Deletion of *Rv3376*, *Rv3377c*, and *Rv3377c*-*Rv3378c*. Schematics of the gene replacement are shown for *Rv3376* (A), *Rv3377c* (C), and *Rv3377c*-*Rv3378c* (E). Validation by PCR amplification of the gene locus used primer sets flanking the target genes *Rv3376* (B), *Rv3377c* (D), and *Rv3377c*-*Rv3378c* (F). Corresponding primer sets are indicated as P1-P2 for *Rv3376*, P4-P5 for *Rv3377c*, and P4-P3 for *Rv3377c*-*Rv3378c* double mutant. (G) Growth curves of the Δ*Rv3378c*, Δ*Rv3378c*::*Rv3378c*, and Mtb H37Rv parent strains grown in 7H9 medium are shown. Uncropped gels for S1 Fig BDF and raw data for growth curves are provided in S1 Data and S1 Raw Images.
(PDF)

S2 Fig Ion chromatograms of Mtb H37Rv or engineered mutant strains with inset boxes showing select dithered peaks to distinguish the overlapping biological replicates. (A) 1- and *N*^6^-TbAd (*m/z* 540.354) are shown in 3 experiments comparing Mtb H37Rv (black), Δ*Rv3378c* (red), and the complemented strain Δ*Rv3378c*::*Rv3378c* (blue) in a validation experiment (*n* = 4); or Mtb H37Rv, ΔRv3377c (orange; dashed), Δ*Rv3378c*, and ΔRv3377-8c (gold; dashed) two-gene deletion in an independent experiment (*n* = 4); or Mtb H37Rv and Δ*Rv3376* (green) in an analysis of *Rv3376* function (*n* = 3). (B) 1- and *N*^6^-tuberculosinyladenine (*m/z* 408.312) measured in the Mtb H37Rv (black), Δ*Rv3378c* (red), and the complemented strain Δ *Rv3378c*::*Rv3378c* (blue). 1-TbAd (A) and 1-tuberculosinyladenine (B) area under the curve were used for statistical testing and to generate Figs 1B and 3A, respectively.
(PDF)

S3 Fig Using limms to identify statistically significant changes in a complex comparison reveals metabolites with unexpected patterns of change. Volcano plots of metabolites altered by disruption of function using a surrogate genetic system expressing the human vitamin B6-dependent enzyme cystathionine beta-synthase (CBS) in *S*. *cerevisiae*. Contrasts of the CBS major allele (MA) grown with high (400 ng/ml) or low (1 ng/ml) vitamin B6, compared to the G307S mutation at high (400 ng/ml) or low (1 ng/ml) vitamin B6, or under methionine replete versus starvation conditions. Annotations based on isotopically labeled, pooled standards are shown and tracked across conditions that affected the CBS cofactor, enzyme function, or substrate availability. Data for S3 Fig are provided in S1 Data.
(PDF)

S4 Fig The synthesis of 1-tuberculosinyladenine, N6-tuberculosinyladenine, 1-tuberculosinylguanosine, and 1-tuberculosinylinosine. Compounds were characterized by NMR and mass spectrometry and used to identify natural isolates. The synthesis procedures and characterization are provided in S4 Data.
(PDF)

S5 Fig Standard addition of synthetic 1-TbAd and 1-tuberculosinyladenine to measure their abundance in clinical isolates of Mtb. (A) The quantities of natural molecules were estimated by plotting of chromatogram area against the known concentrations of each synthetic compound to obtain the extrapolated number on the x-axis. The arrows pointed to the concentration of the natural molecules. (B) The amount of 1-TbAd and 1-tuberculosinyladenine measured in 8 independent clinical isolates relative to total cellular lipid measured on a balance. The measurements underlying this figure can be found in S1 Data.
(PDF)

S6 Fig Determination of structural identity by co-elution of the synthetic compounds with the natural Tb molecules. (A–C) The unknown, *Rv3378c*-dependent masses consistent with tuberculosinylguanosine and tuberculosinylinosine do not match synthetic tuberculosinylguanosine and tuberculosinylinosine. (A) Chemical structure of 1-tuberculosinylguanosine and overlayed extracted ion chromatograms of whole cell lipids from Mtb H37Ra and synthetic 1-tuberculosinylguanosine measured in the positive mode. (B) Chemical structure of 1-tuberculosinylinosine and overlayed extracted ion chromatograms of whole cell lipids from Mtb H37Ra, synthetic 1-tuberculosinylinosine and synthetic 1-tuberculosinylinosine spiked into H37Ra extract, measured using positive (B) and negative (C) mode mass spectrometry.
(PDF)

S7 Fig Network visualization of terpene nucleosides and CID-MS fragments. Terpene nucleoside precursors and their fragment ions (observed *m/z*) after CID-MS were compared to 1-TbAd and the four diagnostic fragments characteristic of CID-MS (calculated *m/z*) in a pairwise analysis. Only fragments diagnostic of TbAd or modifications of those fragments are shown, with all precursors and fragments within 15 ppm of their calculated mass. Node shapes indicate the precursor and fragment ions; colors indicate adenine, adenosine, tuberculosinyl terpene or tuberculosinyladenine fragments or modifications of those moieties. Vertices connecting precursors and fragments show the presence of shared or unique fragments.
(PDF)

S8 Fig Conditional down-regulation of terpene nucleoside biosynthesis genes. (A) Meta-analysis of published transcriptomics data showed normalized expression of *Rv3376*, Rv3377c, or *Rv3378c* in glucose medium during log-phase (blue with blue dotted line to demarcate the reference condition) versus conditions found to repress transcription >4-fold among the 231 unique conditions [39,40]. (B) Heatmap of the most significantly changed genes in a contrast of Δ*Rv3378c* to Mtb H37Rv, with genes clustered by hierarchical clustering after transcriptiomics using RNAseq. (C) Expression of the terpene nucleoside locus genes using quantitative reverse transcription PCR of mRNA in Mtb H37Rv and in the Δ*Rv3378c* or complemented Δ*Rv3378c*::*Rv3378c* strains, normalized to 16S ribosomal RNA. All pairwise contrasts were tested but only significant *p* values are shown (*t* test with Bonferroni correction). The data for S8 Fig can be found in S1 Data; BioProject accession PRJNA1146031 contains raw RNAseq data.
(PDF)

S9 Fig pH and magnesium showed no or modest interactions with TbAd biosynthesis. (A) Heatmap of transcripts with *p* value < 0.01 in a contrast of pH 5.5 versus 6.6 in both the Mtb H37Rv and Δ*Rv3378c* strains measured by RNAseq. (B) Mass spectra of 1-TbAd, *m/z* 540.354, or *N*^*6*^-TbAd (C), *m/z* 540.354, from replicate cultures grown in media containing 0.6, 6.0-, or 60-mM magnesium chloride (*n* = 3 replicates) with observed masses shown. The color key for magnesium concentration was shared for (B–E). Both combined and dithered peaks (inset) are shown to allow visualization of peak correspondence and individual samples. (D) Quantification and statistical analysis of 1-TbAd or *N*^*6*^-TbAd (E) after lipidomics analysis using *limms* (Benjamini–Hochberg adjusted *p* value after Welch ANOVA). Raw data for S9 Fig ADE is provided in S1 Data. Raw RNAseq data is available in BioProject accession PRJNA1146031.
(PDF)

S10 Fig Transcription factor overexpression of *sigF* or *whiB3* showed little influence on TbAd biosynthesis gene expression. Quantitative PCR analysis of gene expression during transcription factor overexpression. Abundance of terpene nucleoside locus transcripts in strains with inducible overexpression of the transcription factor *sigF* or *whiB3*, with anyhydrotetracycline (ATC) induction compared to uninduced conditions. Only significant *p* values are shown (pairwise *t* test with Bonferroni correction). The data for this figure are included in S1 Data.
(PDF)

S1 Table *limms* output of the 10 most significantly changed metabolites following cystathionine beta-synthase disruption. Data and analyses found in S1 Table are provided in S1 Data.
(PDF)

S2 Table *Rv3376-8c* coding mutations in Mtb complex species. (PDF)

S1 Data Raw data and R code for computational analyses and manuscript figures. The R Markdown document contains annotated R code for computational analyses and generation of manuscript figures. The associated files in the zipped folder contain the data necessary for the analyses.
(ZIP)

S2 Data *limms* vignette. The *limms* package vignette provided in html format. The complete R Markdown used to produce the html document is part of the *limms* package available at https://github.com/jamayfie/limms.
(ZIP)

S3 Data The complete results of differential abundance analyses of 2 independent mass spectrometry data sets. Results are contained in spreadsheets of lipidomic data. The tab Positive mode experiment 1 contains the results of the genetic analysis with single and double mutants of *Rv3377c* and *Rv3378c*. The tab Positive mode experiment 2 contains the *Rv3378c* mutant and complementation strains.
(XLSX)

S4 Data Schemata and validation of the chemical synthesis of tuberculosinyladenine, tuberculosinylguanosine, and tuberculosinylinosine. (PDF)

S1 Raw Images Raw Images. (PDF)

## Abbreviations

Mtb: *Mycobacterium tuberculosis*
MTC: *Mycobacterium tuberculosis* complex
STPK: serine/threonine protein kinase
TB: tuberculosis
