# Supplementary figures and images for: Mycobacteria that cause tuberculosis have retained ancestrally acquired genes for the biosynthesis of chemically diverse terpene nucleosides
Source: PLoS Biol. 2024 Sep 30;22(9):e3002813. doi: 10.1371/journal.pbio.3002813 (PMC11476799; doi:10.1371/journal.pbio.3002813)

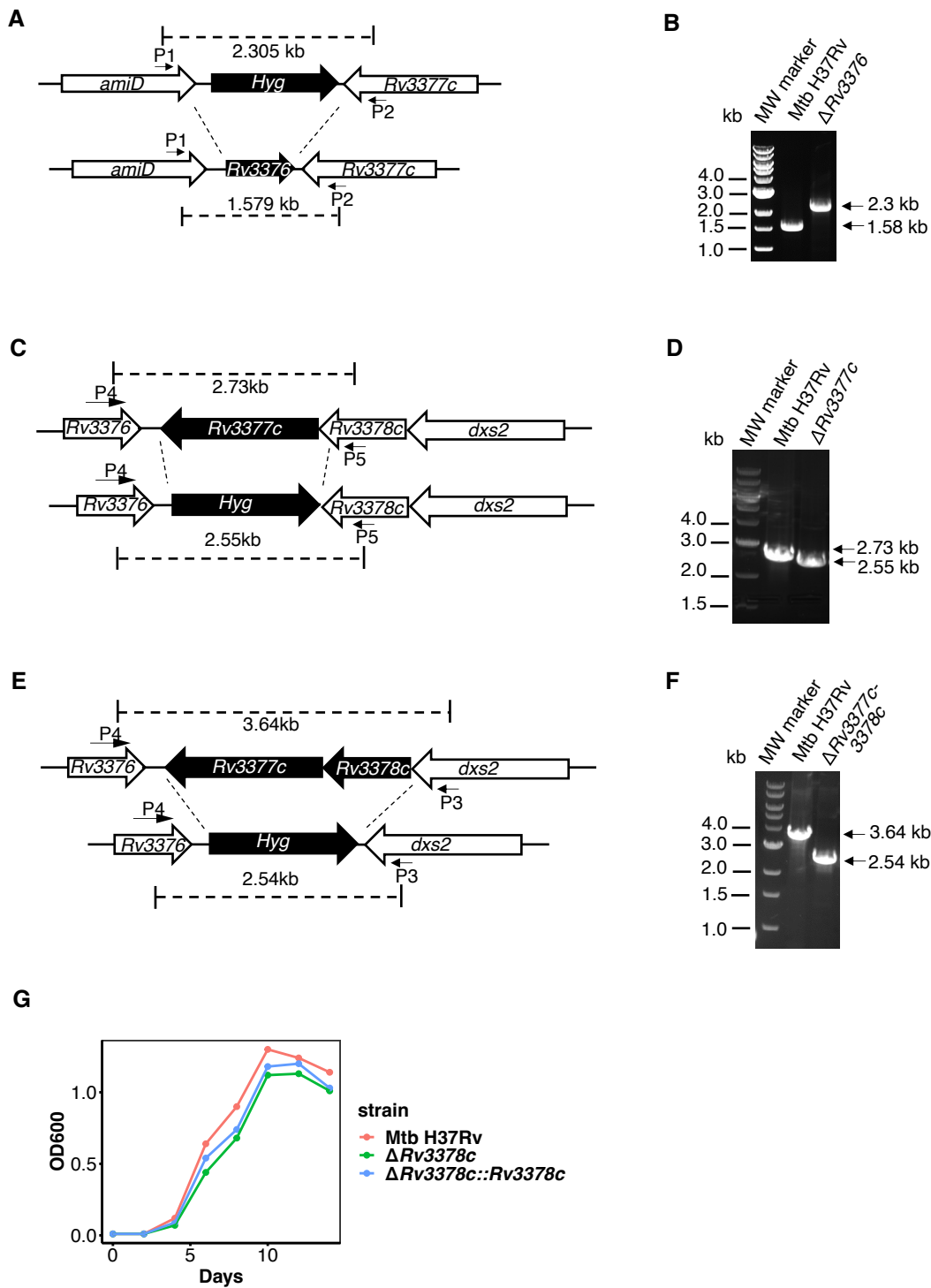

Supplement: S1 Fig — Schematics of the gene replacement are shown for Rv3376 (A), Rv3377c (C), and Rv3377c-Rv3378c (E). Validation by PCR amplification of the gene locus used primer sets flanking the target genes Rv3376 (B), Rv3377c (D), and Rv3377c-Rv3378c (F). Corresponding primer sets are indicated as P1-P2 for Rv3376, P4-P5 for Rv3377c, and P4-P3 for Rv3377c-Rv3378c double mutant. (G) Growth curves of the ΔRv3378c, ΔRv3378c::Rv3378c, and Mtb H37Rv parent strains grown in 7H9 medium are shown. Uncropped gels for S1 Fig BDF and raw data for growth curves are provided in S1 Data and S1 Raw Images. (PDF) [file pbio.3002813.s001.pdf]

**A**

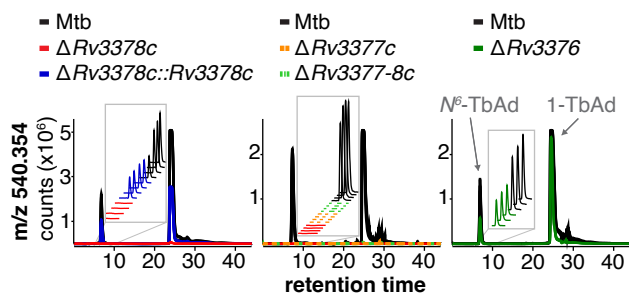

**B**

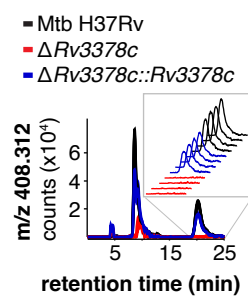

Supplement: S2 Fig — (A) 1- and N6-TbAd (m/z 540.354) are shown in 3 experiments comparing Mtb H37Rv (black), ΔRv3378c (red), and the complemented strain ΔRv3378c::Rv3378c (blue) in a validation experiment (n = 4); or Mtb H37Rv, ΔRv3377c (orange; dashed), ΔRv3378c, and ΔRv3377-8c (gold; dashed) two-gene deletion in an independent experiment (n = 4); or Mtb H37Rv and ΔRv3376 (green) in an analysis of Rv3376 function (n = 3). (B) 1- and N6-tuberculosinyladenine (m/z 408.312) measured in the Mtb H37Rv (black), ΔRv3378c (red), and the complemented strain Δ Rv3378c::Rv3378c (blue). 1-TbAd (A) and 1-tuberculosinyladenine (B) area under the curve were used for statistical testing and to generate Figs 1B and 3A, respectively. (PDF) [file pbio.3002813.s002.pdf]

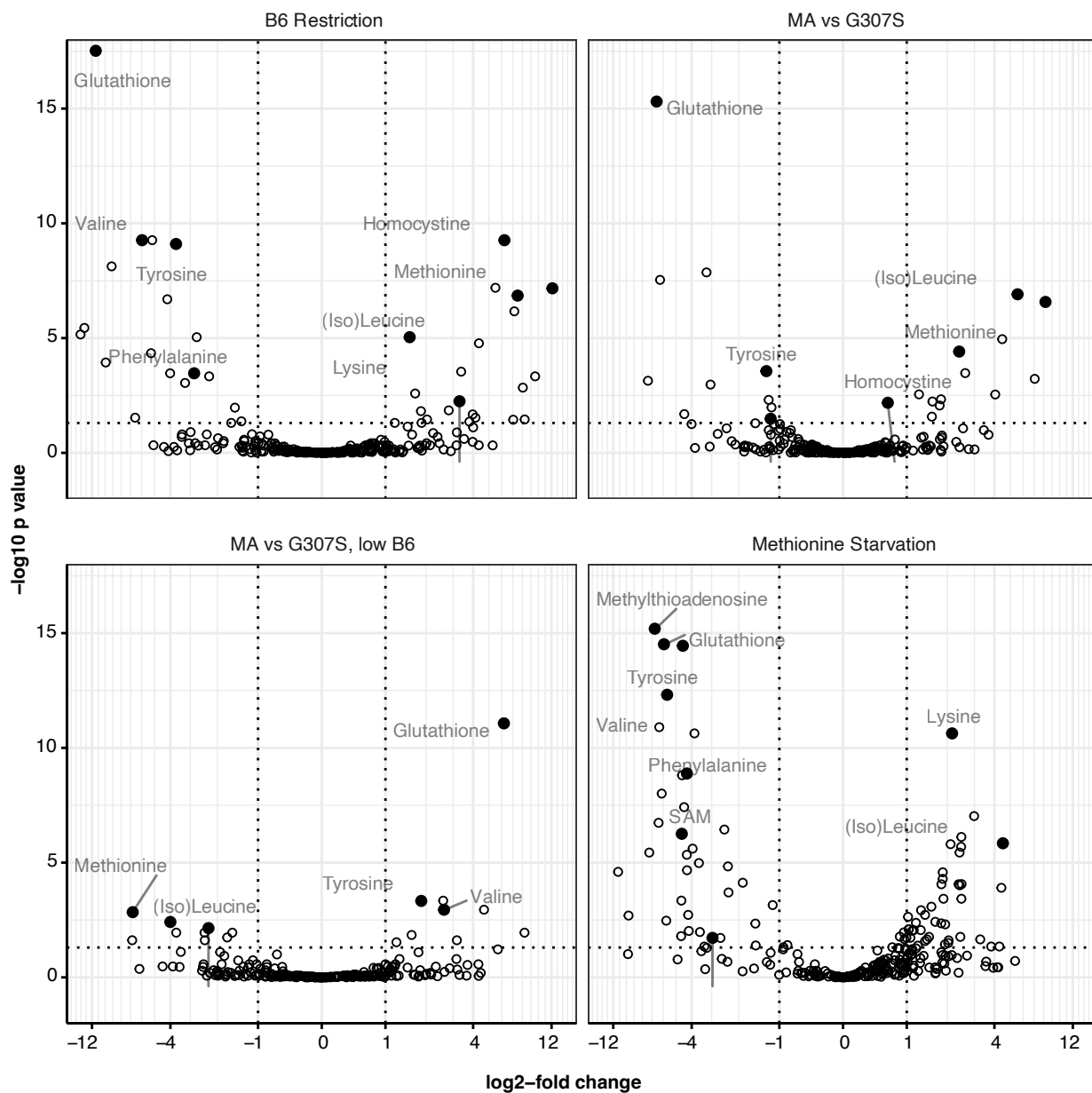

Supplement: S3 Fig — Volcano plots of metabolites altered by disruption of function using a surrogate genetic system expressing the human vitamin B6-dependent enzyme cystathionine beta-synthase (CBS) in S. cerevisiae. Contrasts of the CBS major allele (MA) grown with high (400 ng/ml) or low (1 ng/ml) vitamin B6, compared to the G307S mutation at high (400 ng/ml) or low (1 ng/ml) vitamin B6, or under methionine replete versus starvation conditions. Annotations based on isotopically labeled, pooled standards are shown and tracked across conditions that affected the CBS cofactor, enzyme function, or substrate availability. Data for S3 Fig are provided in S1 Data. (PDF) [file pbio.3002813.s003.pdf]

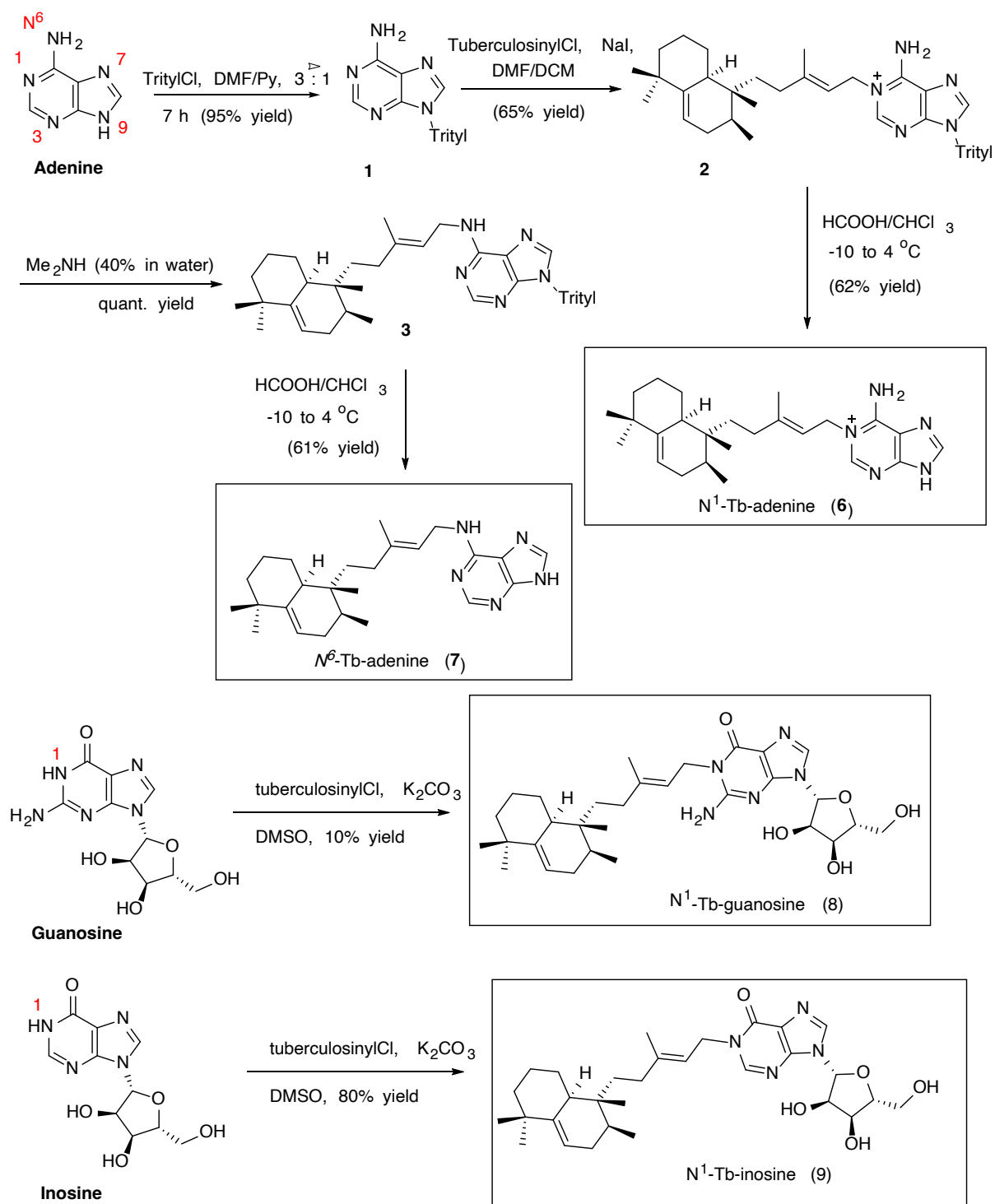

Supplement: S4 Fig — Compounds were characterized by NMR and mass spectrometry and used to identify natural isolates. The synthesis procedures and characterization are provided in S4 Data. (PDF) [file pbio.3002813.s004.pdf]

**A**

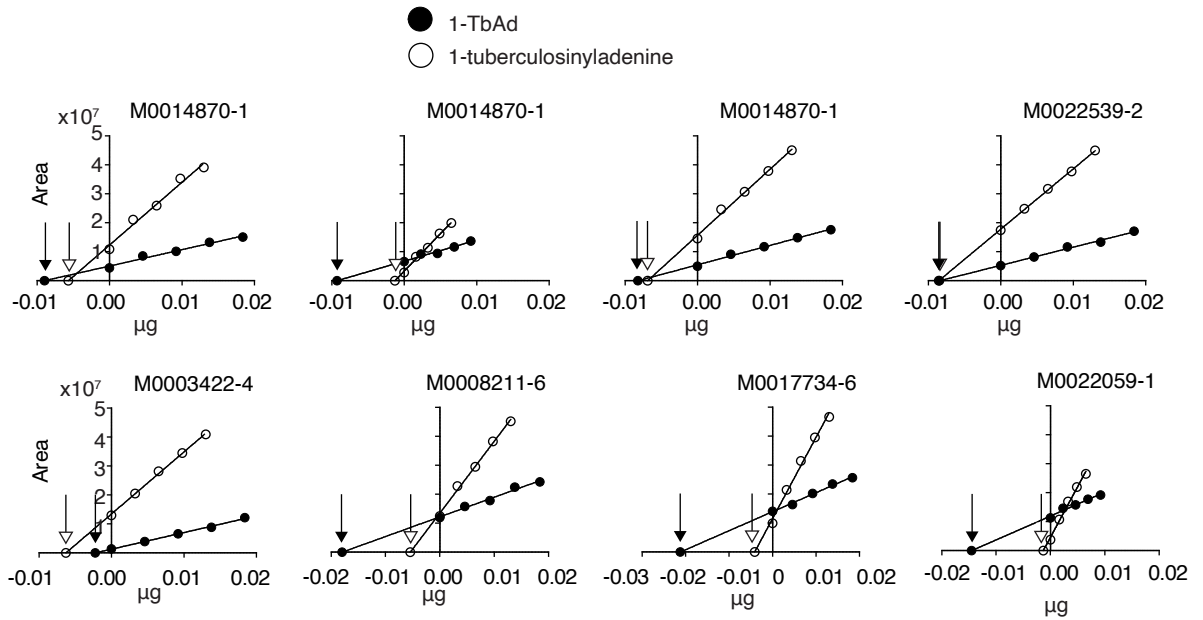

**B**

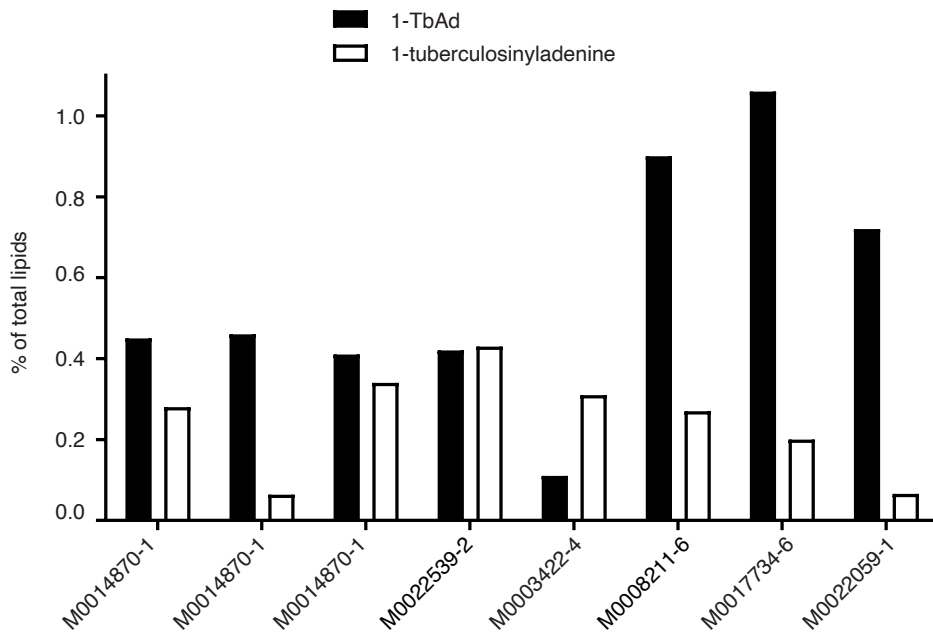

Supplement: S5 Fig — (A) The quantities of natural molecules were estimated by plotting of chromatogram area against the known concentrations of each synthetic compound to obtain the extrapolated number on the x-axis. The arrows pointed to the concentration of the natural molecules. (B) The amount of 1-TbAd and 1-tuberculosinyladenine measured in 8 independent clinical isolates relative to total cellular lipid measured on a balance. The measurements underlying this figure can be found in S1 Data. (PDF) [file pbio.3002813.s005.pdf]

**A**

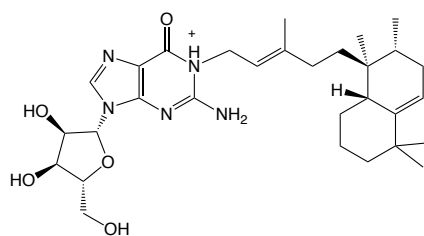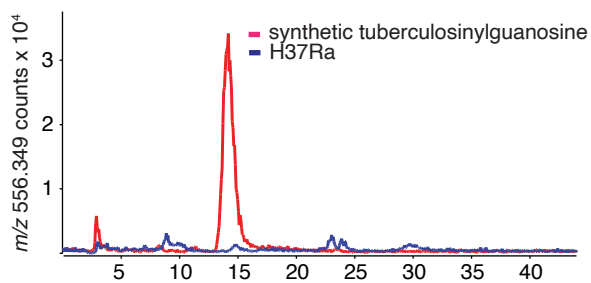

**B**

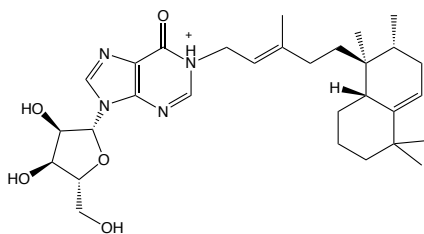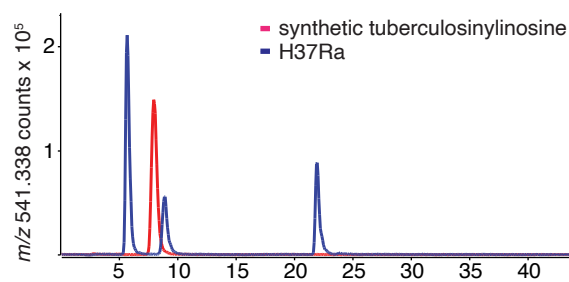

**C**

Negative mode  $[M+HCOO]^-$

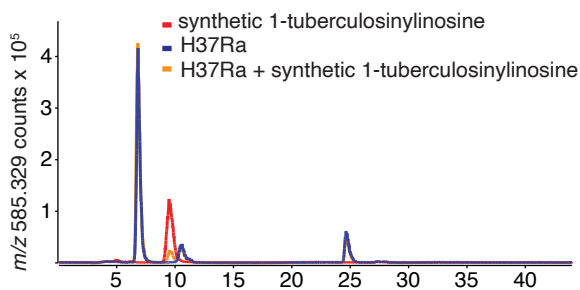

Supplement: S6 Fig — (A–C) The unknown, Rv3378c-dependent masses consistent with tuberculosinylguanosine and tuberculosinylinosine do not match synthetic tuberculosinylguanosine and tuberculosinylinosine. (A) Chemical structure of 1-tuberculosinylguanosine and overlayed extracted ion chromatograms of whole cell lipids from Mtb H37Ra and synthetic 1-tuberculosinylguanosine measured in the positive mode. (B) Chemical structure of 1-tuberculosinylinosine and overlayed extracted ion chromatograms of whole cell lipids from Mtb H37Ra, synthetic 1-tuberculosinylinosine and synthetic 1-tuberculosinylinosine spiked into H37Ra extract, measured using positive (B) and negative (C) mode mass spectrometry. (PDF) [file pbio.3002813.s006.pdf]

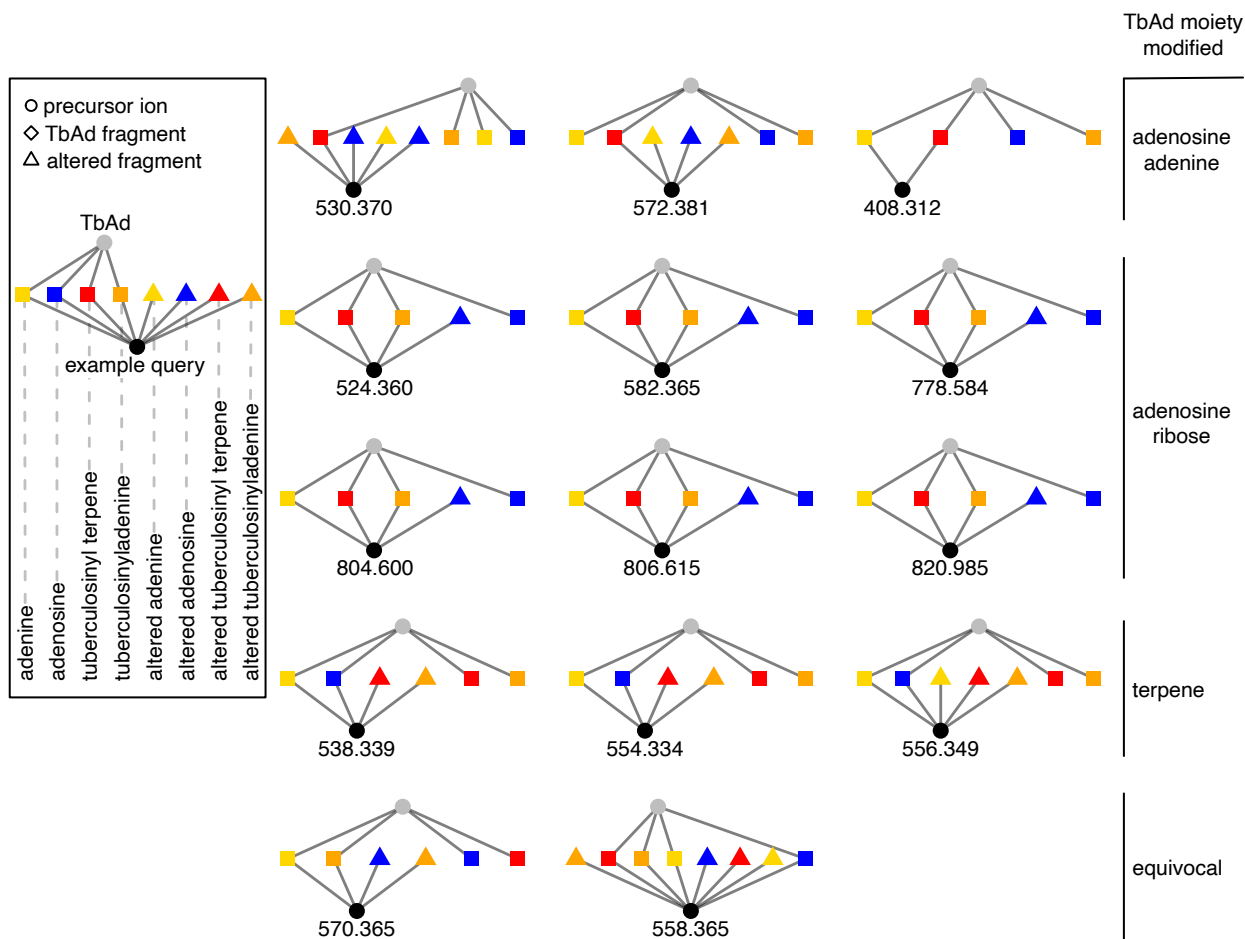

Supplement: S7 Fig — Terpene nucleoside precursors and their fragment ions (observed m/z) after CID-MS were compared to 1-TbAd and the four diagnostic fragments characteristic of CID-MS (calculated m/z) in a pairwise analysis. Only fragments diagnostic of TbAd or modifications of those fragments are shown, with all precursors and fragments within 15 ppm of their calculated mass. Node shapes indicate the precursor and fragment ions; colors indicate adenine, adenosine, tuberculosinyl terpene or tuberculosinyladenine fragments or modifications of those moieties. Vertices connecting precursors and fragments show the presence of shared or unique fragments. (PDF) [file pbio.3002813.s007.pdf]

A

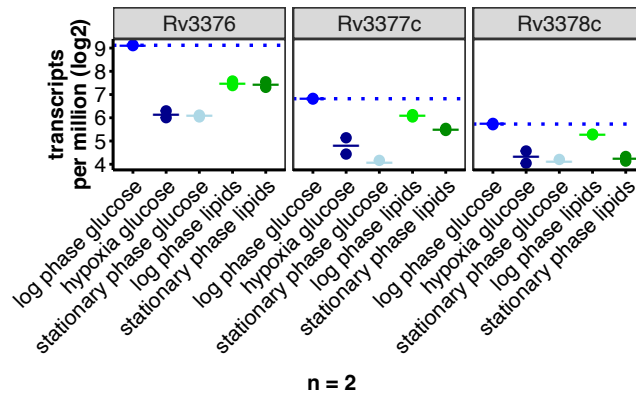

B

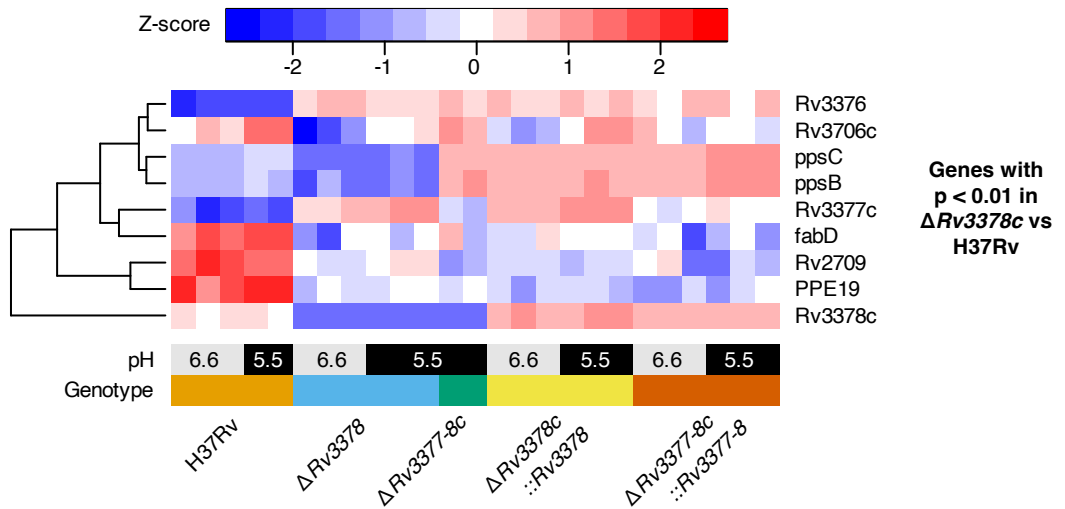

C

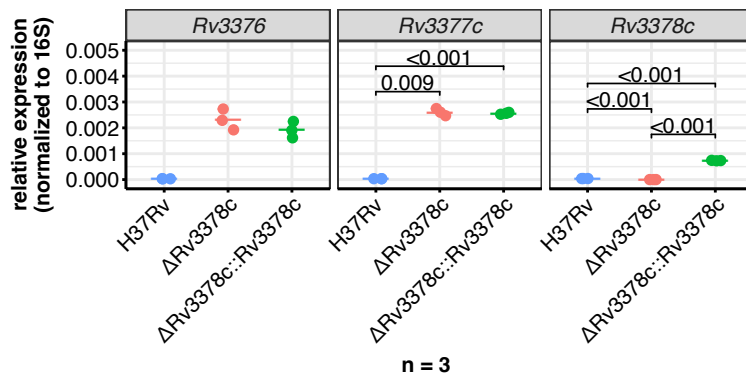

Supplement: S8 Fig — (A) Meta-analysis of published transcriptomics data showed normalized expression of Rv3376, Rv3377c, or Rv3378c in glucose medium during log-phase (blue with blue dotted line to demarcate the reference condition) versus conditions found to repress transcription >4-fold among the 231 unique conditions [39,40]. (B) Heatmap of the most significantly changed genes in a contrast of ΔRv3378c to Mtb H37Rv, with genes clustered by hierarchical clustering after transcriptiomics using RNAseq. (C) Expression of the terpene nucleoside locus genes using quantitative reverse transcription PCR of mRNA in Mtb H37Rv and in the ΔRv3378c or complemented ΔRv3378c::Rv3378c strains, normalized to 16S ribosomal RNA. All pairwise contrasts were tested but only significant p values are shown (t test with Bonferroni correction). The data for S8 Fig can be found in S1 Data; BioProject accession PRJNA1146031 contains raw RNAseq data. (PDF) [file pbio.3002813.s008.pdf]

A

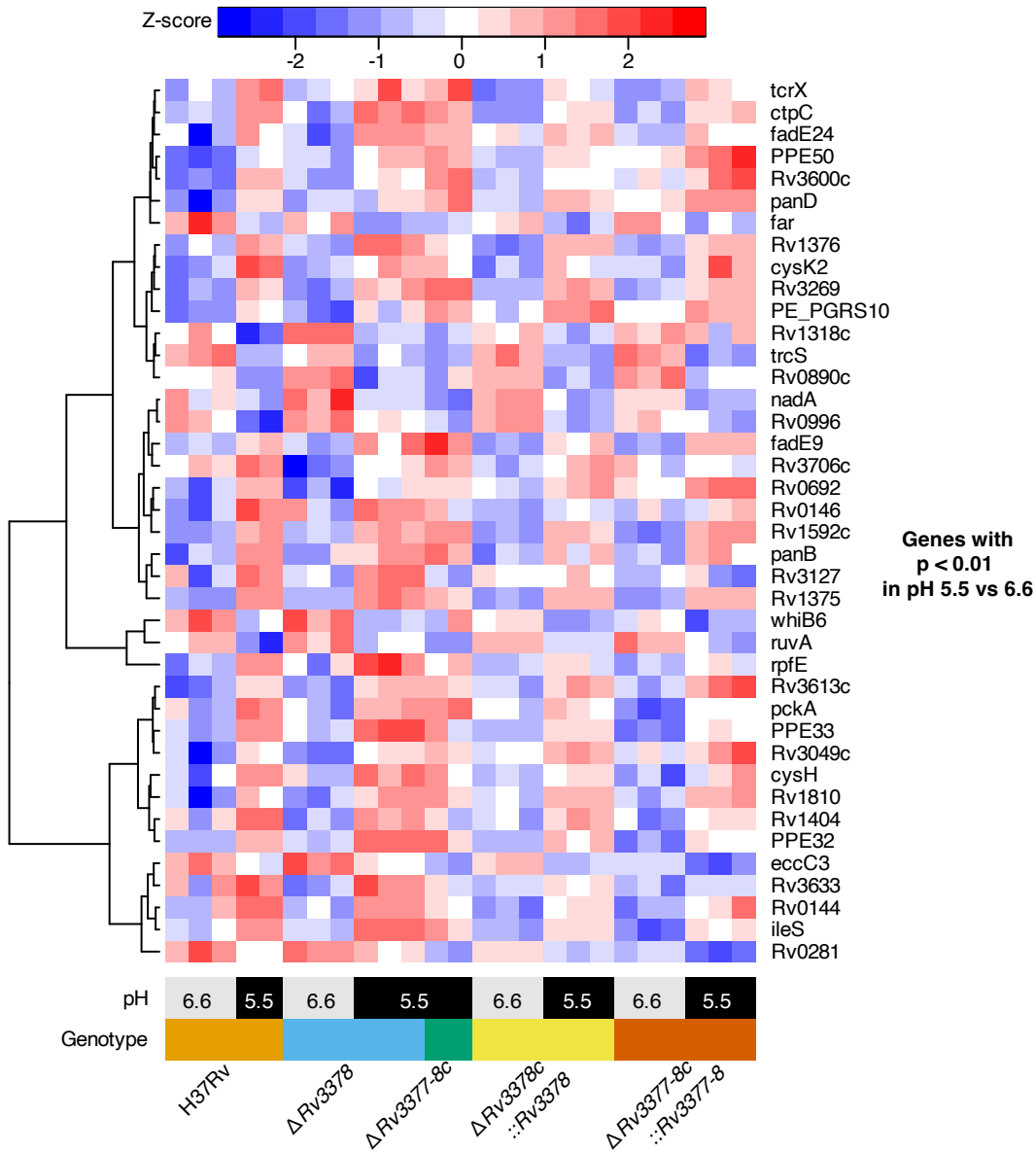

B

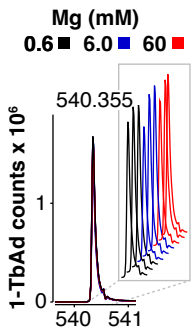

C

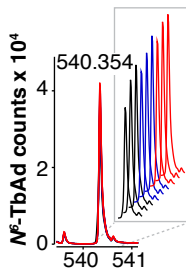

D

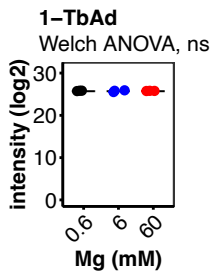

E

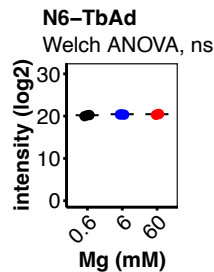

Supplement: S9 Fig — (A) Heatmap of transcripts with p value < 0.01 in a contrast of pH 5.5 versus 6.6 in both the Mtb H37Rv and ΔRv3378c strains measured by RNAseq. (B) Mass spectra of 1-TbAd, m/z 540.354, or N6-TbAd (C), m/z 540.354, from replicate cultures grown in media containing 0.6, 6.0-, or 60-mM magnesium chloride (n = 3 replicates) with observed masses shown. The color key for magnesium concentration was shared for (B–E). Both combined and dithered peaks (inset) are shown to allow visualization of peak correspondence and individual samples. (D) Quantification and statistical analysis of 1-TbAd or N6-TbAd (E) after lipidomics analysis using limms (Benjamini–Hochberg adjusted p value after Welch ANOVA). Raw data for S9 Fig ADE is provided in S1 Data. Raw RNAseq data is available in BioProject accession PRJNA1146031. (PDF) [file pbio.3002813.s009.pdf]

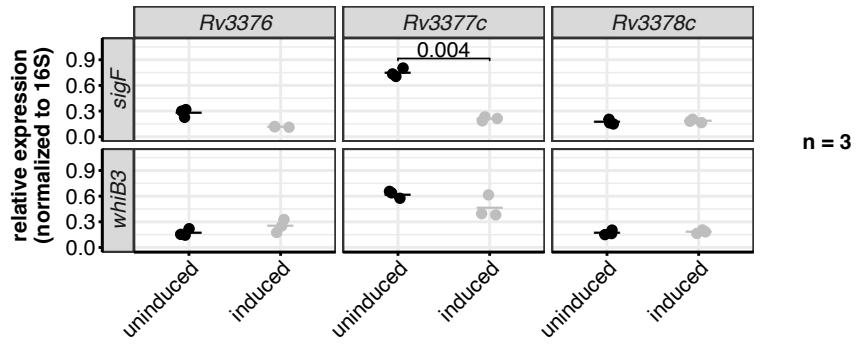

Supplement: S10 Fig — Quantitative PCR analysis of gene expression during transcription factor overexpression. Abundance of terpene nucleoside locus transcripts in strains with inducible overexpression of the transcription factor sigF or whiB3, with anyhydrotetracycline (ATC) induction compared to uninduced conditions. Only significant p values are shown (pairwise t test with Bonferroni correction). The data for this figure are included in S1 Data. (PDF) [file pbio.3002813.s010.pdf]

S1 Fig: Uncropped images of ethidium bromide stained DNA on agarose gels

B

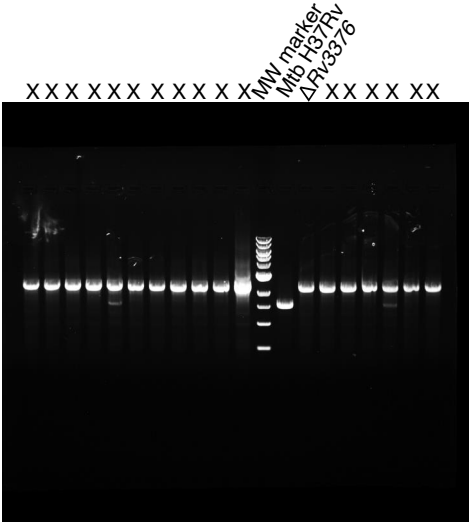

D

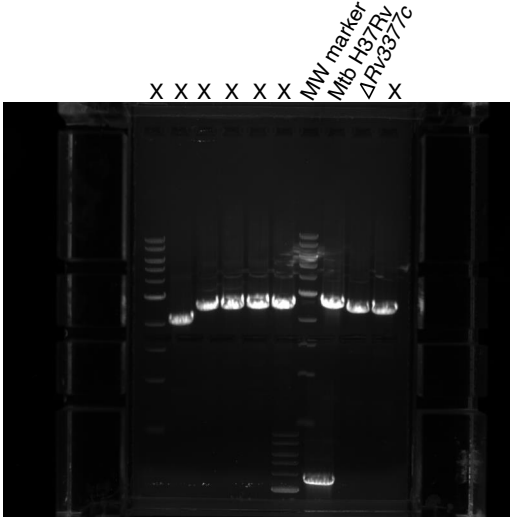

F

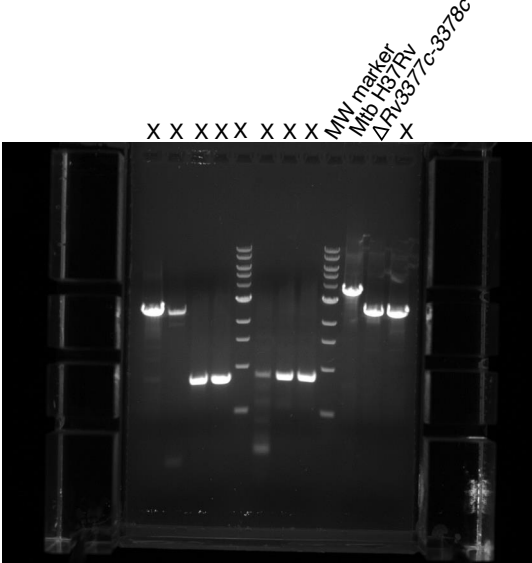

Supplement: S1 Data — The R Markdown document contains annotated R code for computational analyses and generation of manuscript figures. The associated files in the zipped folder contain the data necessary for the analyses. (ZIP) [file pbio.3002813.s013.zip › Mayfield_S1_Data/S1_raw_images.pdf]
