# Supplementary material for: Mycobacteria that cause tuberculosis have retained ancestrally acquired genes for the biosynthesis of chemically diverse terpene nucleosides
Source: PLoS Biol. 2024 Sep 30;22(9):e3002813. doi: 10.1371/journal.pbio.3002813 (PMC11476799; doi:10.1371/journal.pbio.3002813)
Supplement: S1 Table — Data and analyses found in S1 Table are provided in S1 Data. (PDF) [file pbio.3002813.s011.pdf]

putative metabolite	log$_2$-fold change				mean level	F-statistic	p-value	adjusted p-value
	major allele		G307S vs major allele					
	1 vs 400μM B6	-/+ methionine	400μM B6	1μM B6				
glutathione	-11.39	-5.919	-6.559	6.179	21.89	295.1	3.536e-21	9.69e-19
methylthioadenosine	0	-6.733	0	0	31.29	147.6	2.053e-17	2.812e-15
tyrosine	-3.689	-4.534	-1.272	1.856	27.79	107.8	9.665e-16	8.827e-14
valine	-5.969	-5.662	-1.178	2.623	23.38	75.5	6.978e-14	3.824e-12
methionine	12.15	-2.47	8.206	-6.803	22.4	69.64	1.81e-13	8.267e-12
homocystine	6.222	-0.6474	2.394	-2.293	19.93	52.12	5.204e-12	1.584e-10
lysine	1.54	2.15	0.6601	-0.6601	31.85	47.05	1.66e-11	3.789e-10
(iso)leucine	7.486	4.515	5.554	-4.001	27.6	40.96	7.804e-11	1.645e-09
phenylalanine	-2.838	-4.281	-0.4811	1.13	26.17	30.66	1.778e-09	3.48e-08
sam	3.29	-4.603	1.36	-1.873	20.51	28.35	4.026e-09	6.894e-08
