# Supplementary material for: Mycobacteria that cause tuberculosis have retained ancestrally acquired genes for the biosynthesis of chemically diverse terpene nucleosides
Source: PLoS Biol. 2024 Sep 30;22(9):e3002813. doi: 10.1371/journal.pbio.3002813 (PMC11476799; doi:10.1371/journal.pbio.3002813)
Supplement: S2 Table — (PDF) [file pbio.3002813.s012.pdf]

		**gene**	*Rv3376*					*Rv3377c*											*Rv3378c*			
		**coordinate (H37Rv)**	3790459	3790491	3790652	3790688	3790694	3790929	3791117	3791129	3791133	3791136	3791195	3791261	3791576	3791598	3792135	3792263	3792523	3792712	3792840	3793102
		**mutation**	dupT	G>T	C>T	T>A	G>T	T>C	G>A	T>C	C>X	T>C	G>T	A>C	G>T	T>C	T>C	C>A	T>G	CC>TT	A>G	C>A
		**change**	X	V>F	T>M	V>D	G>C	K>E	T>M	X	X15	I>V	T>K	I>S	P>H	T>A	M>V	G>V	I>L	X	V>A	D>Y
**MTb complex species**	**total genomes**	**synteny (%)**	**Number of genomes with mutation**																			
africanum	34	101	2		4		4		1				1		1			29				1
bovis	130	99									129			1		1			1		50	
orygis	2	101						1														
caprae	4	100									4	4										
microti	6	100				6				1												
pinnipedii	3	100		3		3																
mungi	1	99															1			1		
canetti	14	97	100% coverage; 99% identity																			
lacus	2	43	97% coverage; 77% identity																			
decipiens	1	52	97% coverage; 79% identity																			
