# Supplementary material for: Mycobacteria that cause tuberculosis have retained ancestrally acquired genes for the biosynthesis of chemically diverse terpene nucleosides
Source: PLoS Biol. 2024 Sep 30;22(9):e3002813. doi: 10.1371/journal.pbio.3002813 (PMC11476799; doi:10.1371/journal.pbio.3002813)
Supplement: S2 Data — The limms package vignette provided in html format. The complete R Markdown used to produce the html document is part of the limms package available at https://github.com/jamayfie/limms. (ZIP) [file pbio.3002813.s014.zip › LIMMS_vignette.html]

LIMMS\_vignette


# LIMMS\_vignette

#### Jacob A. Mayfield

- 0.1 Introduction
- 0.2 Starting limms
- 0.3 Data Quality Control
- 0.4 Preprocessing and
  Normalization
- 0.5 Building Contrasts
- 0.6 Differential Abundance
  Analyses
- 0.7 Database Matching
- 0.8 Data Visualizations
- 0.9 Version Info

```
#> Warning: replacing previous import 'aroma.light::normalizeRobustSpline' by
#> 'limma::normalizeRobustSpline' when loading 'limms'
#> Warning: replacing previous import 'data.table::melt' by 'reshape2::melt' when
#> loading 'limms'
#> Warning: replacing previous import 'data.table::dcast' by 'reshape2::dcast'
#> when loading 'limms'
```

## 0.1 Introduction

The R package `limms`provides functions for both
unsupervised and supervised analyses of mass spectrometry data. More
specifically, it provides functions for exploratory data analysis,
quality control, preprocessing, and normalization of mass spectrometry
data. It also facilitates contrast-based analyses to identify
compounds/molecules (e.g., adducts, metabolites) that are differentially
abundant under different conditions (e.g., treatment/control, cell
type). Study designs with multiple conditions benefit from the ability
to specify the contrasts of interest, rather than rely on pairwise or
one-vs-all comparisons. `limms` adapts the excellent
`limma` package for transcriptomics to mass spectrometry. In
addition to providing a portal to `limma` that maintains mass
spectrometry peak information, `limms` provides the functions
necessary to coerce mass spectrometry data into a format readable by
`limma`. Downstream of differential abundance analysis,
`limms` provides a function to match masses to a
user-provided list of knowns.

`limms` accepts as input a table of *m/z* values
that can come from any mass spectrometry platform or peak finding
algorithm, as long as the rows correspond to mass peaks and columns to
samples. It outputs a `limma` object, or can append a
`limma` topTable to the input peak table. This output
includes the adjusted p-value and fold-change needed to flag the
compounds that differ significantly under a contrast of interest. This
shorter list of changed *m/z*’s is easily amenable to data
visualization and analysis, for example using heatmaps. A plot of 10,000
metabolites is hard to read, but a heatmap of 50 metabolites that differ
significantly between classes is useful. This vignette includes examples
of suggested data visualizations facilitated by `limms`.

This vignette demonstrates the `limms` package in R using
the example CBS dataset provided with the package, which consists of
metabolite abundances in *Saccharomyces cerevisiae* after
complementation with human cystathionine beta-synthase (CBS) allele. All
of the functions in `limms` are used in the vignette, plus
several data visualizations that take advantage of the
`limms` output. The vignette also corresponds to the help
files for the `limms` package, but provides additional
explanations. In contrast, the help files for individual
`limms` functions may provide additional analyses not in the
vignette, especially for quality control.

- The CBS data are in the dataset
  `CBS.xcms_diffreport`.
- A metabolite database file from the Human Metabolome Database
  corresponding to compounds included as internal controls in the mass
  spectrometry experiments is in the dataset
  `hmdb_Shortlist`.
- A topTable object of metabolites is produced by the code in this
  vignette, but is also included in the `limms` package as the
  dataset `ttF.CBS` for convenience.

## 0.2 Starting limms

An example pipeline from a table of *m/z* intensities to a
significance table with putative identifiers is provided here. Start by
loading `limms`.

The example data used the excellent `xcms` package for
peak picking, grouping, and retention time alignment, with output as a
`diffreport` object of aligned peaks in rows and sample
intensities in columns. The `diffreport` also contains mass
and retention time columns that can be passed through the
`limms` functions, although only the intensities are needed.
`xcms` or a `diffreport` are recommended but not
required; however, the mass spectrometry data must be read in as a data
frame with the peak intensities listed as columns.

Data input can be in any format, but `limmms` will
internally convert to a data.table object to use functions from the
`data.table` package. Some `limms` functions allow
arguments that specify which columns contain names, intensities, or
information. Peak names, i.e., the `xcms` mz@rt labels, can be specified and
used, or if the rows are unspecified, `limms` will number the
rows for consistent tracking. For the CBS dataset, columns 24 to 55
contain the peak data. There are additional columns of data from
controls with isotopically labeled standards not used here.

## 0.3 Data Quality Control

The `limms` function `scatterQC` generates two
data visualizations that can help to assess overall data quality, how
sample classes are similar or different, and whether any samples are
drastically different. The function displays the total number of
non-zero ions in a dot plot and the intensity measurements in a boxplot.
The samples can be grouped according to user-defined groupings, such
that different experimental covariates, mass spec run order, or
arbitrary groupings can be examined. The 5%, 25%, 50%, 75%, and 95%s
quantiles across all samples are plotted as grey reference lines, and
outliers can be displayed if desired.

These plots allow for exploratory data analysis that may suggest
strategies for improving the downstream results. Differential abundance
analysis relies on differences between sample classes, which may or may
not be visible in `scatterQC` plots. However, these plots are
intended to flag outlier samples, check for changes in quality over the
course of a mass spectrometry run, or to identify sample classes that
are different enough that peak picking and alignment may have suffered.
In some cases, users may want to repeat the pick peaking step without
problematic samples rather than simply excluding them from
`limms` analysis.

To group the data, make a covariates object for the
`CBS.xcms_diffreport` data set.

```
# Extract the data columns from CBS.xcms_diffreport, 
# an xcms diffreport object included as limms package data,

desMetB6 <- cbind(names(CBS.xcms_diffreport[,24:55]), c(rep("CBS",4), rep("CBS",4), rep("CBS",4), 
rep("CBS",4), rep("CBS",4), rep("CBS",4), rep("G307S",4), rep("G307S",4)), 
c(rep("Yes",4), rep("Yes",4), rep("Yes",4), rep("Yes",4), rep("No",4), rep("No",4),
rep("Yes",4), rep("Yes",4)),  c(rep("High",4), rep("High",4), rep("Low",4), 
rep("Low",4), rep("High",4), rep("Low",4), rep("High",4), rep("Low",4)), c(rep(2,4),
rep(1,4), rep(2,4), rep(1,4), rep(1,4), rep(1,4), rep(2,4), rep(2,4)))
desMetB6 <- data.frame(desMetB6)
names(desMetB6) <- c("Run", "Strain", "Met", "B6", "Rep")

knitr::kable(desMetB6[1:5,], caption="Subset of the CBS covariates table")
```

Subset of the CBS covariates table

| Run | Strain | Met | B6 | Rep |
| --- | --- | --- | --- | --- |
| ORB17928 | CBS | Yes | High | 2 |
| ORB17935 | CBS | Yes | High | 2 |
| ORB17942 | CBS | Yes | High | 2 |
| ORB17949 | CBS | Yes | High | 2 |
| ORB17900 | CBS | Yes | High | 1 |

Plot the data, first by run order, then by methionine
supplementation.

```
# QC plot of all samples
# this can be useful for checking run order, etc
scatterQC(CBS.xcms_diffreport[, c(24:55)], seed=75, main="QC of all samples")
#> [1] "Warning: scatterQC performs a log transformation: input data should not be log transformed."
```

```
# QC plot by group
# Treatments are expected to differ... but it's still useful to know which ones!
# flag outlier samples
scatterQC(CBS.xcms_diffreport[, c(24:55)], seed=75, groupNames=desMetB6$Met, 
  main="QC by methionine addition", outlayer=TRUE)
#> [1] "Warning: scatterQC performs a log transformation: input data should not be log transformed."
```

## 0.4 Preprocessing and Normalization

Before statistical analysis such as clustering or inference of
differential abundance, the intensity data benefit from:

- having zeros removed
- being log transformed
- being normalized

The `limms` function `imputeZerosUnifMin`
imputes zeros, replacing them with random values between 1 and the
actual sample minima. This was empirically determined to give reasonable
results, and works under the assumption that the measured minima reflect
the threshold of detection of the instrument. Users of `xcms`
will be familiar with the `fillPeaks` family of functions
that also aim to replace zeros, using a different method.
`imputeZerosUnifMin` works well with `fillPeaks`,
and removes any remaining zeros. `imputeZerosUnifMin` also
performs log2 transformation by default, although transformation can be
turned off using the `output="imputed"` argument.

Impute the zeros and log2 transform columns 24 to 55. To store the
imputed values, call to a new object.

```
all.i_l <- imputeZerosUnifMin(CBS.xcms_diffreport, intensities=24:55, seed=478)
```

**An important note:** because **random values
below the minima are used for imputation**, the resulting object
will be different each time `imputeZerosUnifMin` is called.
Differences between calls are minor and affect compounds at or below the
threshold for reliable measurement. Nonetheless, it can be convenient to
have the same imputed values each time the analysis is run. This can be
accomplished by specifying the seed to feed
`imputeZerosUnifMin` inside the function using the
`seed` argument, or like this:

```
set.seed(2846)
```

where 2846 is a user chosen number, which should be reset to a
different number for other, independent analyses.

Knowing the imputed compounds is important. To generate a new table
with information about impounded measurements, the impounded
measurements can be flagged and counted.

```
metimp <- t(sapply(1:dim(CBS.xcms_diffreport)[1], function(i) CBS.xcms_diffreport[i, 24:55]==0))
```

```
numimp <- sapply(1:dim(CBS.xcms_diffreport)[1], function(i) length(which(metimp[i,]=="TRUE")))
```

```
# Quick QC according to the number of impounded measurements
# which peaks have no samples with impounded zeros?
summary(numimp == 0)
#>    Mode   FALSE    TRUE 
#> logical     104     170
# which peaks have > 5 impounded?
summary(numimp > 5)
#>    Mode   FALSE    TRUE 
#> logical     241      33
```

For quick QC according to the number of impounded measurements,
generate a table with information about impounded measurements including
how many and which samples were impounded for each metabolite

```
lcids <- sapply(1:dim(CBS.xcms_diffreport)[1], function(i) 
  paste(as.character(
    colnames(CBS.xcms_diffreport[, 24:55])[which(CBS.xcms_diffreport[i, 24:55]==0)]),
    collapse="; "))

xi <- data.table(cbind(numimp,lcids))
head(xi)
#>    numimp
#> 1:      9
#> 2:      5
#> 3:      2
#> 4:      0
#> 5:      0
#> 6:      0
#>                                                                                       lcids
#> 1: ORB17900; ORB17902; ORB17909; ORB17916; ORB17923; ORB17903; ORB17910; ORB17917; ORB17924
#> 2:                                         ORB17929; ORB17902; ORB17909; ORB17903; ORB17910
#> 3:                                                                       ORB17907; ORB17908
#> 4:                                                                                         
#> 5:                                                                                         
#> 6:
```

The `limms` function `runNorm` normalizes the
data across sample classes. Three methods for normalization are provided
by setting the argument `method` to

- “FullQuantileNorm” for normalization by full quantiles,
- “globalScalingNorm” for scaling by the 75% quantile,
- “pc1Norm” for normalization by the first principal component.

A fourth method, “custom” allows a custom normalization method to be
used by setting FUN=your\_custom\_method.

“FullQuantileNorm” produced the best results for test datasets and is
the default method.

Is normalization necessary? There may be experiments/cases where it
is not advised; however, sample-to-sample technical variation, typical
variation between biological replicates, and mass spectrometry issues
like chromatographic drift and loss of sensitivity over a multi-sample
run can confound differential abundance analyses and inflate false
positive and false negative rates. Normalization helps remove unwanted
technical effects, so that differences in intensities reflect biological
effects of interest. Batch normalization is not part of
`limms`, but can be implemented via `limma` or
through `ComBat` in the `sva` package.

```
all.fq <- runNorm(all.i_l, intensities=70:101)

# NOTE: you can also call intensities by pattern match
all.fq <- runNorm(all.i_l, intensities=grep("log2", names(all.i_l)))

# or by name
all.fq <- runNorm(all.i_l, intensities=names(all.i_l[, .SD, .SDcols=patterns("log2")]))

# which has advantages in making code portable if something changes
# or you want to use as a template
```

Normalize by other methods as a comparison

```
# Normalization by global scaling
all.gs <- runNorm(all.i_l, intensities=70:101, method="globalScalingNorm")

# Normalization by principal components
all.pc1 <- runNorm(all.i_l, intensities=70:101, method="pc1Norm")

# Normalization using a custom function, scaling to the median
# For demonstration of a custom function only: this method is not recommended.
medPeak <- function(x)
      {
        # x[, .(scale(x, center=TRUE, scale=FALSE))]
        x[, .(scale(x, center = (unlist(x[, .(lapply(.SD, function(i)
          stats::median(unlist(i, use.names = FALSE)))), .SDcols = names(x)]) - 
            unlist(x[, .(stats::median(unlist(.SD, use.names = FALSE))),
            .SDcols = names(x)], use.names = FALSE)), scale = FALSE)
          )]
      }

all.med <- runNorm(all.i_l, intensities=70:101, method="custom", FUN=medPeak)
```

What did the normalization do?

```
# add a color key
# methionine starvation induced the largest changes
# make the methionine starvation condition pale green
colCBS <- data.table(desMetB6)

# Boxplots of log intensities

all.fq.melt <- data.table(reshape2::melt(all.fq, id.vars=1:69, value.name="intensity", 
 variable.name="sample"))
all.fq.melt[, Run := gsub("\\..*$", "", sample)]
all.fq.melt[, transform := gsub("^.*\\.", "", sample)]
all.fq.melt[, transform := gsub("norm", "quantile", transform)]

all.fq.melt <- merge(all.fq.melt, colCBS, by="Run", sort=FALSE)

ggplot(all.fq.melt, aes(x=fct_inorder(Run), y=intensity)) +
  geom_boxplot(aes(fill=Met)) +
  facet_wrap(~transform, scales="free_x") +
  theme(axis.text.x=element_text(angle=90, vjust=0.5)) +
  xlab("sample") +
  ggtitle("comparison of log2 transformed to full quantile normalized data")
```

Comparison of normalization methods.

```
norm.list <- list(quantile=all.fq[, 102:133], global_scaling=all.gs[, 102:133], 
 pc1=all.pc1[, 102:133], median=all.med[, 102:133])
all.norm.melt <- data.table(reshape2::melt(norm.list, value.name="intensity", 
 variable.name="sample"))
#> No id variables; using all as measure variables
#> No id variables; using all as measure variables
#> No id variables; using all as measure variables
#> No id variables; using all as measure variables

all.norm.melt[, Run := gsub("\\..*$", "", sample)]
all.norm.melt[, transform := gsub("^.*\\.", "", sample)]
all.norm.melt[, transform := gsub("norm", "quantile", transform)]
all.norm.melt <- merge(all.norm.melt, colCBS, by="Run", sort=FALSE)

ggplot(all.norm.melt, aes(x=fct_inorder(Run), y=intensity)) +
  geom_boxplot(aes(fill=Met)) +
  facet_wrap(~L1, scales="free") +
  theme(axis.text.x=element_text(angle=90, vjust=0.5)) +
  xlab("sample") +
  ggtitle("comparison of normalization methods")
```

Normalization by the first principal component alters the scale of
the abundance measures, making comparisons to other methods difficult.
In context of normalization, it is often more informative to compare
relative log expression (RLE) measures, where the RLE of a given
compound in a given sample is the log ratio of the intensity to the
median intensity of the compound across samples. `relLog` is
a `limms` internal function for computing RLEs.

```
norm.list <- list(
  quantile=relLog(all.fq, intensities=102:133)[, .SD, .SDcols=patterns("rle")],
  global_scaling=relLog(all.gs, intensities=102:133)[, .SD, .SDcols=patterns("rle")],
  pc1=relLog(all.pc1, intensities=102:133)[, .SD, .SDcols=patterns("rle")],
  median=relLog(all.med, intensities=102:133)[, .SD, .SDcols=patterns("rle")])
all.norm.melt <- data.table(reshape2::melt(norm.list, value.name="intensity", 
 variable.name="sample"))
#> No id variables; using all as measure variables
#> No id variables; using all as measure variables
#> No id variables; using all as measure variables
#> No id variables; using all as measure variables
all.norm.melt[, Run := gsub("\\..*$", "", sample)]
all.norm.melt <- merge(all.norm.melt, colCBS, by="Run", sort=FALSE)

ggplot(all.norm.melt, aes(x=fct_inorder(Run), y=intensity)) +
  geom_boxplot(aes(fill=Met)) +
  facet_wrap(~L1, scales="free") +
  theme(axis.text.x=element_text(angle=90, vjust=0.5)) +
  xlab("sample") +
  ggtitle("comparison of normalization by relative log expression")
```

## 0.5 Building Contrasts

Since `limms` relies on `limma` for
differential abundance analysis, it adopts the `limma` input
structure. As a contrast-based method, `limms` requires input
of R objects to specify the experimental design and contrasts to make,
in addition to the mass spectrometry data.

These objects can be built in different ways. The `limma`
manual and vignette include a detailed, formal discussion of these
objects that is required reading to understand how the output is
generated and what interpretations can be made. The `stats`
function `model.matrix` and `limma` function
`makeContrasts` are helpful to build the correct objects. Be
aware that the default behaviors for `model.matrix` and
`makeContrasts` may add intercept terms, not encode the
desired contrast, or include contrasts that are neither of interest nor
necessary.

Here, a design matrix and a contrast matrix for the CBS dataset is
used as an example.

First, we detail the experimental design. For the CBS dataset, the
variables are:

- Run = the mass spectrometry sample run number,
- Strain = whether the cells had the major CBS allele or the
  nonfunctional G307S allele,
- Met = whether methionine was added to the medium to stimulate CBS
  flux,
- B6 = whether vitamin B6 was added at optimal or limiting
  concentration,
- Rep = whether the cells belonged to replicate batch 1 or 2.

These are contained in `desMetB6`, the covariate table
used for `scatterQC` above.

Now build the design matrix.

Start by choosing the covariates on which to base the contrasts:

```
f <- paste(desMetB6$Strain, desMetB6$Met, desMetB6$B6, desMetB6$Rep, sep="")
f <- factor(f)
```

The `stats` function `model.matrix` is useful
for converting the factor levels data into a design matrix.

```
desCu <- model.matrix(~0+f)
colnames(desCu) <- levels(f)

knitr::kable(desCu[1:5, ], caption="Subset of the CBS design matrix")   # Check the design matrix.
```

Subset of the CBS design matrix


| CBSNoHigh1 | CBSNoLow1 | CBSYesHigh1 | CBSYesHigh2 | CBSYesLow1 | CBSYesLow2 | G307SYesHigh2 | G307SYesLow2 |
| --- | --- | --- | --- | --- | --- | --- | --- |
| 0 | 0 | 0 | 1 | 0 | 0 | 0 | 0 |
| 0 | 0 | 0 | 1 | 0 | 0 | 0 | 0 |
| 0 | 0 | 0 | 1 | 0 | 0 | 0 | 0 |
| 0 | 0 | 0 | 1 | 0 | 0 | 0 | 0 |
| 0 | 0 | 1 | 0 | 0 | 0 | 0 | 0 |

Now build the contrast matrix.

While many contrasts are possible for the CBS data, only four are
interesting. - `MA_B6` compares cells with the major allele
at high versus low concentrations of the CBS cofactor vitamin B6; -
`Strain_w_B6` compares the major allele to G307S at high
vitamin B6; - `Strain_by_B6` is a compound contrast comparing
metabolites that vary with both B6 concentration and allele; and -
`CBS_Met` compares the major allele under methionine replete
versus starvation medium to contrast growth restriction due to low
pathway flux to a CBS block.

```
contMatrix <- makeContrasts(MA_B6="(CBSYesLow1+CBSYesLow2)-(CBSYesHigh1+CBSYesHigh2)", 
    Strain_w_B6="G307SYesHigh2-CBSYesHigh2",
    Strain_by_B6="(G307SYesLow2-G307SYesHigh2)-(CBSYesLow2-CBSYesHigh2)",
    CBS_Met="CBSNoHigh1-CBSYesHigh1", levels=desCu)

knitr::kable(contMatrix, caption="CBS contrast matrix") # Check the contrast matrix.
```

CBS contrast matrix

|  | MA\_B6 | Strain\_w\_B6 | Strain\_by\_B6 | CBS\_Met |
| --- | --- | --- | --- | --- |
| CBSNoHigh1 | 0 | 0 | 0 | 1 |
| CBSNoLow1 | 0 | 0 | 0 | 0 |
| CBSYesHigh1 | -1 | 0 | 0 | -1 |
| CBSYesHigh2 | -1 | -1 | 1 | 0 |
| CBSYesLow1 | 1 | 0 | 0 | 0 |
| CBSYesLow2 | 1 | 0 | -1 | 0 |
| G307SYesHigh2 | 0 | 1 | -1 | 0 |
| G307SYesLow2 | 0 | 0 | 1 | 0 |

All objects are now ready for testing. The design matrix
`desCu` and contrast matrix `contMatrix` are used
to set the `des` and `cont` arguments,
respectively, in the function `limmaTest`.

## 0.6 Differential Abundance Analyses

Once the design and contrast matrices are built and the data are
transformed into a `limma`-useable format, you could begin
the analysis procedure outlined in the `limma` vignette.
Alternatively, as a streamlined procedure,`limms` provides
the function `limmaTest`.

By combining the `limma` functions `lmFit`,
`contrasts.fit` and `eBayes`,
`limmaTest` focuses on the functions most appropriate to mass
spectrometry data. Using `limmaTest` also allows peak
information columns like mass and retention time to be passed to the
output.

Different types of study design can be handled through
`limmaTest` by specifying a contrast matrix and/or block
structure. A contrast matrix is not required; however, all contrasts
from the design matrix are tested if a contrast matrix is not provided,
which is desirable only in some cases. Likewise, `limmaTest`
allows specification of blocks to call the
`duplicateCorrelation` function for cases where this is
needed. A tabular summary shows how `limma` functions can be
accessed using `limmaTest` arguments.

**Summary of contrast-based options in
`limmaTest`:**

| limmaTest arguments | limma functions used | usage case(s) |
| --- | --- | --- |
| cont=NULL  block=NULL | `lmFit`  `eBayes` | simple contrast;  paired samples;  all contrasts |
| cont specified  block=NULL | `lmFit`  `contrasts.fit`  `eBayes` | select contrasts;  compound contrasts |
| cont=NULL  block specified | `lmFit`  `duplicateCorrelation`  `eBayes` | duplicated samples;  blocks |
| cont specified  block specified | `lmFit`  `duplicateCorrelation`  `contrasts.fit`  `eBayes` | select paired;  select within block |

Please refer to the `limma` help page for
`lmFit` and/or the `limma` manual if this is
unclear or for additional examples of design and contrast objects for
different study designs.

The `limmaTest` output can also be directed to a
`topTable` object that is appended to the input matrix. This
is useful to get the peaklist data (mass, retention time) and
statistical inference data (Benjamini-Hochberg adjusted p-value,
log-fold-change) in one place, and is the default output. The
`limma` `eBayes` output is available by setting
`limma_out=TRUE`, which can be useful to use native
`limma` plotting functions like QQ-plots.

The default output calls `topTable` using the entire
contrast matrix; hence, if more than one contrast is present, the
adjusted p-value returned will be based on an F-test across all
contrasts. This is useful in some cases, but if the desired output is
for one specific contrast, set the `coef` argument to the
contrast of interest using the name of the desired contrast or its
number in the contrast matrix.

Here, use the CBS data to input the data, design matrix and contrast
matrix.

```
# Choose the peak names and normalized intensity columns from a data.table like all.fq,
# and all other columns will be passed to the output,
# or enter a matrix of intensities only and row names will be passed to the output.
# Results are appended to the input as a limma topTable.
# The default behavior is to return the p-value for all contrastas in the adj.P.Val column.
# This is an F-score if more than one contrast is present in the contrast matrix.

tT.F <- limmaTest(x=all.fq, peaknames="name", intensities=102:133, des=desCu, 
  cont=contMatrix)
#> [1] "For data.table input, assign columns using the 'peaknames' and 'intensities' arguments"
#>        MA_B6 Strain_w_B6 Strain_by_B6 CBS_Met
#> Down      18          11            8      36
#> NotSig   233         249          257     193
#> Up        23          14            9      45
```

Warning: the `adj.P.Val` column returned in the output may
be a p-value or an F-statistic depending on the design, contrasts and
output requested.

Results are most easily accessed through creation of
`limma` `topTable` objects. The
`limmaTest` default is to append these to the input data.
Each contrast has its own `topTable`. For example,
significant hits for the first contrast, `MA_B6`, are seen
with

```
tt1 <- limmaTest(x=all.fq, peaknames="name", intensities=102:133, des=desCu, 
  cont=contMatrix, coef=1)
#> [1] "For data.table input, assign columns using the 'peaknames' and 'intensities' arguments"
#>        MA_B6 Strain_w_B6 Strain_by_B6 CBS_Met
#> Down      18          11            8      36
#> NotSig   233         249          257     193
#> Up        23          14            9      45
```

The `eBayes` output can be obtained using the
`limma_out` argument.

```
DECu <- limmaTest(x=all.fq, peaknames="name", intensities=102:133, des=desCu, 
  cont=contMatrix, limma_out=TRUE)
#> [1] "For data.table input, assign columns using the 'peaknames' and 'intensities' arguments"
#>        MA_B6 Strain_w_B6 Strain_by_B6 CBS_Met
#> Down      18          11            8      36
#> NotSig   233         249          257     193
#> Up        23          14            9      45
```

The `topTable` columns appended to the input data and, in
particular, the `adj.P.Val` column is the main purpose of the
`limms` analysis. The rest of the vignette gives examples of
using those results in downstream analysis; however, given the
differential abundance analysis, several useful functions provided by
`limma` for quick quality control are highlighted.

A quick summary via `decideTests` is already printed as a
`limmaTest` output message, but can be regenerated.

Note: `decideTests` and the native `limma`
functions like `vennDiagram` and `qqt` only work
on an object of class `MArrayLM`, obtained through
`limmaTest` using the `limma_out=TRUE`
argument.

```
results <- decideTests(DECu)
summary(results)
#>        MA_B6 Strain_w_B6 Strain_by_B6 CBS_Met
#> Down      18          11            8      36
#> NotSig   233         249          257     193
#> Up        23          14            9      45
```

Venn diagrams

```
results2 <- results[, 1:2]
vennDiagram(results2)
```

QQ-plot of limma t-statistics

```
par(mfrow=c(2,2))
for(i in 1:4)
  {
    qqt(DECu$t[,i],df=DECu$df.residual+DECu$df.prior, 
        main=colnames(desCu)[i])
    abline(0,1)
  }
par(mfrow=c(1,1))
mtext("QQ-plots of limma t-statistics", line=3)
```

A volcano plot with more detail is shown later in the vignette, but a
quick version is a native `limma` option.

```
par(mfrow=c(2,2))
for(i in 1:4)
volcanoplot(DECu, coef=i, main=colnames(desCu)[i])
par(mfrow=c(1,1))
mtext("Volcano plots", line=3)
```

## 0.7 Database Matching

Now that compounds that vary significantly for a specified contrast
are identified, the function `dbMatch` can provide a first
approximation of the compound identities.

\*\* This is absolutely not a definitive identity\*\*, which would
require additional data.

Rather, `dbMatch` provides a list of matches based on
compounds with a similar mass. If multiple putative annotations are
found within the specified mass tolerance, 10 ppm by default, all are
listed.

Warning: because multiple putative identifications are allowed, the
output peak list may expand to larger than the input. When an input peak
has more than one putative match, that row is duplicated, but then
distinct annotations are appended for each match. Such duplicated rows
can occur for real biochemical reasons (e.g., leucine and isoleucine),
or technical artifacts (e.g., misshapen peaks), and/or in cases where
the database contains distinct annotations that overlap the mass window.
`dbMatch` cannot distinguish why a peak has more than one
match, although the annotations may help to determine the reason.

- A closer match is not validation of a particular identity.
- Annotated objects should be maintained separately from the input
  object to best manage the peaks that are duplicated by annotation.

The use of chromatographic information (retention time) can greatly
aid compound annotation. `dbMatch` includes arguments to
allow retention time matching to a database that includes
chromatographic information.

`dbMatch` requires that the user provide an appropriate
database.  
In this example, `HMDB_Shortlist` contains compounds used as
calibration standards in the CBS experiment. However, a larger list,
such as all metabolites found in a yeast cell, can be used. If retention
time matching is desired, the database must have the correct information
matching the chromatographic system used, which would generally be
laboratory-specific.

Warning! Do not publish annotations produced by `dbMatch`,
nor are they even suitable for public presentation. In our experience
with complex samples, more than half of the annotations are incorrect.
Intended uses for `dbMatch` include:

- prioritizing the peaks to validate with standards or CID-MS in
  further experiments.
- filtering contaminant peaks [by their lack of annotation as organic
  compounds]
- filtering isotopes, alternative adducts, or multimers
- investigating mass/retention time clusters or class enrichment
- aligning new data to historical data.

```
# Read in a list of knowns. hmdb_Shortlist contains a list
# of compounds used as calibration standards in the CBS experiment.
data(hmdb_Shortlist)
hmdb <- hmdb_Shortlist
```

Run `dbMatch`. The `mzmed` column in the
`CBS.xcms_diffreport` is column 6. The
`db_annotations` argument passes annotation information to
the output object.

```
x.ID_db <- dbMatch(x=CBS.xcms_diffreport, peaknames="name", mzmedcol="mzmed", 
  db=hmdb, dbcol="Adduc_MW_.Da.", ppmCut=10, db_annotations=c("ID", 
  "Formula", "HMDB_ID", "Adduct"))
#> [1] "No retention time detected.  Mass match only"

# Both the peaklist and database have an "ID" column, 
# so the merge failed with an error message.

# Change the name of the database column and re-run
names(hmdb)[1] <- "Metabolite"

x.ID_db <- dbMatch(x=CBS.xcms_diffreport, peaknames="name", mzmedcol="mzmed", db=hmdb, 
  dbcol="Adduc_MW_.Da.", ppmCut=10, 
  db_annotations=c("Metabolite", "Formula", "HMDB_ID", "Adduct"))
#> [1] "No retention time detected.  Mass match only"
```

`dbMatch` can be used on other input types, like a
`limma` topTable object so that only significant hits are
annotated. Read in a `topTable`, such as the significant
metabolites from the CBS data ranked by F-test in the dataset
`ttF.CBS`, merge with the mass info, keeping the
`topTable` order, and annotate.

```
ttF_anno <- merge(data.table(ttF.CBS), CBS.xcms_diffreport, by.x="ID", by.y="name", 
                  all.x=TRUE, all.y=FALSE, sort=FALSE)

x.ID_db <- dbMatch(x=ttF_anno, peaknames="ID", mzmedcol="mzmed", db=hmdb, 
  dbcol=4, ppmCut=10, 
  db_annotations=c("Metabolite", "Formula", "HMDB_ID", "Adduct"))
#> [1] "No retention time detected.  Mass match only"

# A fast check: pull only the database hits
# Now with a proper adjusted p-value
knitr::kable(x.ID_db[!is.na(exact_mz), c(1, 9, 14, 78, 81)], 
             caption="CBS peaks matching standards")
```

CBS peaks matching standards


| ID | adj.P.Val | mzmed | exact\_mz | Formula |
| --- | --- | --- | --- | --- |
| M116T279 | 0.0081598 | 116.0708 | 116.0706 | C5H9NO2 |
| M118T468 | 0.0000000 | 118.0863 | 118.0863 | C5H11NO2 |
| M120T220 | 0.0000003 | 120.0657 | 120.0655 | C4H9NO3 |
| M132T949 | 0.0000000 | 132.1021 | 132.1019 | C6H13NO2 |
| M147T264 | 0.0000000 | 147.1137 | 147.1128 | C6H14N2O2 |
| M150T549 | 0.0000000 | 150.0589 | 150.0583 | C5H11NO2S |
| M156T552 | 0.0001063 | 156.0725 | 156.0731 | (13)CH3S(13CH2)2(13)CH(15)NH2(13)CO2H |
| M166T1042 | 0.0000000 | 166.0865 | 166.0863 | C9H11NO2 |
| M170T976 | 0.0000202 | 170.0815 | 170.0812 | C8H11NO3 |
| M175T306 | 0.0000023 | 175.1202 | 175.1190 | C6H14N4O2 |
| M182T910 | 0.0000000 | 182.0820 | 182.0812 | C9H11NO3 |
| M205T1285 | 0.0002016 | 205.0974 | 205.0972 | C11H12N2O2 |
| M269T446 | 0.0000000 | 269.0639 | 269.0624 | C8H16N2O4S2 |
| M298T1133 | 0.0000000 | 298.0990 | 298.0968 | C11H15N5O3S |
| M308T503 | 0.0000000 | 308.0929 | 308.0911 | C10H17N3O6S |
| M385T1001 | 0.0000709 | 385.1303 | 385.1288 | C14H20N6O5S |
| M400T665 | 0.0000001 | 400.1492 | 400.1523 | C15H23N6O5S |

## 0.8 Data Visualizations

`limms` objects can be sliced to a shortlist of
statistically significant compounds to use for data visualizations.
There are many possible methods, but as for transcriptomics analyses,
dot plots, volcano plots and heatmaps are useful.

Looking at the changes for the levels of one compound, stratified by
covariates, is a good way to detect patterns.

```
# pull methionine
methionine <- melt(tT.F[name %in% "M269T446"], measure.vars=patterns("norm"), 
  variable.name="sample", value.name="intensity")[, c(1,109:111)]

# append the B6 supplementation information
methionine[, B6 := desMetB6$B6]
methionine[, allele := desMetB6$Strain]

# Add identifying colors
methionine[, colorCBS_B6 := c("red", "darkorange", "violet", "blue")[as.factor(paste(
  desMetB6$Strain, desMetB6$B6, sep="_"))]]

ggplot(methionine, aes(x=B6, y=intensity, color=colorCBS_B6)) +
  geom_jitter(width=0.3) +
  stat_summary(fun="median", size=18, geom="point", shape="-") +
  scale_color_identity() +
  facet_wrap(~allele) +
  ylim(0, 24) +
  theme_bw() +
  ggtitle("methionine intensity with B6 supplementation by CBS allele")
```

A nicer volcano plot using `ggplot`

```
# pull the topTable for contrast 1, B6 supplementation of the major allele only:
tt1.4volcano <- melt(tt1, measure.vars=patterns("norm"), variable.name="sample", 
  value.name="intensity")[, c(1, 102, 106)]


ggplot(tt1.4volcano, aes(x=logFC, y=-log10(adj.P.Val))) +
    geom_point(shape=1, size=2) +
  theme_bw() +
    geom_hline(yintercept=1.30103, linetype="dotted") +
    geom_vline(xintercept=1, linetype="dotted") +
    geom_vline(xintercept=-1, linetype="dotted") +
  ggtitle("p-value by log-fold-change\nmetabolites changed by B6 supplementation\n
    for the major CBS allele")
```

A heatmap of the most significant hits in the vitamin B6
contrast.

```
# apply p-value and log-fold-change cutoffs

tt1.pCut <- limmaTest(x=all.fq, peaknames="name", intensities=102:133, des=desCu, 
  cont=contMatrix, coef=1, p.value=0.01, lfc=2)
#> [1] "For data.table input, assign columns using the 'peaknames' and 'intensities' arguments"
#>        MA_B6 Strain_w_B6 Strain_by_B6 CBS_Met
#> Down      18          11            8      36
#> NotSig   233         249          257     193
#> Up        23          14            9      45
# down to 26 metabolites, a reasonable number for a heatmap

tt1.scaled <- data.table(reshape2::melt(tt1.pCut[, .(name, .SD), .SDcols=patterns("norm")],
  id.vars=1, variable.name="sample", value.name="intensity"))
tt1.scaled[, scaled := scale(.SD), by=name, .SDcols="intensity"]

# make the heatmap
hm.tt1 <- ggplot(tt1.scaled, aes(x=fct_inorder(sample), y=name, fill=scaled)) +
  geom_raster() +
  scale_fill_gradient2(low="blue", mid="white", high="red") +
  scale_y_discrete(position="right", limits=rev(levels(tt1.scaled$name))) +
  coord_cartesian(expand=FALSE) +  
  theme(axis.text.x=element_blank(), axis.title.x=element_blank(), legend.position="left",
        legend.text.align=0.5, legend.margin=margin(0,0,0,0, unit="pt"), 
        legend.key.size=unit(0.4, "cm"), legend.title.align=0.5) +
  guides(fill="colorbar") +
  labs(fill="intensity\nZ-score") +
    ylab("metabolite, n = 25\n") +
  ggtitle("heatmap of metabolites\nF-test p < 0.01 and log2-fold-change > 2")


# map classes onto the samples
desMetB6.melt <- melt(data.table(desMetB6), id.vars="Run", variable.name="covariate", 
  value.name="class")

# make a covariates table
hm.classes <- ggplot(desMetB6.melt, aes(x=fct_inorder(Run), y=covariate, fill=class)) +
  geom_raster() +
  geom_text(aes(label=class), angle=90) +
  coord_cartesian(expand=FALSE) +  
  theme(axis.text.x=element_blank(), legend.position="none", legend.text.align=0.5, legend.margin=margin(0,0,0,0, unit="pt"), legend.key.size=unit(0.4, "cm"), 
        legend.title.align=0.5) +
  xlab("mass spectrometry sample")

# put the heatmap and table together
plot_grid(hm.tt1, hm.classes, nrow=2, rel_heights=c(0.66, 0.34), axis="lr", align="v")
```

## 0.9 Version Info

```
sessionInfo(package="limms")[5]
#> $tzcode_type
#> [1] "internal"
```
