# Supplementary material for: Mycobacteria that cause tuberculosis have retained ancestrally acquired genes for the biosynthesis of chemically diverse terpene nucleosides
Source: PLoS Biol. 2024 Sep 30;22(9):e3002813. doi: 10.1371/journal.pbio.3002813 (PMC11476799; doi:10.1371/journal.pbio.3002813)
Supplement: S4 Data — (PDF) [file pbio.3002813.s016.pdf]

The synthesis of 1-tuberculosinyl adenine and N^6-tuberculosinyl adenine commenced with the protection of N9 in adenine with a trityl group (Scheme 1).[1] The next step was to find suitable conditions for N1 alkylation. Previously, our group has demonstrated that N1 alkylation of adenosine can be achieved with a series of allylic chlorides, assisted by addition of sodium iodide in DMF.[2] Alkylation of trityl-adenine **1** with freshly prepared tuberculosinyl chloride according to this procedure did not lead to complete conversion in 12 h and we observed considerable precipitation of salts. This issue was solved by adding a small amount of dichloromethane. This provided **2** in 65% yield after column purification. Part of the material was subsequently subjected to Dimroth rearrangement using Me_2NH (40% solution in water). This to prepare N^6-tuberculosinyl adenine. Trityl protected 1-tuberculosinyl adenine **2** has characteristic chemical shifts: δ 8.41, and 7.85 ppm whereas N^6-tuberculosinyl-adenine **3** has characteristic chemical shifts δ 7.53, and 7.39 ppm.

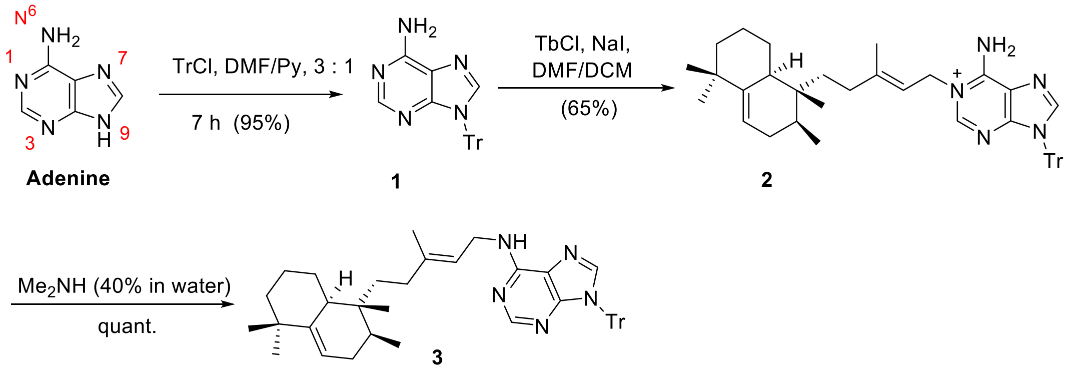

__Scheme 1__. Synthesis of N9-trityl protected tuberculosinyl-adenines.

To obtain the final compounds, the trityl groups in **2** and **3** had to be removed and the compounds were therefore treated with a mixture of TFA/CH_2Cl_2 at rt. Although this led to the formation of products having the appropriate molecular mass (408 amu), ^1H NMR showed that double bond migration in the tuberculosinyl unit had occurred leading to disappearance of an “alkene hydrogen”. These compounds were, after close examination, assigned as **4** and **5** (Scheme 2).

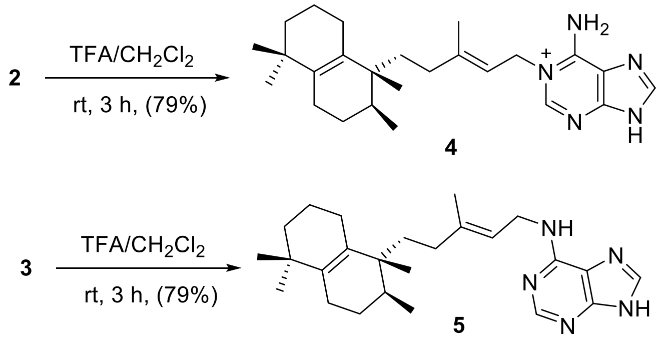

Scheme 2. TFA-mediated removal of the trityl protecting groups in **2** and **3** leads to Wagner-Meerwein shifts

The problem of the undesired rearrangement was solved by using formic acid as a weaker acid in the deprotection step (**Scheme 3**). The compounds **2**, and **3** were treated with a solution of formic acid in chloroform at -10 °C. These reactions provided the desired products in good yields. The NMR spectra of **6** and **7** are very similar and in order to exclude potential Dimroth rearrangement of **2** or **6** during the deprotection reaction, the compounds were subjected to UPLC (Acquity UPLC BEH C8-Column, 1.7 μm, flow rate 0.3 mL/min, 0.01% TFA in CH_3CN, water: 5% to 50%, run time 17 min). The chromatogram showed retention times of 12.4 and 11.8 min for **6** and **7**, respectively. These similar but not identical

retention times led to the conclusion that **6** is a very weak base and preferentially exists in its "deprotonated" neutral form (**6'**). This was confirmed by treating **6'** and **7** with HCl in methanol which led to the formation of the charged species **6** and **7'**. Also in this case, the ^{1}H-NMR spectra of these compounds were very similar.

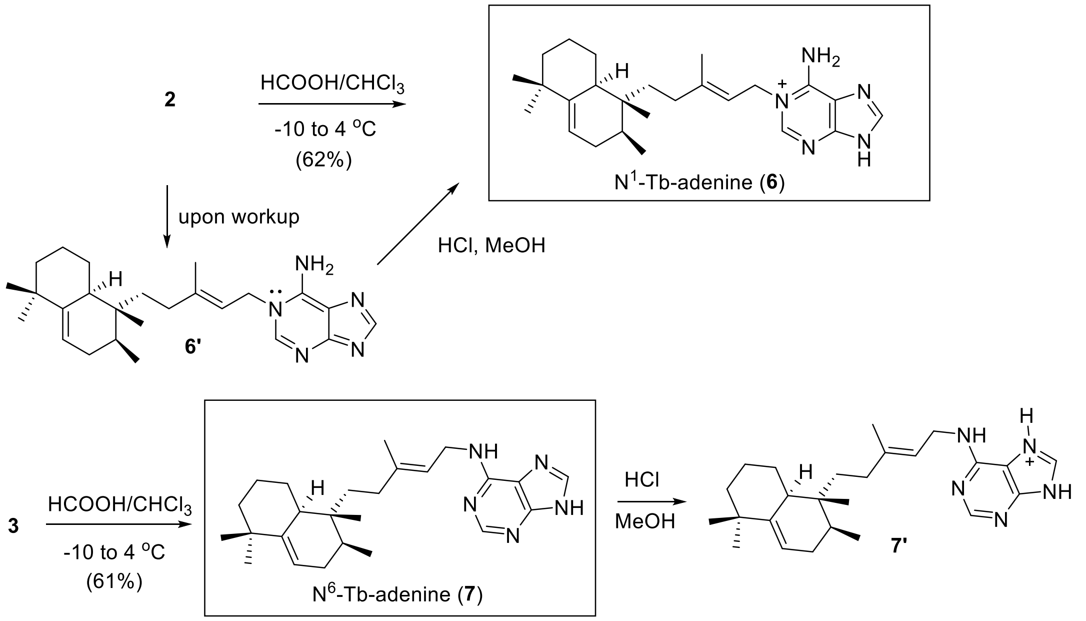

***Scheme 3**. Trityl deprotection and proton shift to form Tb-adenines most substituted Tb-adenines.*

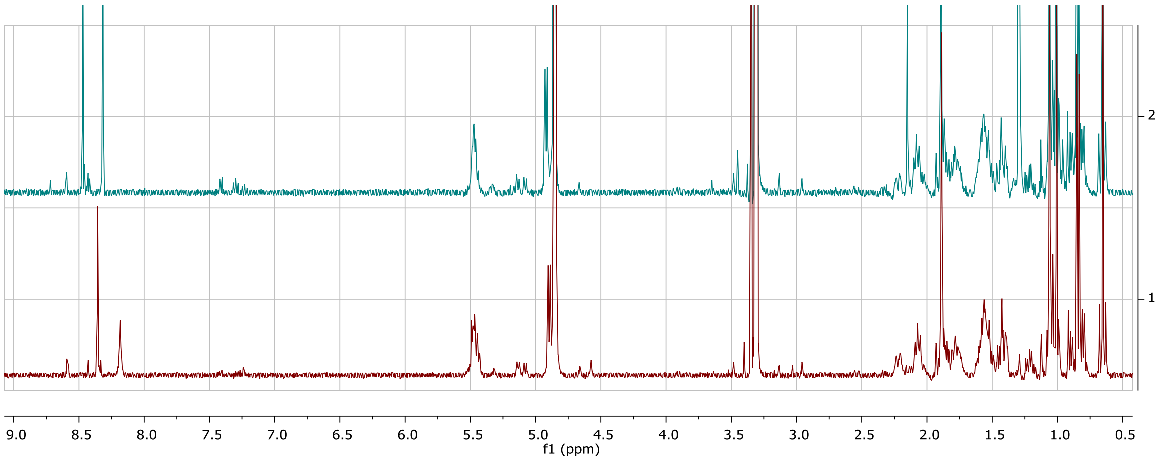

***Figure 1.** ^{1}H NMR spectra of 6 (top) and 6' (bottom) in methanol-d4.*

9-trityl-9H-purin-6-amine (1)[1]

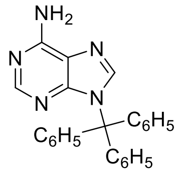

To a stirred solution of adenine (3.23 g, 23.9 mmol) in DMF (24 mL) was added sequentially pyridine (72 mL) and Ph_3CCl (6.66 g, 23.9 mmol, 1.0 eq) at rt. The reaction was allowed to stir at rt overnight. The reaction mixture was diluted with EtOAc (150 mL) and water (100 mL). The layers were separated and the organic layer was washed with water (4 x 100 mL), dried with anhydrous $MgSO_4$ and concentrated under reduced pressure to give the crude as a solid in oil suspension. This was re-suspended in Et_2O and filtered. The solid (2.0 g) was dried and characterized by NMR, which showed pure **1**. The filtrate was concentrated and purified by flash column chromatography (EtOAc/pentane = 2/8 to MeOH/DCM = 1/9) to give a second batch of the pure product as a colorless solid (3 g). ^{1}H NMR (400 MHz, Chloroform-*d*) δ 8.05 (s, 1H), 7.75 (s, 1H), 7.36 – 7.27 (m, 10H), 7.21 – 7.11 (m, 5H), 5.99 (br, 2H); ^{13}C NMR (101 MHz, Chloroform-*d*) δ 152.5, 151.5, 142.0, 142.0, 141.5, 129.9, 128.2, 128.0, 121.3, 75.9. The spectral data were identical with those in literature.

6-amino-1-((E)-3-methyl-5-((1R,2S,8aS)-1,2,5,5-tetramethyl-1,2,3,5,6,7,8,8a-octahydronaphthalen-1-yl)pent-2-en-1-yl)-9-trityl-9H-purin-1-ium (2)

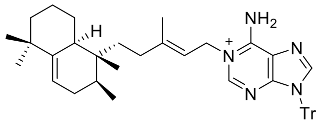

To a suspension of N-chloro succinimide (0.088 g, 1.3 equiv) in dry CH_2Cl_2 (1.3 mL) at −20 °C, was added dropwise dimethyl sulfide (0.057 mL, 1.5 equiv) in dry CH_2Cl_2 (0.44 mL). After addition, the reaction became a milky suspension, which was allowed to warm to 0 °C for 15 min, whereafter the temperature was lowered to – 40 °C and tuberculosinol[3] (0.150 g, 0.52 mmol) in dry CH_2Cl_2 (1.7 mL) was added dropwise over 15 min. After addition, the cooling bath was removed, and the reaction was allowed to warm up to rt. The reaction was allowed to stir at this temperature for 2 h, after which TLC indicated complete conversion of the tuberculosinol. The reaction mixture was concentrated under reduced pressure and treated with pentane upon which succinimide oiled out. The mixture was decanted and filtered, and the filtrate was concentrated under reduced pressure affording nearly pure tuberculosinyl chloride (0.135 g) as a yellow oil.

The obtained tuberculosinyl chloride (0.135 g, 0.47 mmol) was dissolved in DMF and NaI (0.085 g, 0.56 mmol, 1.2 equiv) and trityl adenine (0.193 g, 0.51 mmol, 1.1 equiv) were added at rt. The reaction flask was covered with aluminum foil and allowed to stir at rt for 16 h. The conversion was monitored by TLC, which indicated 50% conversion. The reaction mixture was viscous and contained a lot of solid material. Therefore, CH_2Cl_2 (1 mL) was added and the reaction mixture was stirred for an additional 11 h. Volatiles were removed in vacuo and the crude was purified by column chromatography (EtOAc/pentane = 2/1) to give **2** (0.208 g, 65%) as a brown amorphous solid. ^{1}H NMR (400 MHz, Chloroform-*d*) δ 8.41 (s, 1H), 7.85 (s, 1H), 7.40 – 7.27 (m, 9H), 7.21 – 7.11 (m, 6H), 5.46 – 5.34 (m, 2H), 5.09 (d, *J* = 7.1 Hz, 2H), 2.15 – 5.01 (m, 2H), 2.09 (d, *J* = 13.2 Hz, 1H), 2.01 – 1.92 (m, 2H), 1.89 - 1.84 (m, 2H), 1.78 (s, 3H), 1.75 – 1.64 (m, 2H), 1.60 – 1.50 (m, 2H), 1.49 – 1.38 (m, 3H), 1.38 – 1.23 (m, 2H), 1.04 (s, 3H), 0.99 (s, 3H), 0.77 (d, *J* = 6.7 Hz, 3H), 0.61 (s, 3H). ^{13}C NMR (101 MHz, cdcl$_3$) δ 162.7, 150.9, 146.0, 143.2, 140.4, 129.9, 129.7, 128.7, 128.5, 128.2, 128.0, 116.3, 113.0, 41.0, 39.9, 37.1, 36.6, 36.2, 34.6, 33.5, 33.1, 31.7, 31.6, 29.9, 29.1, 27.6, 22.3, 17.9, 16.3, 15.4, 15.3. MS: (ESI$^+$) calculated mass [M]$^+$ $C_{44}H_{52}N_5^+$ = 650.42, found: 650.71.

Trityl deprotection:

A solution of N^9-tritylated substrate (10 mg, 0.02 M) in $CHCl_3$ was cooled to -10 °C by an ice-salt bath before MeOH (0.01 mL) and a mixture (0.2 mL, 1:1) of formic acid in chloroform was added. The reaction was allowed to reach 5 °C in 2.5 h. Thereafter, the reaction was quenched with saturated aqueous $NaHCO_3$ (3 mL) and diluted with $CHCl_3$ (2 mL). This mixture was stirred for 10 min before layers

were separated and the aqueous layer was extracted with chloroform (5 mL). The combined organic extract was dried with $MgSO_4$, and concentrated *in vacuo* to give the crude product as a brown solid. This product was triturated with pentane (3 x 2 mL) to remove the residual trityl formate, leaving behind a solid, which was washed with Et_2O (1.5 mL) to give the pure product as a colorless solid.

6-amino-1-((E)-3-methyl-5-((1R,2S,8aS)-1,2,5,5-tetramethyl-1,2,3,5,6,7,8,8a-octahydronaphthalen-1-yl)pent-2-en-1-yl)-9H-purin-1-ium (6')

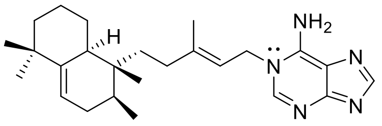

Compound **2** (10 mg, 0.015 mmol) was treated according to the procedure to afford **3** (4 mg, 62%) as a yellow solid. ^{1}H NMR (600 MHz, methanol-d_4) δ 8.25 (s, 1H), 8.07 (s, 1H), 5.41 – 5.28 (m, 2H), 4.80 (d, *J* = 6.7 Hz, 2H), 2.14 – 2.09 (m, 1H), 1.97 (dt, *J* = 10.7, 5.1 Hz, 2H), 1.79 (d, *J* = 1.2 Hz, 3H), 1.75 (dt, *J* = 5.4, 2.6 Hz, 1H), 1.71 – 1.63 (m, 2H), 1.53 – 1.39 (m, 4H), 1.35 – 1.27 (m, 2H), 1.25 – 1.17 (m, 2H), 1.11 (td, *J* = 12.8, 4.9 Hz, 1H), 0.96 (m, 3H), 0.91 (s, 3H), 0.74 (d, *J* = 6.8 Hz, 3H), 0.55 (s, 3H). ^{13}C NMR (151 MHz, Methanol-d_4) δ 147.2, 147.1, 145.5, 117.5, 116.2, 116.1, 49.6, 42.1, 41.2, 38.1, 37.0, 36.0, 34.6, 33.9, 32.7, 30.3, 29.5, 28.6, 23.2, 17.0, 16.6, 15.5. HRMS: (ESI$^+$) calculated mass $[M]^+$ $C_{25}H_{38}N_5^+$ = 408.312, found: 408.311.

N-((E)-3-methyl-5-((1R,2S,8aS)-1,2,5,5-tetramethyl-1,2,3,5,6,7,8,8a octahydronaphthalen-1-yl)pent-2-en-1-yl)-9-trityl-9H-purin-6-amine (3)

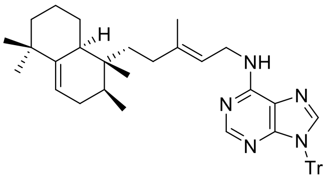

To a solution of **2** (50 mg, 0.07 mmol) in THF (1 mL) was added Me_2NH (2.0 mL, 40% solution in water) and the resulting solution was allowed to stir for 2.5 h. TLC indicated complete conversion of the starting material. The volatiles were removed by distillation *in vacuo* to give the crude product (45 mg, 95%) as a white solid. The crude compound was found sufficiently pure by NMR analysis. Therefore, we proceeded to the next step without further purification. ^{1}H NMR (400 MHz, Chloroform-*d*) δ 7.53 (s, 1H), 7.39 (s, 1H), 7.34 – 7.28 (m, 9H), 7.18 – 7.11 (m, 6H), 5.42 (dt, *J* = 4.8, 2.2 Hz, 1H), 5.36 (tt, *J* = 5.8, 3.4 Hz, 1H), 4.58 (d, *J* = 7.2 Hz, 2H), 2.12 (d, *J* = 12.9 Hz, 1H), 1.93 (h, *J* = 5.7 Hz, 2H), 1.87 – 1.82 (m, 2H), 1.73 (d, *J* = 1.3 Hz, 3H), 1.70 (s, 1H), 1.56 (tt, *J* = 7.1, 3.6 Hz, 2H), 1.52 – 1.39 (m, 4H), 1.20 (dp, *J* = 12.9, 4.4 Hz, 2H), 1.05 (s, 3H), 0.99 (s, 3H), 0.78 (d, *J* = 6.8 Hz, 3H), 0.60 (s, 3H).

N-((E)-3-methyl-5-((1R,2S,8aS)-1,2,5,5-tetramethyl-1,2,3,5,6,7,8,8a-octahydronaphthalen-1-yl) pent-2-en-1-yl)-9H-purin-6-amine (7)

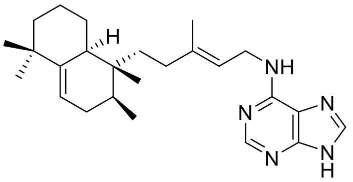

compound **3** (13 mg, 0.020 mmol) was deprotected according to the general procedure to afford **7** (5 mg, 61%) as a colorless solid. ^{1}H NMR (600 MHz, methanol-d_4) δ 8.35 (s, 1H), 8.19 (s, 1H), 5.53 – 5.36 (m, 2H), 4.90 (d, *J* = 6.7 Hz, 2H), 2.22 (dtd, *J* = 13.1, 4.1, 2.1 Hz, 1H), 2.07 (dq, *J* = 10.8, 6.0, 4.9 Hz, 2H), 1.91 – 1.88 (m, 3H), 1.88 – 1.82 (m, 1H), 1.81 – 1.74 (m, 2H), 1.55 (m, 4H), 1.45 – 1.38 (m, 2H), 1.20 (td, *J* = 12.8, 4.9 Hz, 1H), 1.06 (s, 3H), 1.01 (s, 3H), 1.05 – 1.01 (m, 2H), 0.84 (d, *J* = 6.8 Hz, 3H), 0.65 (s, 3H). ^{13}C NMR (151 MHz, Methanol-d_4) δ 150.9, 149.1, 147.2, 147.1, 145.5, 143.7, 117.5, 116.2, 42.1, 41.2, 40.4, 38.1, 37.0, 36.0, 34.6, 33.9, 32.7, 30.3, 29.5, 28.6, 23.2, 17.0, 16.6, 15.5. HRMS: (ESI$^+$) calculated mass $[M + H]^+$ $C_{25}H_{38}N_5^+$ = 408.312, found: 408.312.

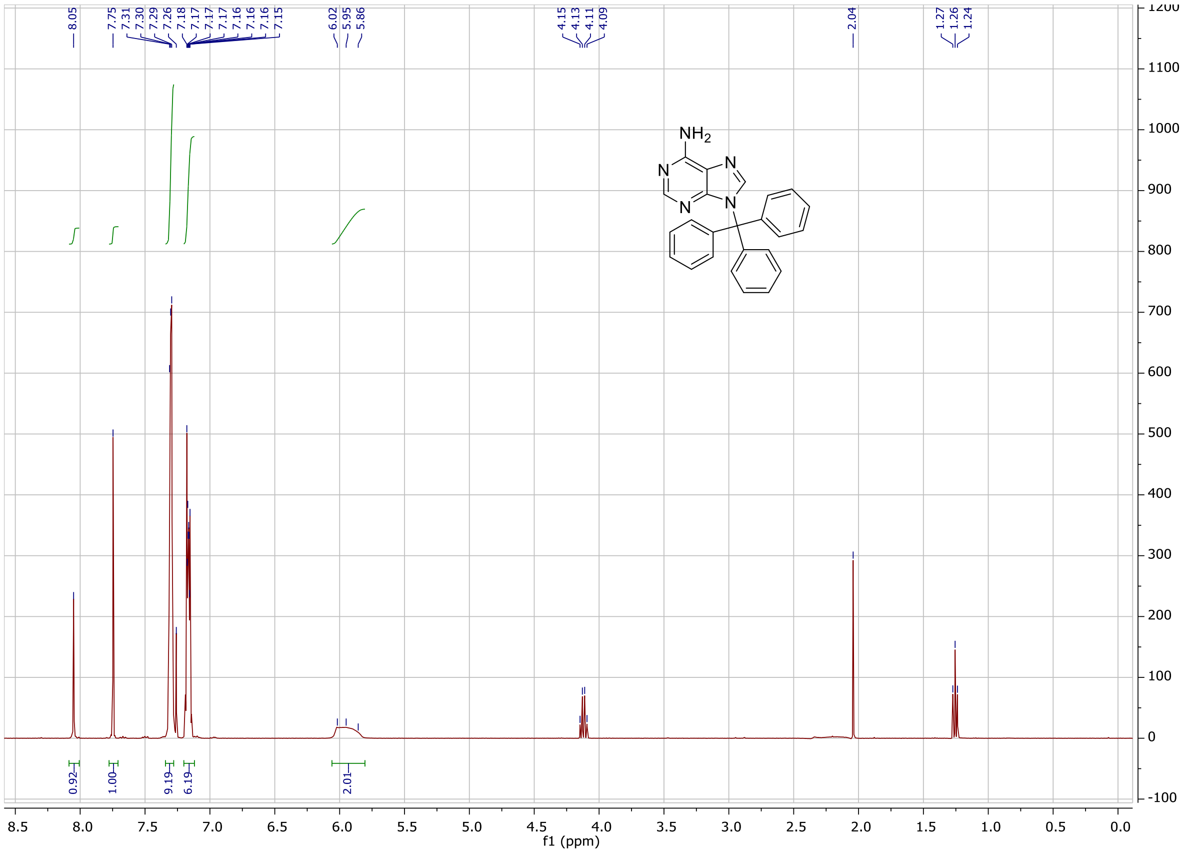

^{1}H NMR (400 MHz) spectrum of 9-Trityl adenine (**1**) in Chloroform-*d*.

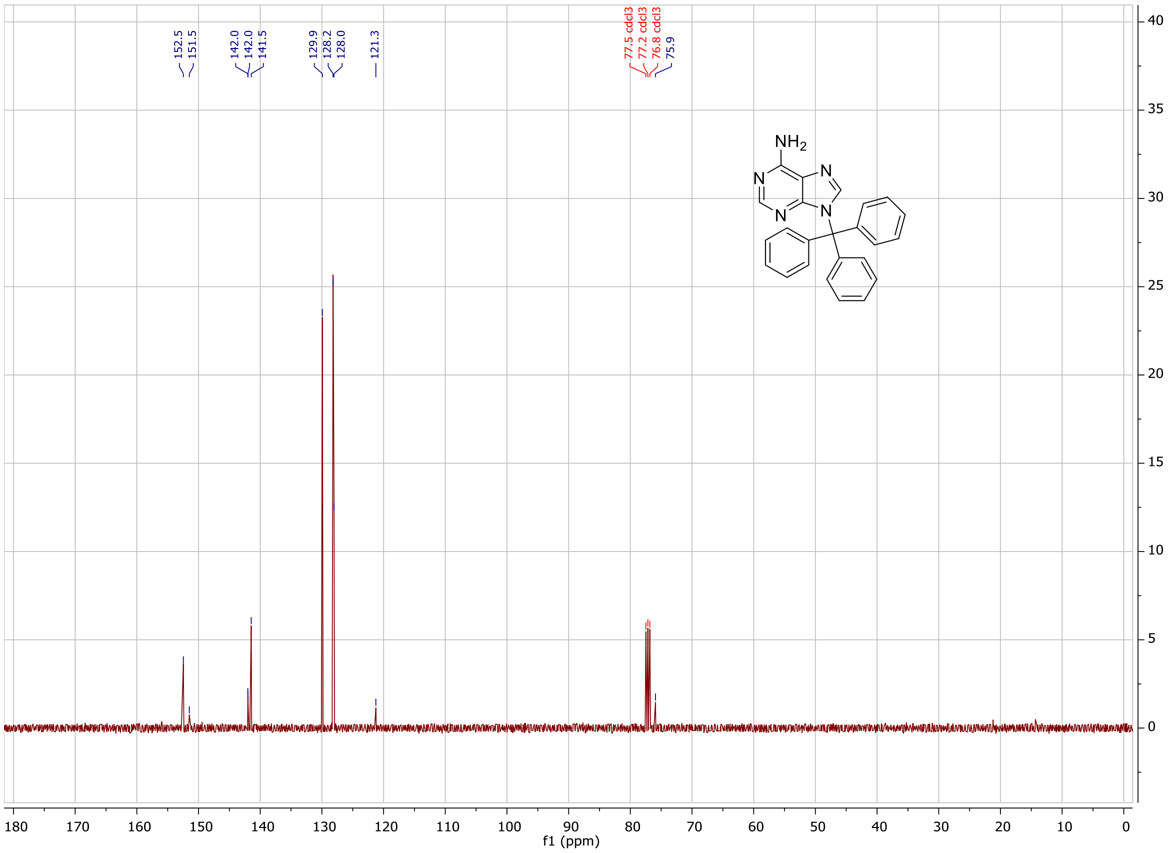

^{13}C NMR (100 MHz) spectrum of *N*9-trityl adenine (**1**) in Chloroform-*d*.

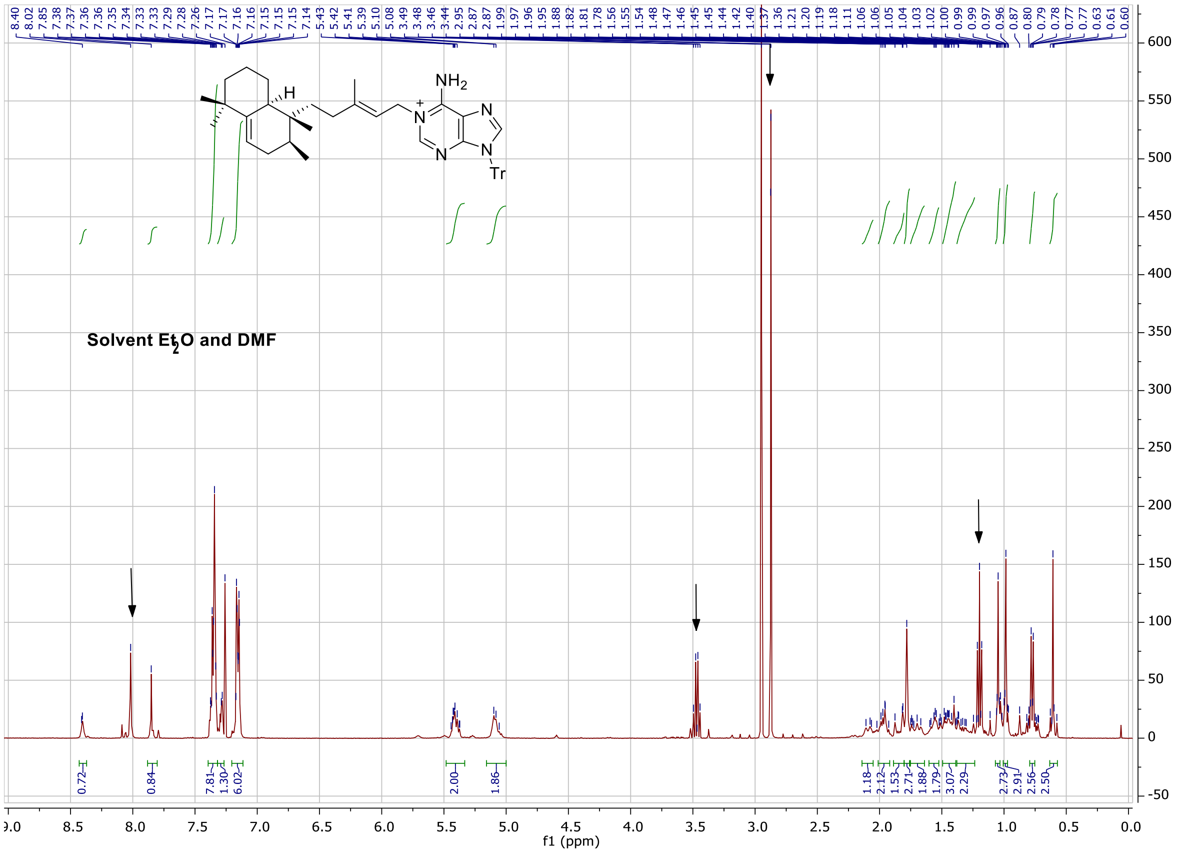

^{1}H NMR (400 MHz) spectrum of *N*9-trityl Tb-adenine **3** in Chloroform-*d*.

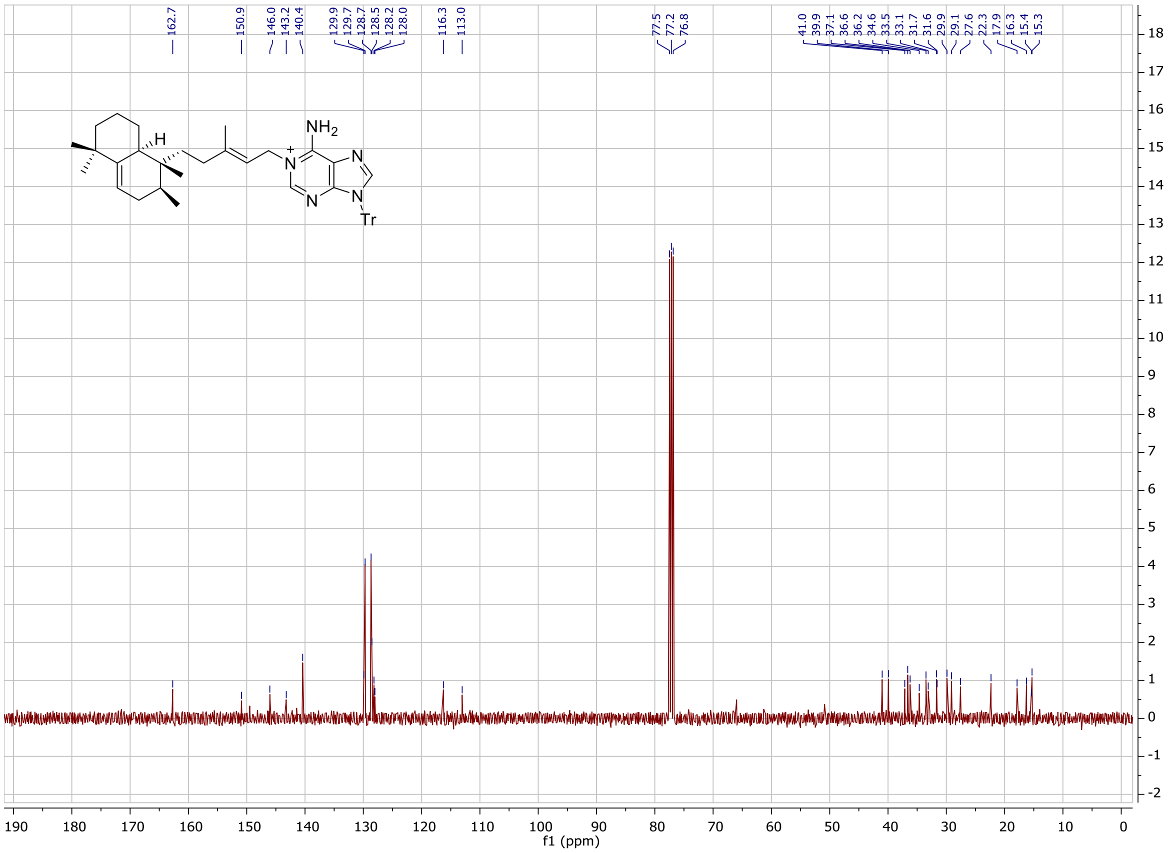

^{1}H NMR (400 MHz) spectrum of *N*9-trityl Tb-adenine **3** in Chloroform-*d*.

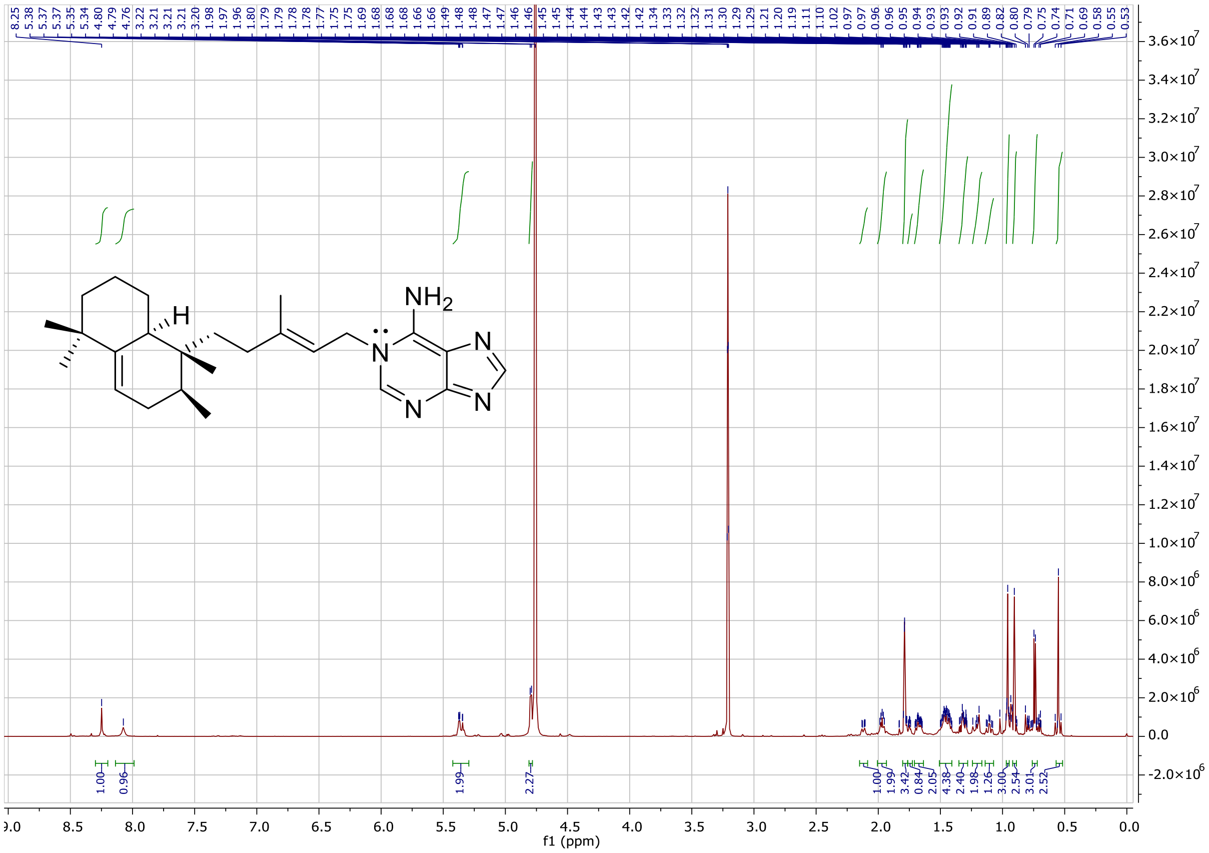

^{1}H NMR (600 MHz) spectrum of N^{1} Tb-adenine **6'** in methanol-d_4.

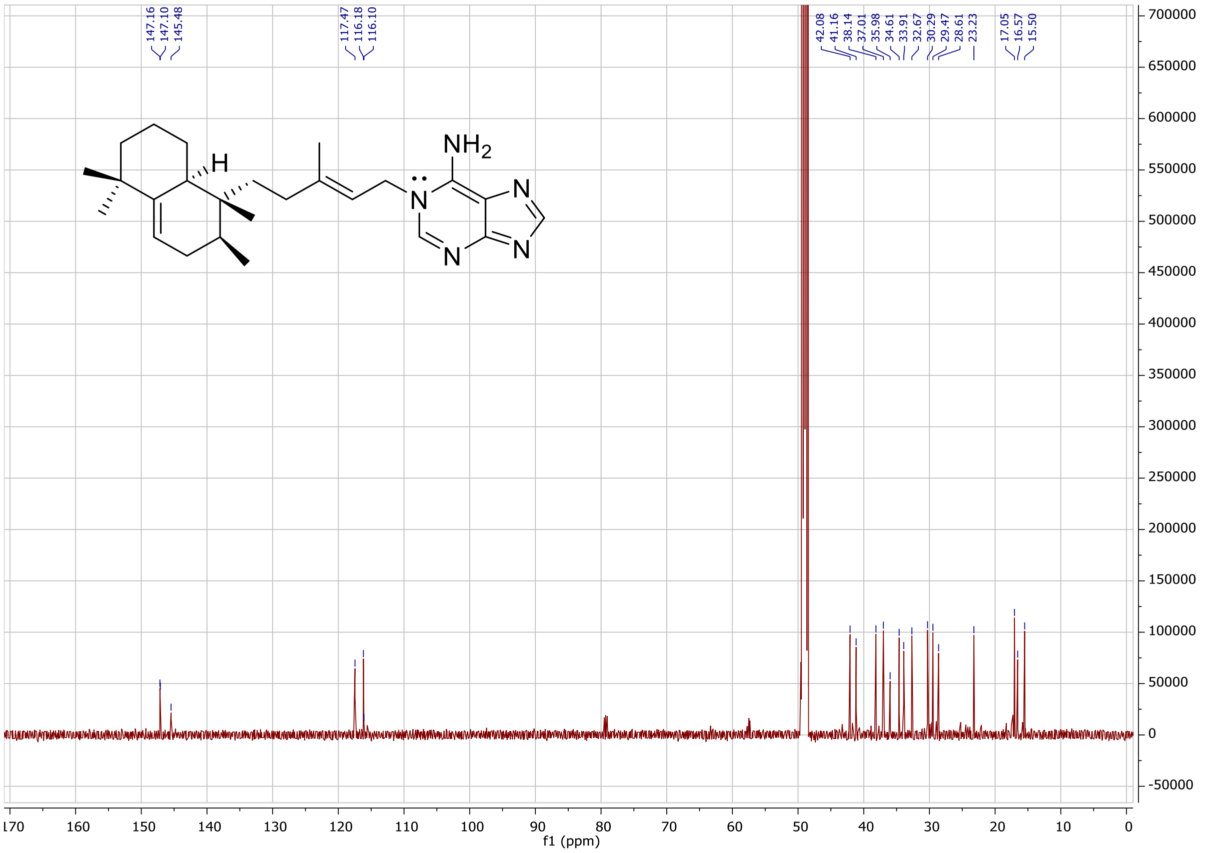

^{1}H NMR (600 MHz) spectrum of N^{1} Tb-adenine **6'** in methanol-d_4.

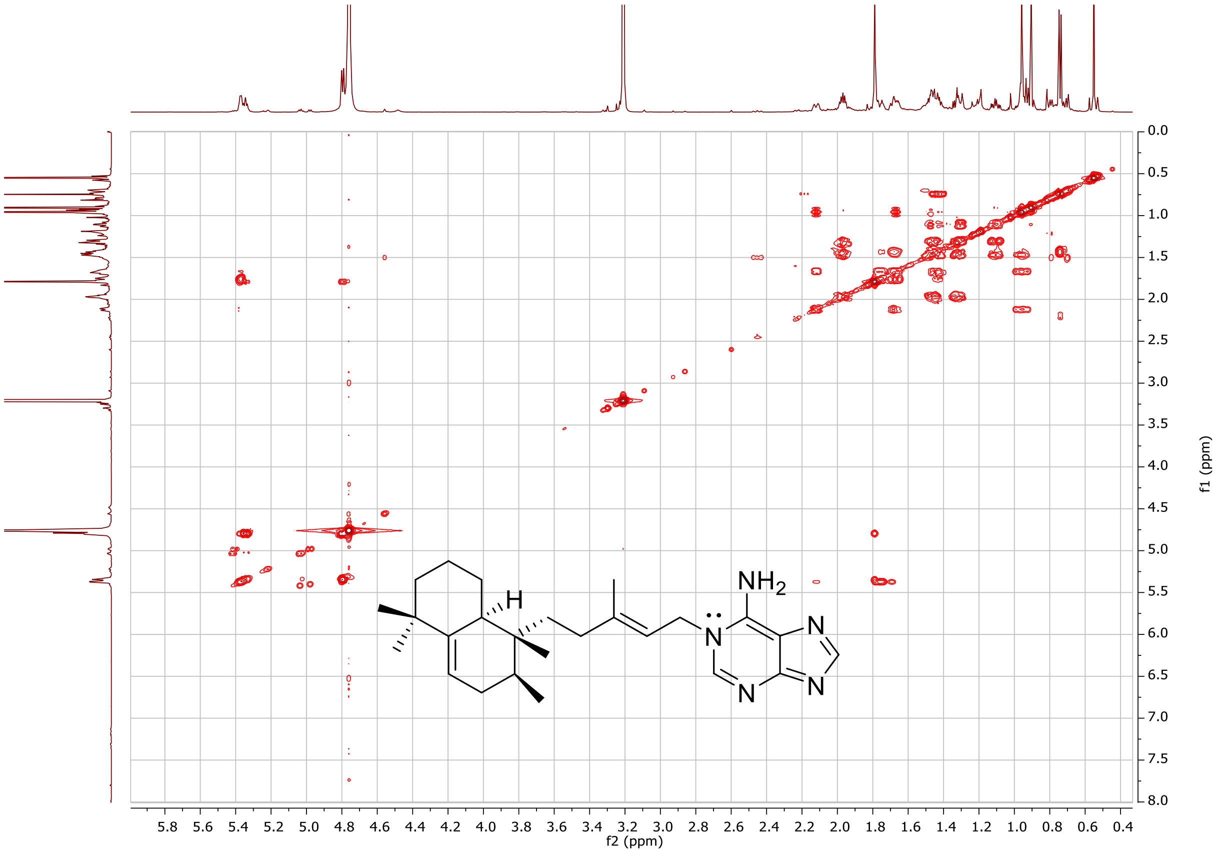

COSY expansion spectrum of N[1] Tb-adenine **6’** in methanol-d_4.

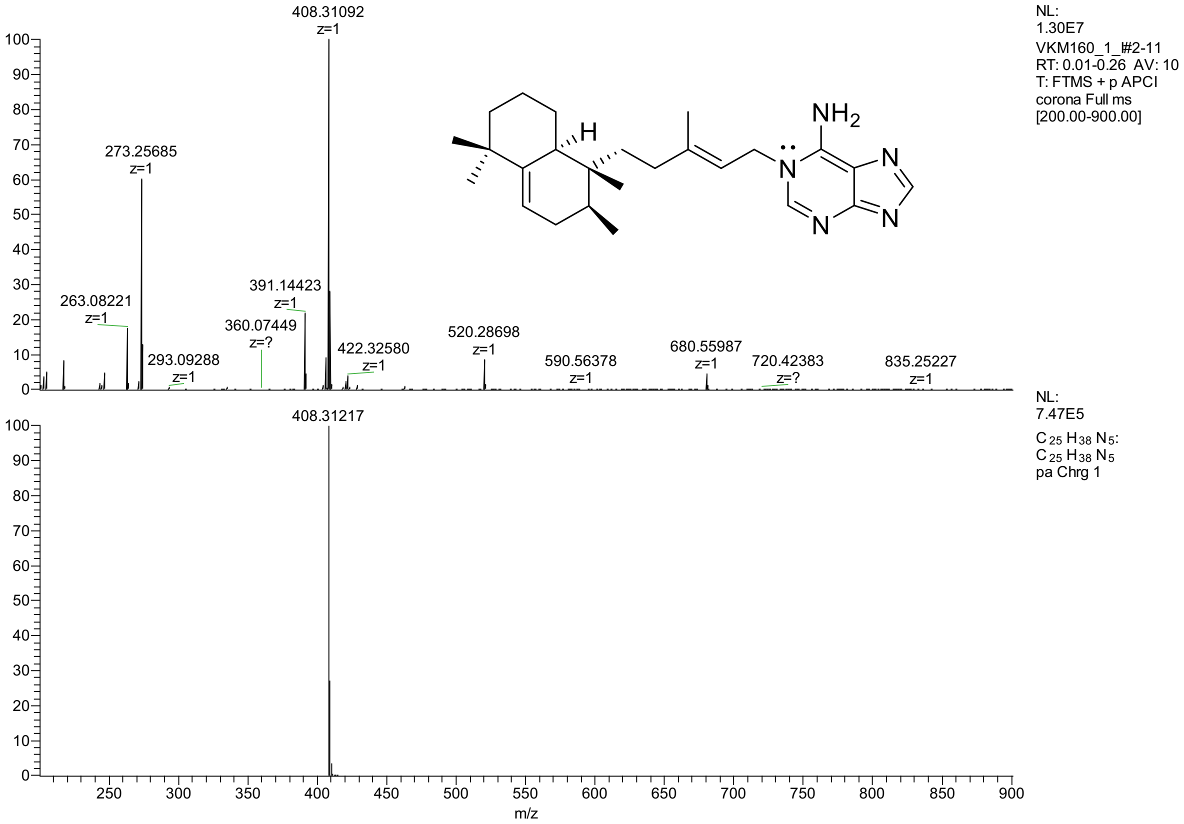

ESI-MS spectrum of N[1]-Tb-adenine **6’**.

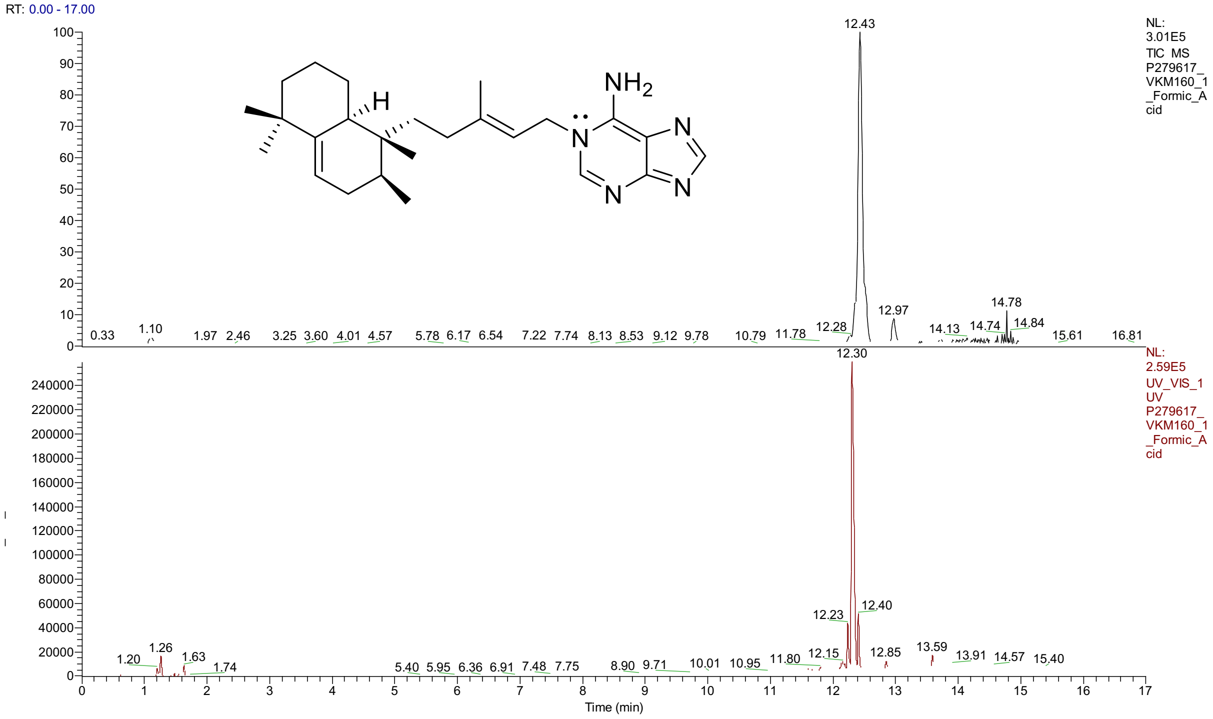

UPLC Chromatogram of N[1]-Tb-adenine.

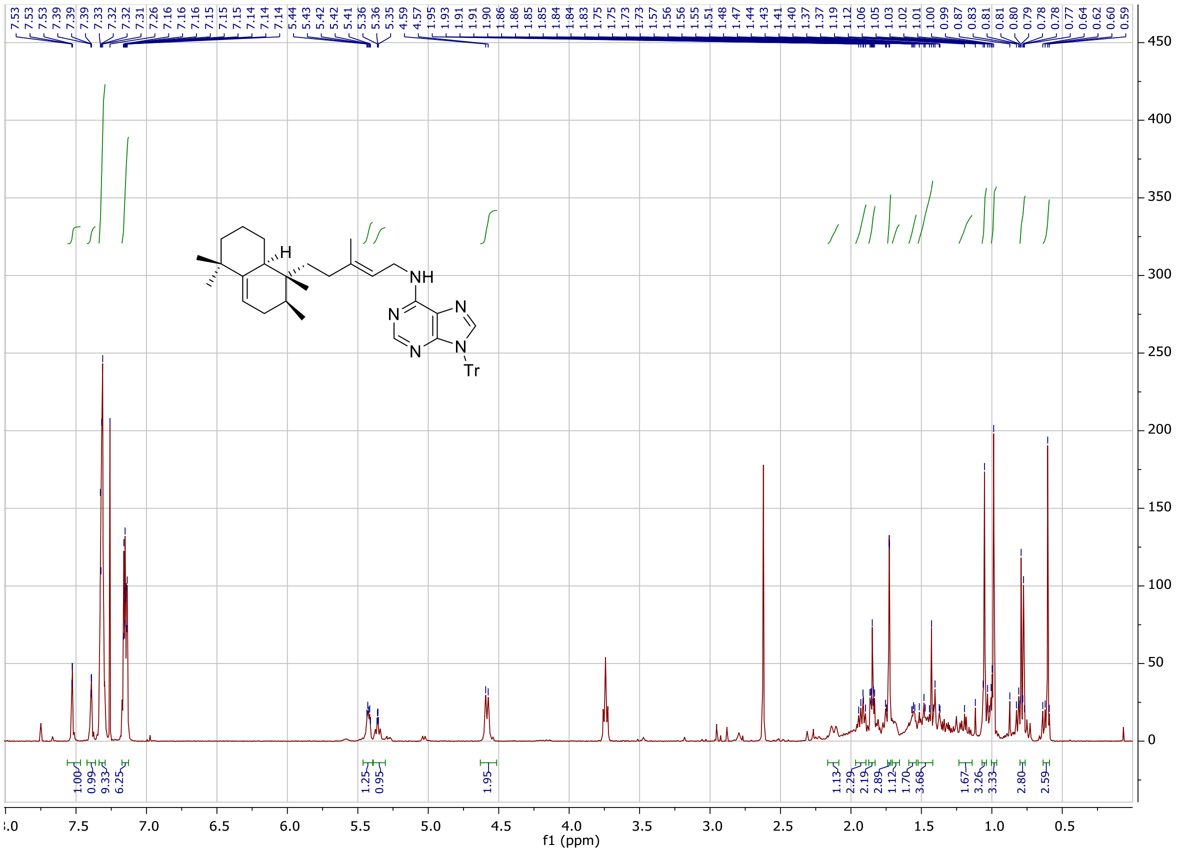

^{1}H NMR (400 MHz) spectrum of N[9]-Trityl-N[6]-tb-adenine in chloroform-*d*.

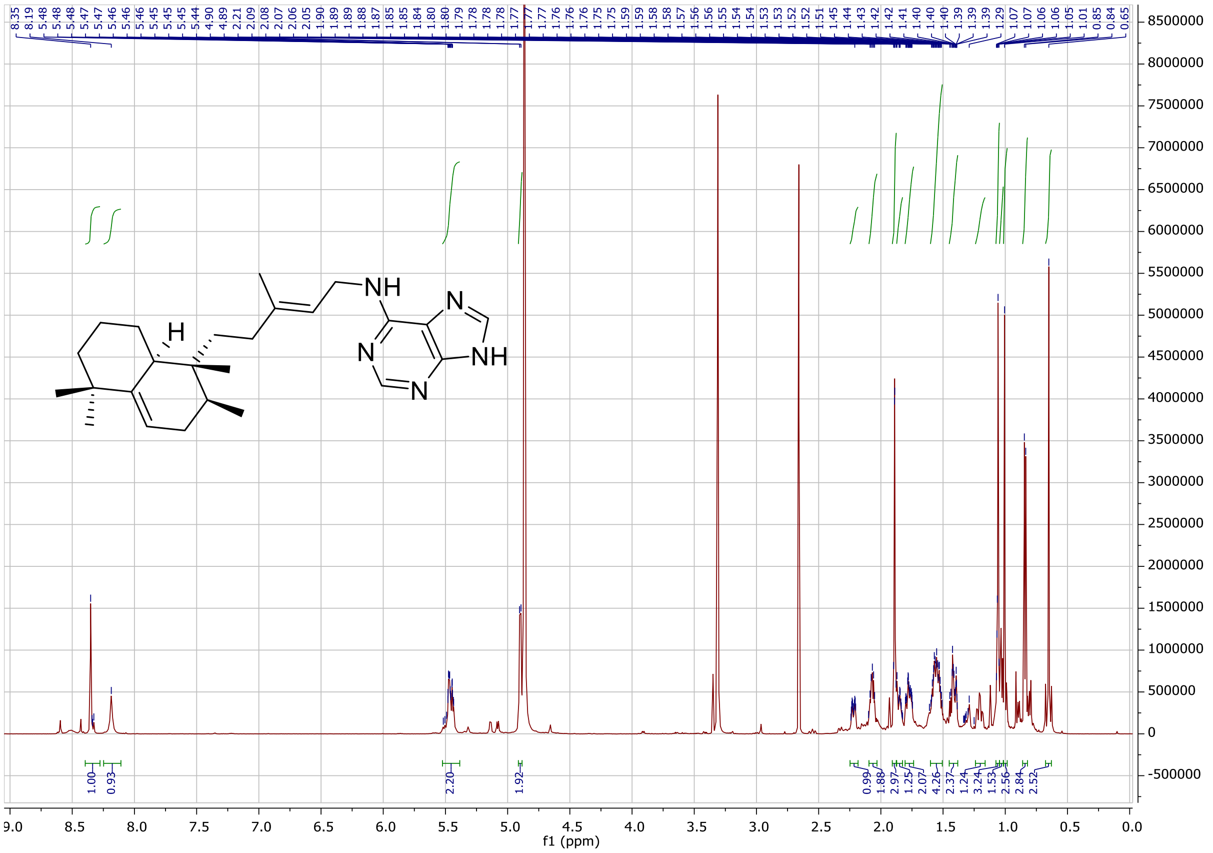

^{1}H NMR (600 MHz) spectrum of N[6]-Tb-adenine **7** in methanol-d_4.

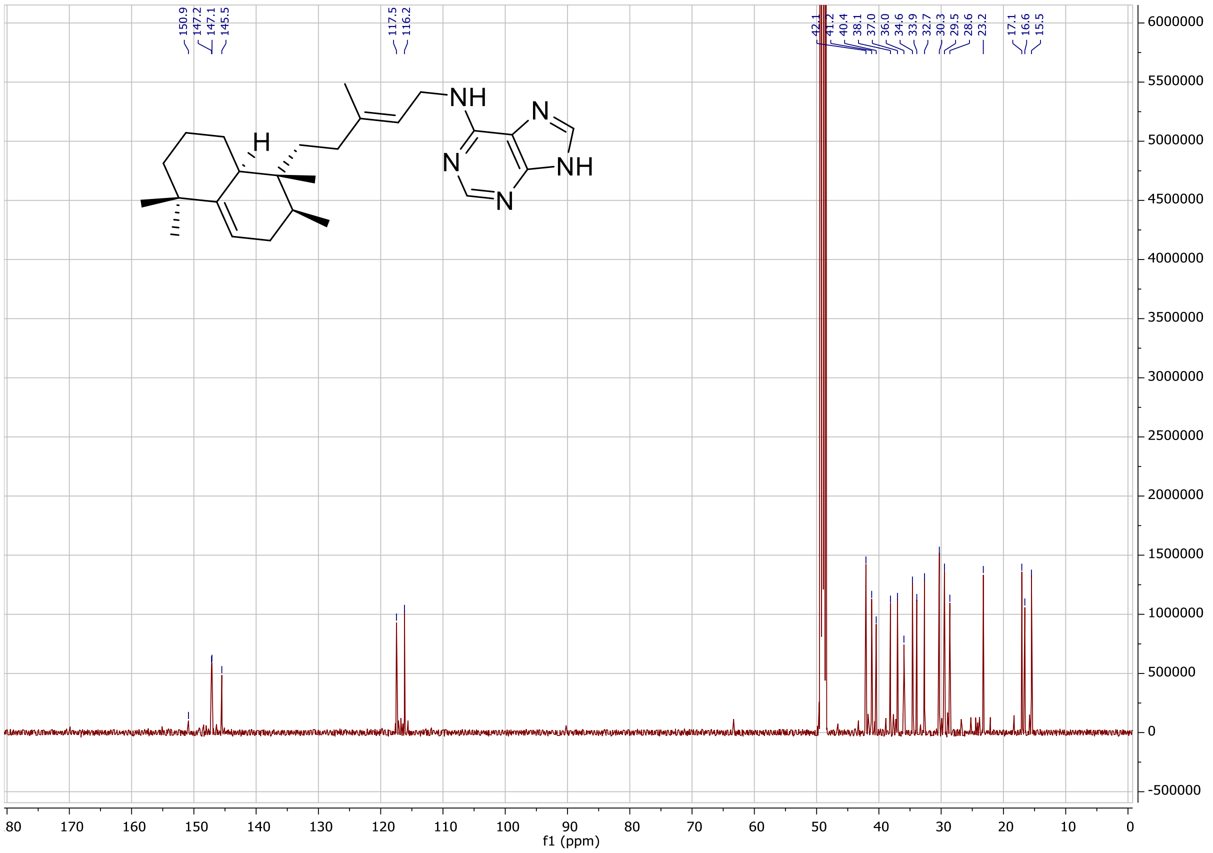

^{1}H NMR (600 MHz) spectrum of N[6]-Tb-adenine **7** in methanol-d_4.

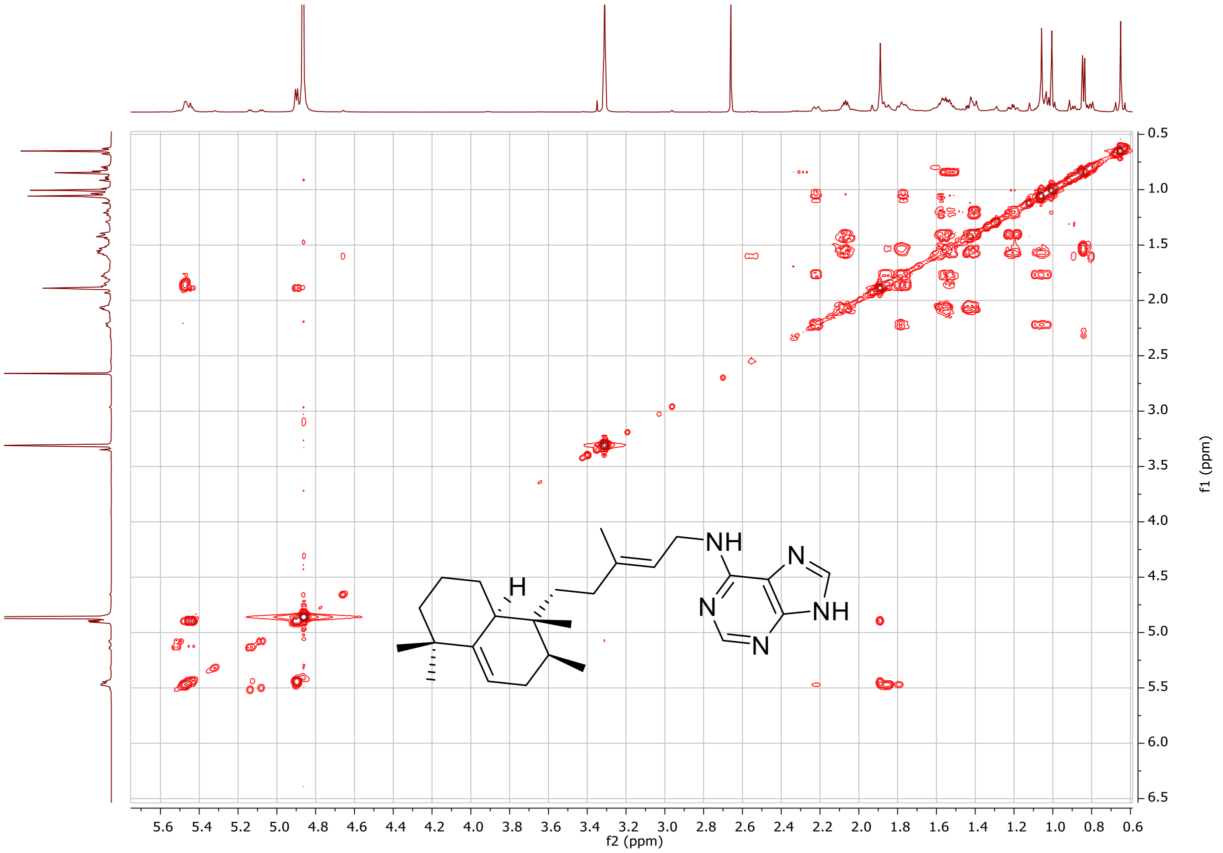

COSY expansion spectrum of N^6-Tb-adenine **7** in methanol-d_4.

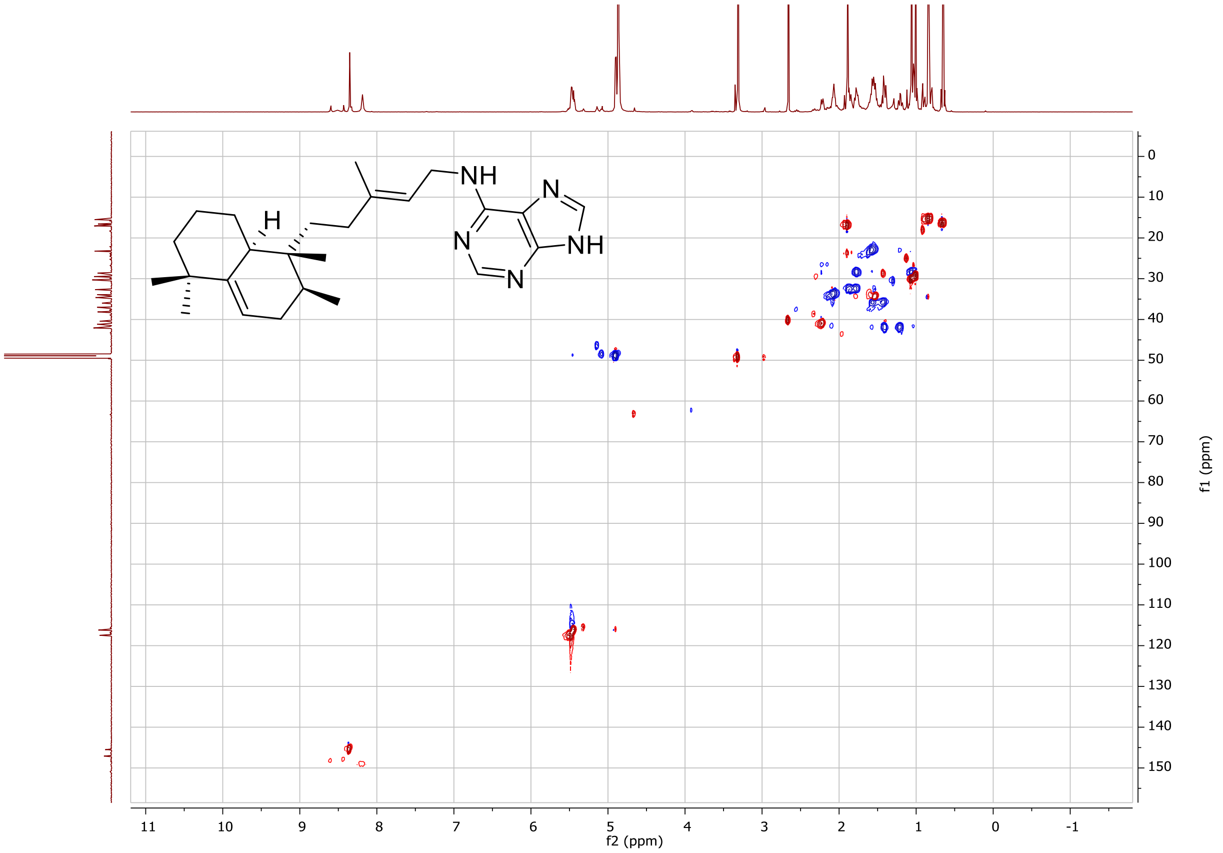

Multiplicity edited HSQC spectrum of N^6-Tb-adenine **7** in methanol-d_4.

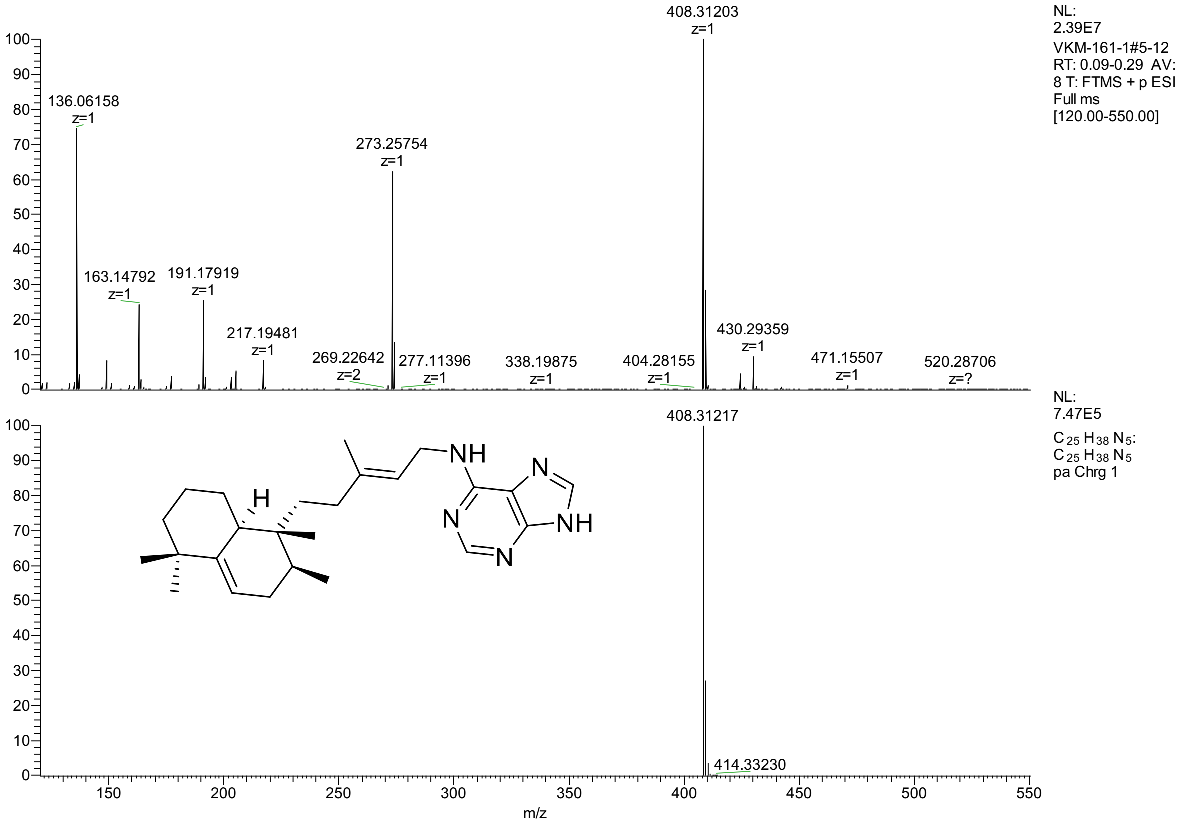

ESI-MS spectrum of N[6]-Tb-adenine **7**.

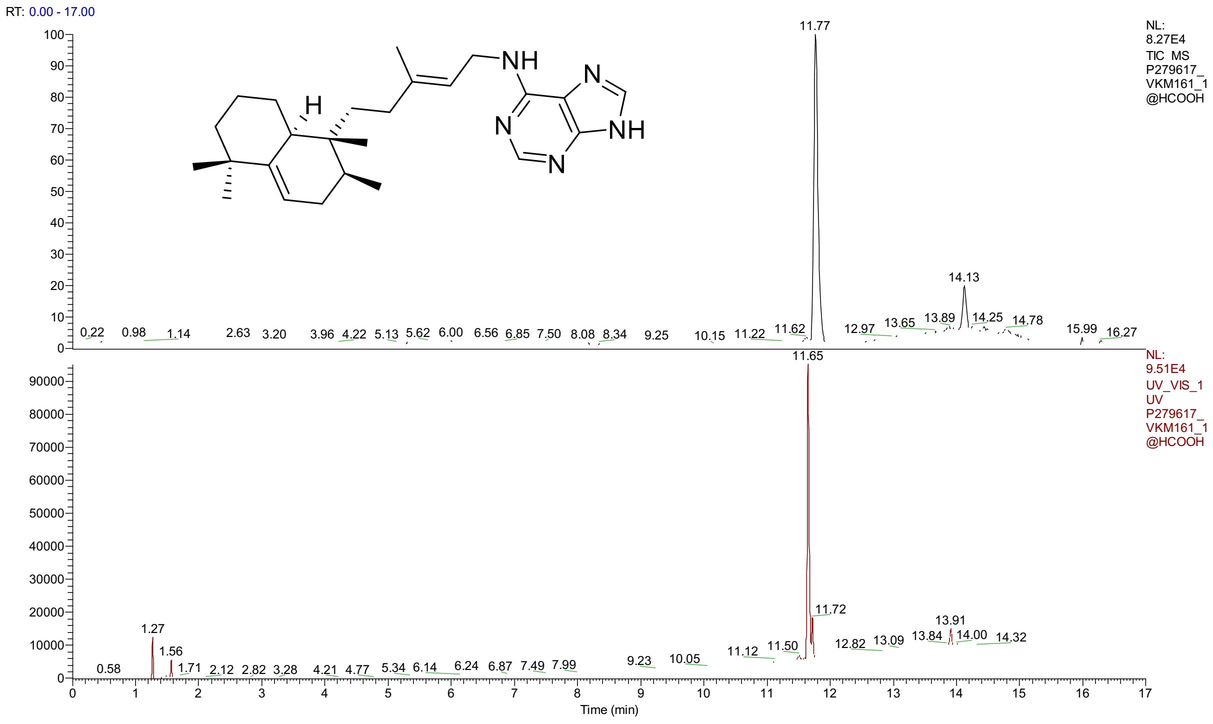

UPLC Chromatogram of N[6]-Tb-adenine **7**.

[1] Hakimelahi, G. H.; Ly, T. W.; Movahedi, A. A. –M, Jain, M. L.; Zakerinia, M.; Davari, H.; Mei, H. –C.; Sambaiah, T.; Moshfegh, A. A.; Hakimelahi, S. *J. Med. Chem.* **2001,** *44,* 3710-3720.

[2] Buter, J., Cheng, T.Y., Ghanem, M., Grootemaat, A.E., Raman, S., Feng, X., Plantijn, A.R., Ennis, T., Wang, J., Cotton, R.N., et al. (2019). *Nat Chem Biol 15*, 889-899.

[3] Buter, J., Heijnen, D., Wan, I.C., Bickelhaupt, F.M., Young, D.C., Otten, E., Moody, D.B., and Minnaard, A.J. (2016). *J Org Chem 81*, 6686-6696.
